# Chemoselective Electrochemical Cleavage of Sulfonimides as a Direct Way to Sulfonamides

**DOI:** 10.1021/acs.joc.3c01932

**Published:** 2024-01-10

**Authors:** Karolína Salvadori, Michal Churý, Jan Budka, Jakub Harvalík, Pavel Matějka, Ludmila Šimková, Pavel Lhoták

**Affiliations:** † J. Heyrovský Institute of Physical Chemistry of Czech Academy of Sciences v.v.i., Dolejškova 2155/3, 182 23 Prague 8, Czech Republic; ‡ Department of Physical Chemistry, University of Chemistry and Technology, Prague (UCTP), Technická 5, 166 28 Prague 6, Czech Republic; § Institute of Chemical Process Fundamentals of Czech Academy of Sciences v.v.i., Rozvojová 135, 165 02 Prague 6, Czech Republic; ∥ Department of Organic Chemistry, UCTP, Technická 5, 166 28 Prague 6, Czech Republic

## Abstract

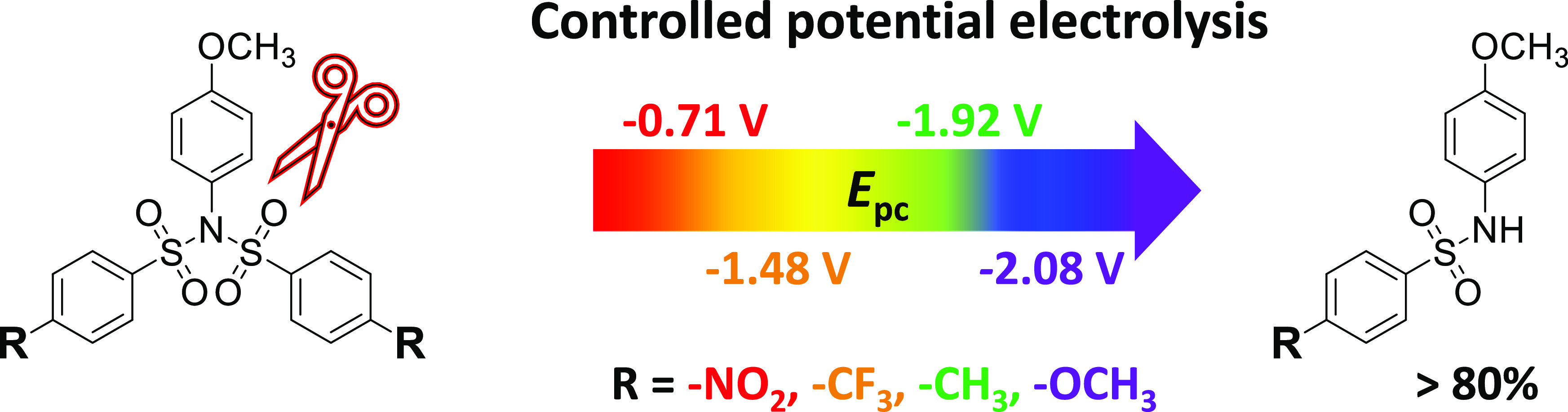

A new method for selective cleavage of sulfonimides into sulfonamides in high yields using a simple electrochemical approach is shown. As revealed by the electrochemical study, the aromatic sulfonimides can be selectively cleaved by electrolysis of the starting compound at a given potential (only −0.9 V vs SCE for the nosyl group). The high chemoselectivity was confirmed by preparative electrolysis, and the results were supported with DFT calculations of a set of substances bearing different sulfonimide functions. Moreover, various experimental setups together with other attempts to simplify the procedure were tested. Finally, the removal of the *p*-nosyl group from the corresponding sulfonimides proceeds smoothly regardless of the number of nosyl groups and the overall shape of the complex molecule. Thus, the method is interesting for use in the field of multifunctional molecules such as calix[*n*]arenes.

## Introduction

Sulfonamides form a very large and important class of compounds with many applications in synthetic and medicinal chemistry. The sulfonamide functional group represents the structural motif of many biologically active substances and drugs, exhibiting antimicrobial, anti-inflammatory, anticancer, hypoglycemic, and protease inhibitory activity, to name just a few.^1−4^ The history of the so-called sulfa-drugs as antibacterial agents is almost 100 years long, and their use in practice is one of the cornerstones of modern medicinal chemistry.

**Figure 1:**
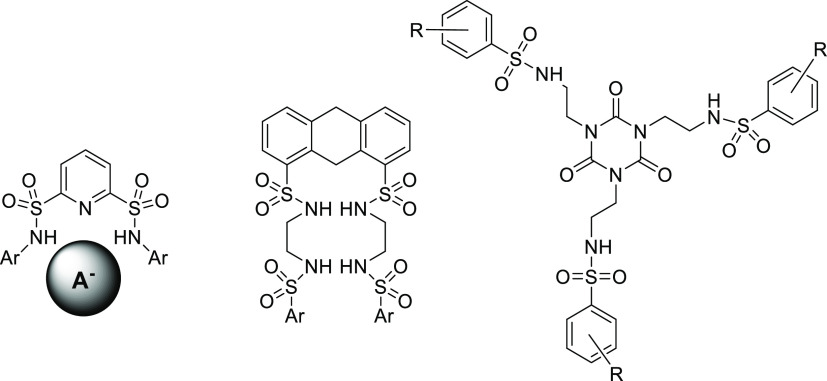
Examples of sulfonamide-based anionic receptors.

In addition to being used for the treatment of a wide range of diseases, sulfonamides have also found their application in supramolecular chemistry, mainly due to the strong hydrogen bonds (HBs) of amidic –NH– hydrogens. The presence of the electron-withdrawing –SO_2_– group makes the NH hydrogens highly acidic, making sulfonamides excellent HB donors. Thus, the high directionality of such HB interactions predisposes the sulfonamide as an important building block in the design of various anion receptors. Indeed, the arrangement of several sulfonamide functions within the molecule can lead to a preorganized cavity capable of anion recognition via cooperative HB interactions (for some examples, see Figure 1).^5−7^ On the other hand, deprotonated sulfonamides can also serve to complex cations, making this group extremely useful.^8^

**Figure 2:**
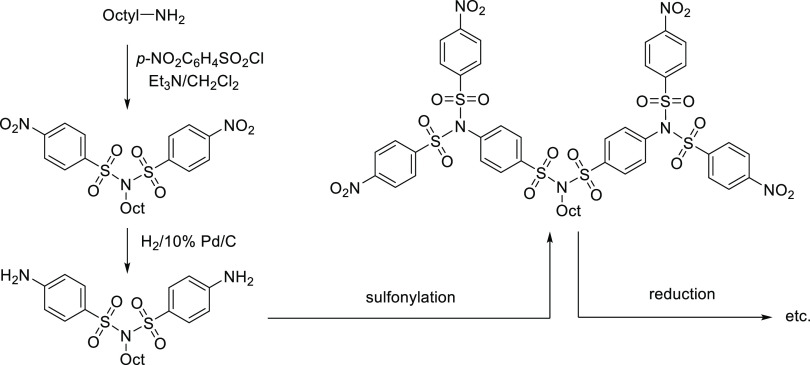
Synthesis of sulfonimide-based dendrimers.

Besides the common sulfonamides, also the products of double substitution–sulfonimides found their application. Rather recently, a new group of sulfonimide-based dendrimers with nosyl groups as repeating units appeared.^9−11^ The application of *p*-nitrobenzenesulfonyl chloride as a starting reagent for the convergent synthesis can lead to higher generations of dendrimers (via reduction of NO_2_ to NH_2_ and repeated sulfonylation), with potential applications in materials science or medicinal chemistry (Figure 2).^12,13^

**Figure 3:**
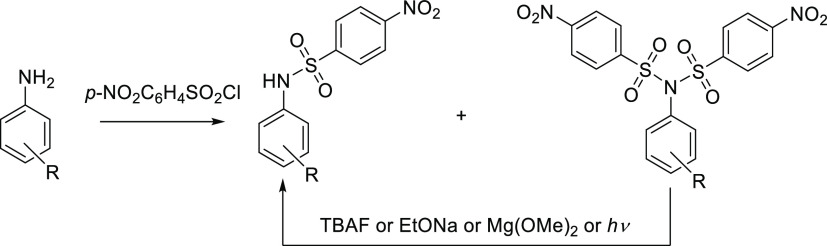
Synthesis of sulfonamides and sulfonimides.

The classical approach to sulfonamides usually involves the nucleophilic substitution of the corresponding sulfonyl chloride derivative with an amine in the presence of base.^2,14^ While aliphatic amines usually provide the desired sulfonamide in high yields, aromatic amines often require elevated temperatures and prolonged reaction times. Despite this, the yields often remain low, and the reaction mixtures require demanding separation of byproducts because in addition to the sulfonamide, an unwanted product formed by double substitution—sulfonimide—is usually present (Figure 3).^15,16^ In our own research, we have encountered the fact that sulfonamide formation is particularly difficult to control for multifunctional molecules with many reactive centers (NH_2_ groups), such as tetraaminocalix[4]arenes.

For these reasons, it would be useful to develop a new method for the selective cleavage of sulfonimide byproducts that would allow higher yields of sulfonamide to be achieved, especially in the case of polyfunctional molecules. Unfortunately, known methods for converting sulfonimides to the corresponding sulfonamides (Figure 3) usually suffer from low yields, harsh reaction conditions, expensive reagents, or low selectivity.^16−18^

However, besides the traditional chemical way of synthesis, the electrochemical approach could be effective. Several examples of electrochemical cleavage of the N–S bond in N-substituted sulfonamides^19−21^ or the O–S bonds^22,23^ in sulfates were published, but only a few precedents are mentioning (albeit marginally) cleavage of sulfonimides.^19,24^

In this paper, we focus on using the electrochemical protocol to prepare sulfonamides from sulfonimides. As we have shown, this approach is simple, highly chemoselective with high yields of sulfonamides and generally applicable to a variety of sulfonyl substituents. In addition, the use of electrochemistry (Pt or GC electrodes) minimizes or completely bypasses the need for various chemical reagents, hitherto required for similar chemical transformations, making our procedure an environmentally acceptable and friendly process.

## Results and Discussion

**Scheme 1:**
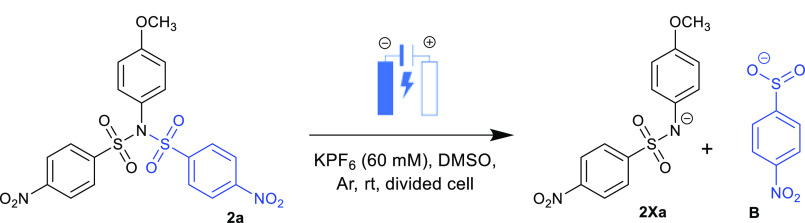
Electrochemical Cleavage of Dinosyl Derivative **2a**

Before using any electrochemical approach for the synthesis of new molecules, first of all, it is essential to explore in detail the electrochemical behavior of the starting compounds, i.e., sulfonimides. At the beginning of our study, we chose di-*p*-nosylimide derivative **2a** as a typical representative of aromatic sulfonimides (Scheme 1). To understand the electrochemical properties of a given compound, one has to detect the potential redox centers in a molecule. In the structure of **2a**, there are two possibilities where the acceptance of an electron could occur—nitro group and sulfonyl motif. The reduction cleavage^25^ of sulfonyl in tosylamides is described as an irreversible two-electron process proceeding at a highly negative potential (approximately −2.0 V); therefore, its contribution seems to be less probable. Then, according to the literature, the most easily reducible motif in molecule **2a** should be the nitro group. The electrochemical behavior of aromatic nitro compounds in polar aprotic solvents is thoroughly documented^26−28^ and usually corresponds to two separate processes. After the first reversible one-electron formation of the radical anion (usually between −0.7 and −1.3 V vs SCE), the second three-electron four-proton irreversible process follows, forming the corresponding hydroxylamine derivative. In the case when the parent molecule contains acidic protons in the structure (in addition to the nitro group), reduction takes place via the so-called autoprotonation mechanism.^29,30^

**Figure 4:**
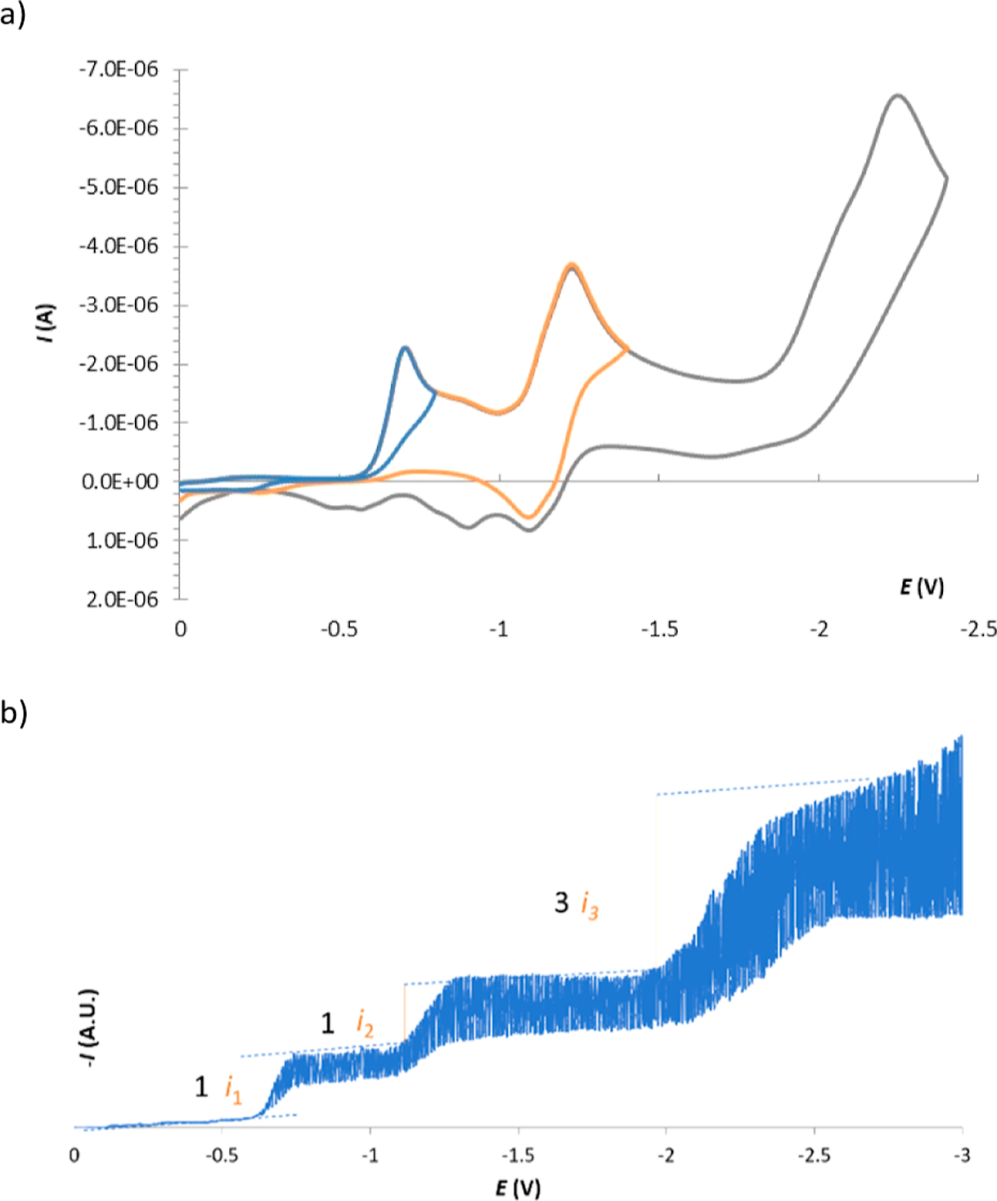
(a) Cyclic voltammetry **2a** (1 × 10^–3^ M) in DMSO (0.1 M TBAPF_6_) on the mercury electrode at a scan rate 100 mV·s^–1^ (gradually increasing potential range); (b) DC-polarography of **2a** (5 × 10^–4^ M) in DMSO (0.1 M TBAPF_6_) with an indicated ratio of limiting currents. The potentials are compared to SCE, the counter electrode was Pt.

Based on this knowledge, we have carried out the electrochemical reduction study of sulfonimide **2a**. To elucidate electrochemical properties, a combination of steady-state (DC-polarography) and dynamic (cyclic voltammetry) methods was applied. Regardless of material of the working electrode used (mercury electrode, glassy carbon electrode, or Pt disk electrode), the reduction mechanism is the same. The records obtained (Figures 4a and S85–S87) showed three well-separated reduction steps with the corresponding limiting currents in a 1:1:3 ratio (measured by DC-polarography, see Figure 4b). As expected, the reduction of nitro groups proceeds. The formation of a reversible couple(s) of nitro groups is located at around −1.2 V, and their further reduction proceeds in the third reduction step (−2.3 V) [the third reduction step is not observed on the Pt electrode because of the potential window (up to −2.0 V vs SCE)]. This is not surprising as it reflects the normal electrochemical behavior of NO_2_ groups. However, the presence of the first irreversible step at −0.71 V (peak potential) was completely unexpected and remained unknown.

To clarify this issue, a preparative (bulk) electrolysis in DMSO-*d*_6_ has been carried out on a 20 mg scale in an H-type cell (anodic and cathodic parts are separated). The divided cell was used to avoid mixing of products formed on working and counter (auxiliary) electrodes and/or to suppress the undesirable reoxidation of expected product(s). The applied potential was set up to −0.9 V, ca. 100 mV behind the first irreversible reduction step and before the second reduction step. The electrolysis continued until the current exponentially decreased (see Figure S90). Quantitative conversion (the amount of the transformed substrate) is directly proportional to the total charge consumed, which corresponds to two electrons passed through. Then, ^1^H NMR, UV–vis, and HRMS spectra of the crude mixture were recorded (Figures S91–S93), providing the evidence for electrochemical splitting of the molecule. During the electrolysis, the red–colored reaction mixture formed containing the *p*-nitrobenzenesulfinate anion **B** (*m*/*z* = 186) together with anionic species **2Xa** (*m*/*z* = 307) (Scheme 1). From this mixture, the sulfonamide **2Aa** was isolated in 85% yield by preparative TLC chromatography after an acidic workup.

Based on the knowledge gained, a preliminary mechanism running in the first reduction step was proposed. Although the nitro group should be generally the most easily electrochemically reducible motive within the **2a**, it is also a strong electron-withdrawing substituent. This action results in an electron-deficient area located at the sulfur atoms of the sulfonimide group.

**Figure 5:**
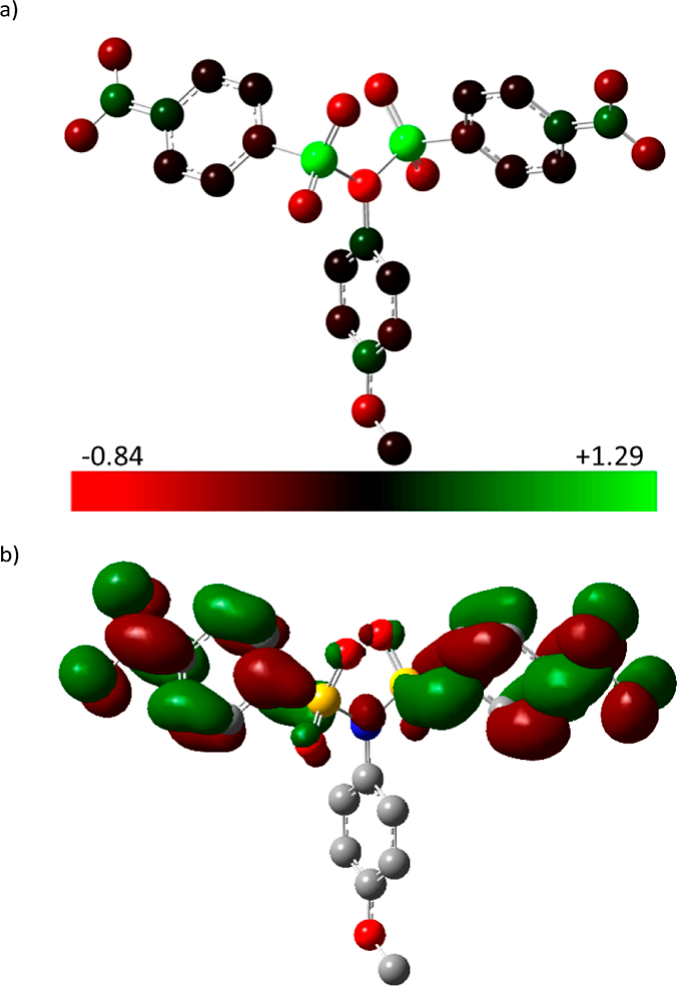
DFT calculations showing: (a) charge distribution (electron deficient = green); (b) LUMO orbitals of derivative **2a**.

**Scheme 2:**
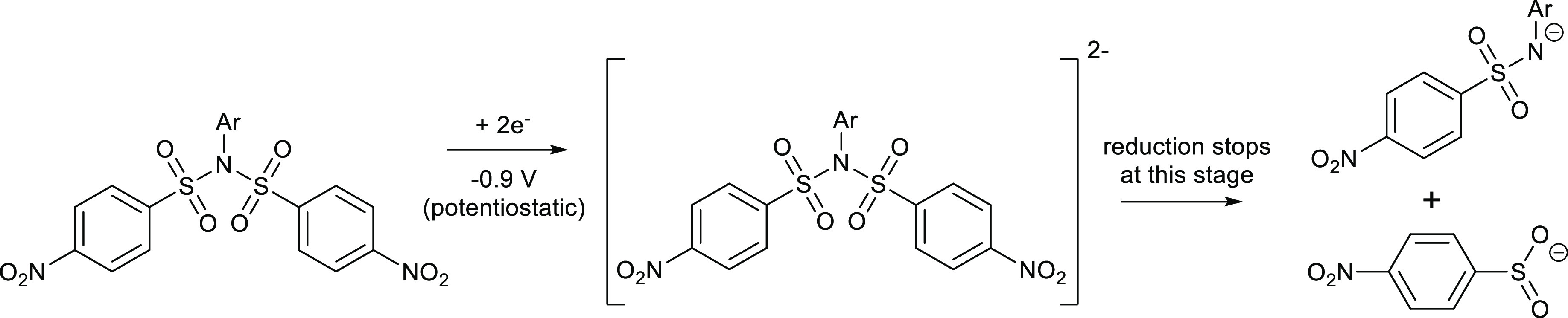
Tentative Mechanism of a Chemoselective Electrochemical Reductive Cleavage of Nosylimides

This assumption is also supported by DFT calculations showing the charge distribution in **2a**. The most electron-deficient area in the molecule (Figure 5a, in green color) corresponds to the sulfur atoms of the imide group. Similar conclusions can be drawn by the analysis of the LUMO orbitals as the place of the most probable electron attack. The LUMO orbitals are mainly localized at both nosyl groups (Figure 5b). Therefore, the molecule can accept two electrons during the first irreversible process (Scheme 2).^31^ This mechanism was supported by additional experiments disproving the alternative ECE mechanism.^32^ The cleavage of intermediate dianionic species gives finally the *p*-nitrobenzenesulfinate anion **B** as a leaving group and the anion of the remaining sulfonamidic product.^21^ Since the entire process takes place under potentiostatic conditions (at −0.9 V), the sulfonamide product cannot react further because the anions formed would need a much more negative potential for their further transformation. Hence, the reduction stops at the sulfonamide stage, thus representing a highly chemoselective process.

**Scheme 3:**
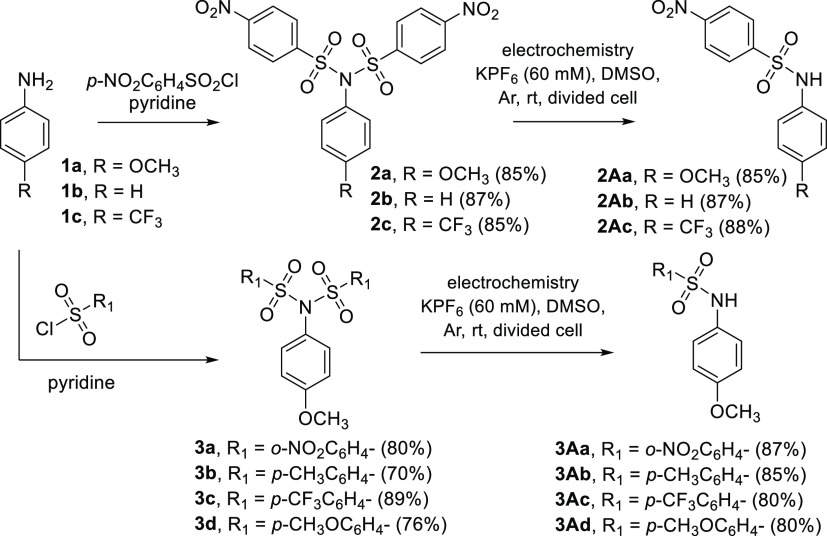
Synthesis of Various Sulfonylimide Series and Their Electrolysis to the Corresponding Sulfonamides

To study the general applicability of this method, a series of nosyl derivatives **2a–2c** bearing different substituents in the *para* position of the aniline moiety were prepared (Scheme 3) starting from anilines **1a–1c**. The electrochemical study confirmed the same cleavage mechanism in all cases, while the cathodic peak (splitting) potentials (−0.59 to −0.71 V) were only little affected by the type of substitution on aniline (EWG vs EDG substituents). The preparative electrolysis at the applied potential set behind the first peak potential (corresponding to splitting) carried out on a 20 mg scale confirmed the formation of amide derivatives **2Aa–2Ac** in very high yields (>85%, Table S3).

Similarly, the imides **3a–3d** bearing two identical sulfonyl groups (different from *para*-nosyl) were prepared by the reaction of *p*-methoxyaniline **1a** with the corresponding aromatic sulfonyl chlorides (**3a**–**3d**) in pyridine (Scheme 3). From the electrochemical point of view, *ortho*-nosyl derivative **3a** behaves in the same way as *para*-nosyl isomers, and the corresponding splitting potential is −0.74 V. On the other hand, the absence of the nitro group in compounds **3b**–**3d** results in a significant shift of the cathodic peak potentials toward the negative region. The size of this shift depends on the electron-accepting or electron-donating properties of the substituents. Thus, *p*-CF_3_ group (**3c**, acceptor) gave the value of −1.48 V, *p*-CH_3_ (**3b**, donor) showed the peak at −1.92 V, and *p*-OCH_3_ (**3d**, strong donor) was found at −2.08 V.

Such a shift of the splitting potential obtained for both donors could already cause some limitations for electrolysis related to the solvent potential window^33^ for the Pt electrode (up to −2.0 V) or problems with the use of the potassium-based supporting electrolyte.^34^ On the other hand, these issues can be overcome by using different working electrodes (such as glassy carbon or mercury electrode) with the TBA supporting electrolyte. Regardless of the different potentials, smooth cleavage of the original molecules occurred again in all cases, as proven by the preparative-scale electrolysis (Scheme 3, Table 1).

**Table 1:** Comparison of Substituent Effect (in Symmetrically Substituted Compounds **2a–c** and **3a–d**)^a^ on the Shift of Splitting Potentials

compound^b^	*E*_pc_ (V)^c^	applied potential (V)^d^	yields (%)
**2a**	–0.71	–0.9	85
**2b**	–0.67	–0.9	87
**2c**	–0.59	–0.8	88
**3a**	–0.74	–0.9	87
**3b**	–1.92	–2.0	85
**3c**	–1.48	–1.6	80
**3d**	–2.08	–2.2	80

a All reactions were carried out with 4 mL of solution (*c* = 10 mM) corresponding to ca. 20 mg of starting imides.

b For the corresponding structures, see Scheme 3.

c Cathodic peak potential corresponding to the first irreversible (splitting) step.

d Potential applied during electrolysis.

**Scheme 4:**
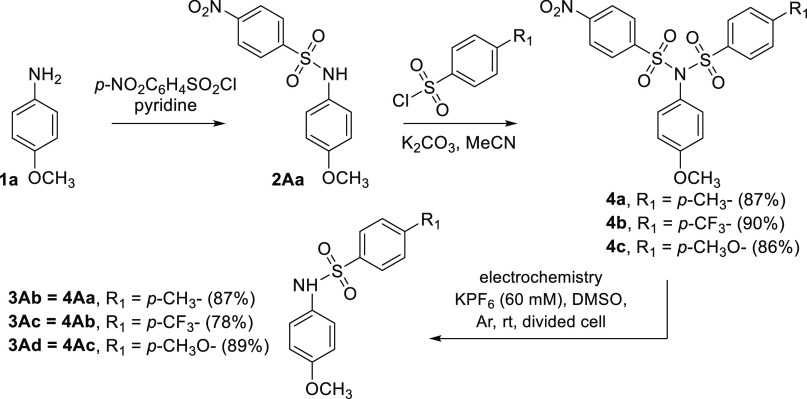
Synthesis of Sulfonylimides Bearing Different Sulfonyl Groups and Their Selective Electrolysis to the Corresponding Amides

Based on previous experiments, derivatives **4a**–**4c** bearing two different sulfonyl groups, one of which was always the *para*-nosyl group, were also prepared (Scheme 4). The value of the cleavage potential was confirmed by an electrochemical study similar to that of **2a**. As expected, the electrolysis carried out at −0.9 V smoothly provided the products **4Aa–4Ac** with the *para*-nosyl groups being removed selectively.

**Figure 6:**
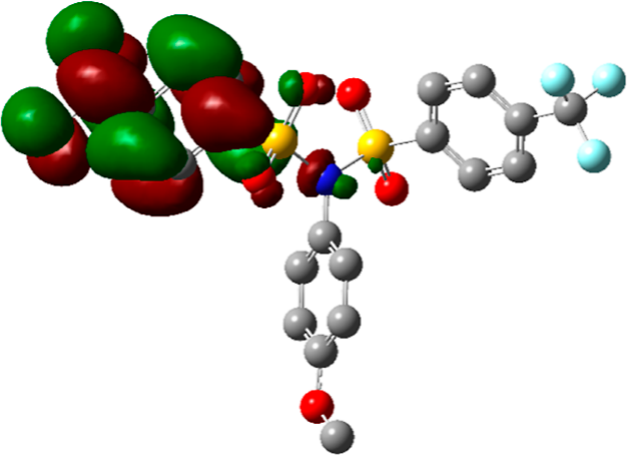
LUMO orbitals in compound **4b**.

These experiments show that the nosyl group can be used as a protecting group on the sulfonamide nitrogen because its removal is very simple, highly selective, and takes place at the least negative potential by far. Interestingly, even the comparison with the strongly electron-withdrawing –CF_3_ substituent sounds clear for the –NO_2_ group [compare the splitting potentials for **2a** (NO_2_, −0.71 V) and **3c** (−CF_3_, −1.48 V)]. These conclusions are further supported by DFT calculations of compound **4b** (bearing both *p*-NO_2_ and *p*-CF_3_ functions). The LUMO orbital is strictly located on the nosyl part of the molecule (see Figure 6), making this fragment an ideal electron acceptor, and finally, a good leaving group (as the corresponding sulfinate).

**Scheme 5:**
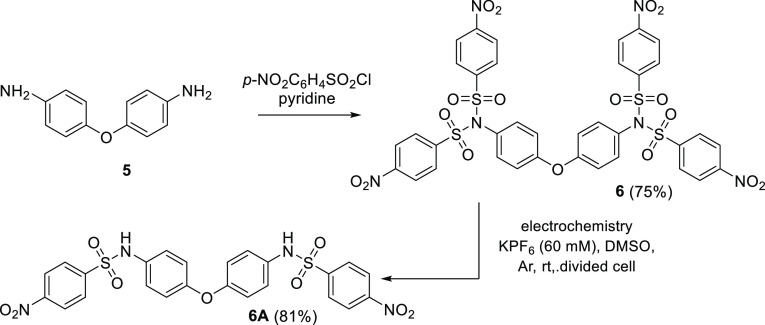
Selective Electrolysis of the Complex Sulfonylimide **6** to the Corresponding Amide **6A**

The nosylation of commercially available bis-aniline **5** provided the corresponding bis-imide **6** in very good yield (Scheme 5). This derivative served as a model substance for systems bearing a larger number of nosyl groups. Compound **6** behaves in the same way as the monoimides. It is reduced in three well-separated reduction steps (see ESI, Figure S107). Electrolysis performed at −0.8 V (the potential behind the potential of the first reduction step = splitting step) afforded the expected product **6A** in good yield (81%), demonstrating that the selective removal of the nosyl (protective) group works even in more complex systems.

**Scheme 6:**
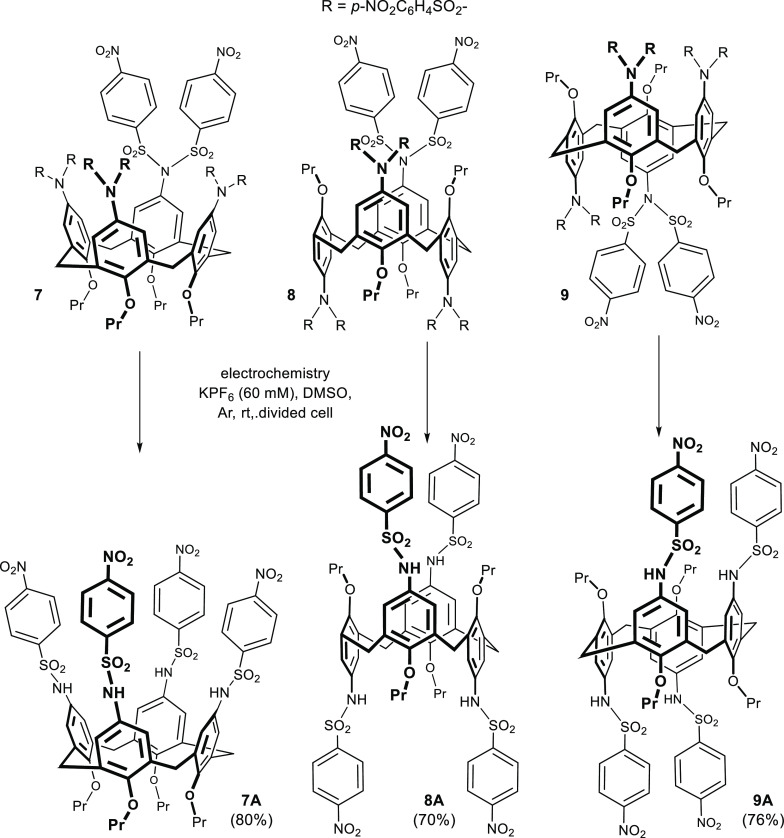
Application of Selective Removal of Nosyl Groups in the Chemistry of Calix[4]arenes

To support this claim, the series of studied compounds was expanded to include nosylated calixarenes. Calix[*n*]arenes^35,36^ are a well-known family of macrocyclic compounds that have found diverse applications in modern supramolecular chemistry due to their complexation properties and the almost limitless possibility of derivatization of the basic skeletons. Calix[4]arenes, in particular, are important starting points for the design of various molecular receptors^37,38^ due to their ability to fix different 3D conformations (atropisomers) through the lower rim alkylation. As we have mentioned in the introduction, during the work on the preparation of tetrakis-sulfonamides **7A**-**9A**, we encountered difficulties in isolating the pure products (see Figure S75 for crude NMR spectra of **7A**). Hence, encouraged by the above-described results, we decided to use the new electrochemical removal of the nosyl functional group in calix[4]arene chemistry. This should not only demonstrate the applicability of this method for multifunctional molecules but also reveal whether there are any restrictions in terms of different 3D shapes (conformations) of the molecules (Scheme 6).

The starting tetraimides **7**–**9** immobilized in three different conformations (*cone*, *1,3-alternate*, and *1,2-alternate*) were obtained by the reaction of the corresponding tetraamines with an excess of 4-nitrobenzenesulfonyl chloride in dichloromethane in the presence of Et_3_N. After one-week reflux, the products were isolated by a simple precipitation with methanol in 54–58% yields. The subsequent experiments by standard electrochemical methods confirmed that all three compounds **7**–**9** exhibited the same behavior during the reduction process, possessing three well-separated reduction steps with the first one (around −0.7 V) being irreversible (see Figures S108–S110).

The corresponding preparative electrolysis of the *cone* conformer **7** was carried out under standard conditions (−0.9 V, mercury pool electrode). Subsequent acidic workup of the crude reaction mixture and final purification using preparative TLC (silica gel) provided the *cone* amide **7A** in 80% yield. As expected from the electrochemical study, the preparative electrolysis of **8** and **9** proceeded identically, and the amides **8A** and **9A** were isolated in very high yields (>70%). This demonstrates that regardless of the calixarene conformation, the nosyl groups can be selectively removed from sulfonimide moieties.

All the electrolyses mentioned so far have been carried out in divided cells with mercury pool used as the working electrode. However, preparative (bulk) electrolysis can be performed on many types of apparatus, so we decided to test the general applicability of reductive cleavage by modifying the experimental setup. The first parameter studied in the electrolysis of **2a** was the material of the working electrode. In our case, we again chose three different materials: mercury, glassy carbon, or platinum. The measurement did not show the influence of the material of the working electrode. However, the effect of its size on the reaction rate should be expected.

To simplify the setup, an electrochemical cell without a separator was also tested. For this purpose, two different materials of counter (auxiliary) electrodes were analyzed. In an undivided cell equipped with a Pt counter electrode, the competitive oxidation process is not suppressed. In this case, **2Aa** was isolated in only 60% yield, and the crude mixture contained impurities (detected by TLC), probably related to uncontrolled oxidation^39^ of deprotonated species **2Xa** (Figure S112). However, as we have shown, this problem can be overcome by using a magnesium counter electrode that dissolves during electrolysis to compensate for oxidation (Table 2).

**Table 2:** Comparison of Different Experimental Setups during Electrolysis of **2a** (20 mg, with 60 mM KPF_6_ in DMSO)^a^

electrode	material	cell	time	yield (%)
working	Hg	divided	2 h 30 min	85
	Pt		2 h 15 min	83
	GC		3 h	80
counter	Pt	undivided	3 h 5 min	60
	Mg		2 h 45 min	78

a The applied potential was set to −0.9 V.

After demonstrating the usefulness of the nosyl group as an electrochemically easily cleavable function, we attempted to further simplify the entire synthetic procedure. We asked ourselves, is it really necessary to isolate the unwanted sulfonimide from the desired sulfonamide before using electrochemical cleavage? In other words, is it possible to take a crude reaction mixture containing sulfonamide and sulfonimide and perform electrolysis to produce only the desired sulfonamide product?

To answer this question, the sulfonamide **2Ab** was tested in a mixture with its undesirable byproduct **2b**. The standard electrochemical methods (Figure S113) applied to pure sulfonamide **2Ab** revealed the reduction corresponding to the autoprotonation mechanism due to the presence of highly acidic sulfonamidic hydrogen (Scheme S1). Unfortunately, the first step (−0.91 V peak potential) is very close to our set electrochemical potential for the cleavage of sulfonimide **2b** (−0.90 V applied potential).

The actual value of the applied potential can of course be shifted as needed. In this case, it would be possible to go as low as −0.7 V since the actual splitting peak potential for **2b** is −0.65 V. However, there is a much simpler solution. The introduction of a base into the crude reaction mixture (where applicable) should lead to deprotonation of the acidic sulfonamidic hydrogen which shifts the reduction of **2Xb** to much more negative potential as the autoprotonation step cannot occur. Indeed, the addition of TBAOH or KOH (approximately 1 equiv to **2Ab**) to a mixture of **2Ab** and **2b** (1:1 molar ratio) confirmed that the electrolysis could be carried out under the usual setup (−0.90 V) even with the crude reaction mixture. This is very advantageous regarding the possible losses caused by the isolation of sulfonimides from reaction mixtures, but also in connection with the preparation of multifunctional molecules, where the synthesis of fully nosylated derivatives could be for some reason complicated.

## Conclusions

As revealed, the aromatic sulfonimides can be selectively cleaved to the corresponding sulfonamides using electrolysis of the starting compound at a given potential (−0.9 V vs SCE for the nosyl group). The procedure is independent of the material of the working electrode, the possibility of using an auxiliary electrode dissolved during electrolysis makes this method undemanding to the equipment. Preparative electrolysis of a series of substances bearing different sulfonimide functions confirmed that this procedure is highly chemoselective as undesired cleavage of the sulfonamide bond was never observed. Moreover, the removal of the *p*-nosyl group from the corresponding imides proceeds smoothly regardless of the overall shape of the molecule, which makes this method attractive for applications in the field of multifunctional molecules such as calix[n]arenes.

## Experimental Section

### General Experimental Procedures

All chemicals were purchased from commercial sources and used without further purification. The solvents used for synthesis and chromatography were purchased from commercial sources and were distilled before use. Anhydrous solvents were dried by standard procedures; pyridine was stored above NaOH(s), and ACN was stored over molecular sieves. The reaction progress during synthesis was monitored by analytical TLC, carried out on foil sheets coated with silica gel containing a fluorescent indicator −60 F_254_ (Merck). Separation of products after electrolysis was provided by using self-made preparative TLC plates carried out on glass plates (10 × 20 cm) covered by silica gel 60 PF_254_ (Merck). Melting points were measured on Heiztisch Mikroskop Polytherm A (Wagner & Munz), and they are not corrected. The ^1^H (400.1 MHz), ^13^C (100.6 MHz), and ^19^F (376.5 MHz) NMR spectra were recorded using a Bruker Avance 400 spectrometer (Bruker Biospin, Rheinstetten, Germany) at 25 °C. Used solvents (DMSO-*d*_6_, chloroform-*d*) were stored over molecular sieves. The ^1^H and ^13^C NMR spectra were referenced to the line of the solvent (δ/ppm; δ_H_/δ_C_: DMSO-*d*_6_, 2.50/39.52, chloroform-*d*, 7.26/77.16). The ^19^F spectra were referenced to the line of standard hexafluorobenzene (δ_F_/ppm; −163.00). The FTIR analysis was performed on a Nicolet 6700 spectrometer (Thermo-Nicolet, USA) connected with a GladiATR diamond ATR adapter (PIKE, USA), reflectance measurement, DTGS KBr detector, with the following parameters: spectral range: 4000–400 cm^–1^, resolution: 4 cm^–1^, number of accumulations: 64, and apodization: Happ-Genzel. The spectra were collected and processed by Omnic 9 (Thermo-Nicolet Instruments Co., USA) including baseline correction. The high-resolution mass spectra (HRMS) were measured on a MicrOtof III spectrometer (Bruker Daltonic, Bremen, Germany) with electrospray (ESI) and atmospheric pressure chemical ionization (APCI) source in the positive or negative mode. For calibration of accurate masses, ESI-APCI Low Concentration Tuning Mix (Agilent, Santa Clara, CA, USA) was used. The samples were delivered into the ion source in a methanol solution. The UV–vis spectra were acquired on a dual beam UV-1800 spectrophotometer Shimadzu (Scinteck Instruments, USA) in the range of 260–800 nm with a 1 nm step. The baseline was determined by measuring the DMSO electrolyte solution (60 mM KPF_6_ in DMSO) in the cuvette with a 1 mm path length. The samples for these experiments were diluted to the concentration of 1 × 10^–4^ mol/L.

### Synthetic Procedures

#### *N*-(4-Methoxyphenyl)-4-nitrobenzenesulfonamide **2Aa**

*p*-Anisidine **1a** 0.25 g (2.0 mmol) was dissolved in pyridine (35 mL), and an equimolar amount of *p*-nosyl chloride (0.45 g, 2.0 mmol) was added under stirring. The reaction was stirred at ambient temperature, and the conversion was monitored by TLC. After completion of the reaction, aqueous solution of HCl was added to reach a pH value of cca 2, and the product was extracted into ethyl acetate. The organic layer was dried over MgSO_4_ and evaporated at the reduced pressure. The crude product **2Aa** was obtained as an orange powder (0.55 g) in 88% yield and was used in the next step without further purification.

^1^H NMR (400 MHz, DMSO-*d*_6_): δ 10.23 (s, 1H, N*H*); 8.36 (d, 2H, Ar*H*, *J* = 8.9 Hz); 7.91 (d, 2H, Ar*H*, *J* = 8.9 Hz); 6.97 (d, 2H, Ar*H*, *J* = 9.0 Hz); 6.82 (d, 2H, Ar*H*, *J* = 9.0 Hz); 3.67 (s, 3H, –OCH_3_) ppm. The spectrum is in agreement with the literature.^40^

#### Compounds **2**, **3**, and **6**

A corresponding aromatic amine **1** or **5** (2.0 mmol) was dissolved in pyridine (50 mL) and stirred. To the mixture, 2.2–5.0 equiv (4.4–10 mmol) of corresponding sulfonyl chloride were added. The reaction was stirred at ambient temperature, and the conversion was monitored by TLC. After completion of the reaction, the pyridine was converted to water-soluble pyridinium salt (the crude mixture was poured into aqueous solution of HCl), and the product was extracted into ethyl acetate (3 × 30 mL). To eliminate unreacted sulfonamides, the collected organic phase was washed with 1 M solution of NaOH. The organic layer was dried over MgSO_4_ and evaporated at the reduced pressure.

##### *N*-(4-Methoxyphenyl)-4-nitro-*N*-((4-nitrophenyl)sulfonyl)benzenesulfonamide **2a**

The product was obtained as a white powder (0.85 g) in 85% yield, mp 245–248 °C. ^1^H NMR (400 MHz, DMSO-*d*_6_): δ 8.52 (dd, 4H, Ar*H*, *J*_1_ = 9.0 Hz, *J*_2_ = 2.0 Hz); 8.11 (dd, 4H, Ar*H*, *J*_1_ = 9.0 Hz, *J*_2_ = 2.0 Hz); 7.06–6.98 (m, 4H, Ar*H*); 3.81 (s, 3H, –OCH_3_) ppm. ^13^C{^1^H} NMR (100 MHz, DMSO-*d*_6_): δ 161.0; 150.9; 143.2; 132.7; 129.8; 125.0; 124.6; 115.1; 55.6 ppm. IR 3102; 3072; 3042; 3004; 2961; 2932; 2857; 1606; 1586; 1538; 1504 cm^–1^. HRMS (ESI-TOF) *m*/*z*: [M + Na]^+^ calcd for C_19_H_15_N_3_O_9_S_2_Na, 516.0141; found, 516.0138. The spectral characterization is identical to that previously reported.^41^

##### 4-Nitro-*N*-((4-nitrophenyl)sulfonyl)-*N*-phenylbenzenesulfonamide **2b**

The product was obtained as a yellowish powder (0.82 g) in 87% yield. mp 260–264 °C. ^1^H NMR (400 MHz, DMSO-*d*_6_): δ 8.52 (d, 4H, Ar*H*, *J* = 9.0 Hz); 8.12 (d, 4H, Ar*H*, *J* = 9.0 Hz); 7.60 (t, 1H, Ar*H*, *J* = 7.4 Hz); 7.51 (dd, 2H, ArH, *J*_1_ = 8.0 Hz, *J*_2_ = 7.4 Hz); 7.15 (d, 2H, ArH, *J* = 8.0 Hz) ppm. ^13^C{^1^H} NMR (100 MHz, DMSO-*d*_6_): δ 151.0; 143.1; 132.6; 131.3; 131.2; 130.0; 129.9; 125.1 ppm. IR 3110; 3073; 3040; 2872; 1608; 1528 cm^–1^. HRMS (APCI-TOF) *m*/*z*: [M + H]^+^ calcd for C_18_H_14_N_3_O_8_S_2_, 464.0217; found, 464.0219. The spectral characterization is identical to that previously reported.^42^

##### 4-Nitro-*N*-((4-nitrophenyl)sulfonyl)-*N*-(4-(trifluoromethyl)phenyl)benzenesulfonamide **2c**

The product was obtained as a white powder (0.93 g) in 85% yield. mp 285–287 °C. ^1^H NMR (400 MHz, DMSO-*d*_6_): δ 8.52 (dd, 4H, Ar*H*, *J*_1_ = 9.0 Hz, *J*_2_ = 2.6 Hz); 8.15 (dd, 4H, Ar*H*, *J*_1_ = 9.0 Hz, *J*_2_ = 2.6 Hz); 7.91 (d, 2H, Ar*H*, *J* = 8.4 Hz); 7.42 (d, 2H, Ar*H*, *J* = 8.2 Hz) ppm. ^13^C{^1^H} NMR (100 MHz, DMSO-*d*_6_): δ 151.1; 142.7; 136.2; 132.5; 131.2 (q, *J* = 32.2 Hz); 130.0 Hz; 127.2 (q, *J* = 3,5 Hz); 125.2; 123.6 (q, *J* = 271 Hz) ppm. ^19^F NMR (376 MHz, DMSO-*d*_6_): δ −61.39 ppm. IR 3127; 3110; 3077; 3043; 2872; 1609; 1533 cm^–1^., HRMS (ESI-TOF) *m*/*z*: [M + Na]^+^ calcd for C_19_H_12_F_3_N_3_O_8_S_2_Na, 553.9910; found, 553.9904.

##### *N*-(4-Methoxyphenyl)-2-nitro-*N*-((2-nitrophenyl)sulfonyl)benzenesulfonamide **3a**

The product was obtained as a white powder (0.80 g) in 80% yield. mp 212–215 °C. ^1^H NMR (400 MHz, DMSO-*d*_6_): δ 8.21 (d, 2H, Ar*H*, *J* = 7.9 Hz); 8.08–7.96 (m, 6H, Ar*H*); 7.15 (d, 2H, Ar*H*, *J* = 9.0 Hz); 6.98 (d, 2H, ArH, *J* = 9.0 Hz); 3.79 (s, 3H, –OCH_3_) ppm. ^13^C{^1^H} NMR (100 MHz, DMSO-*d*_6_): δ 161.1; 147.7; 137.0; 133.2; 132.8; 131.8; 129.2; 124.7; 123.2; 114.8; 55.6 ppm. IR 3098; 3082; 3028; 2985; 2950; 2907; 1603; 1586; 1541; 1503 cm^–1^. HRMS (ESI-TOF) *m*/*z*: [M]^+^ calcd for C_19_H_15_N_3_O_9_S_2_, 493.0244; found, 493.0256.

##### *N*-(4-Methoxyphenyl)-4-methyl-*N*-tosylbenzenesulfonamide **3b**

The product was obtained as a beige powder (0.61 g) in 70% yield. mp 160–163 °C. ^1^H NMR (400 MHz, DMSO-*d*_6_): δ 7.67 (d, 4H, Ar*H*, *J* = 8.4 Hz); 7.48 (d, 4H, Ar*H*, *J* = 8.1 Hz); 6.96 (d, 2H, Ar*H*, *J* = 9.0 Hz); 6.88 (d, 2H, Ar*H*, *J* = 9.0 Hz); 3.78 (s, 3H, –OCH_3_); 2.45 (s, 6H, –CH_3_) ppm. ^13^C{^1^H} NMR (100 MHz, DMSO-*d*_6_): δ 160.4; 145.3; 135.8; 132.5; 129.9; 128.0; 125.8; 114.6; 55.5; 21.2 ppm. IR 3069; 3038; 2923; 2843; 1597; 1506 cm^–1^. HRMS (APCI-TOF) *m*/*z*: [M + H]^+^ calcd for C_21_H_22_NO_5_S_2_, 432.0934; found, 432.0934.

##### *N*-(4-Methoxyphenyl)-4-(trifluoromethyl)-*N*-((4-(trifluoromethyl)phenyl)sulfonyl)benzenesulfonamide **3c**

The product was obtained as a whitish powder (0.97 g) in 89% yield. mp 128–131 °C. ^1^H NMR (400 MHz, DMSO-*d*_6_): δ 8.14–8.05 (m, 8H, Ar*H*); 7.02 (br s, 4H, Ar*H*); 3.81 (s, 3H, –OCH_3_) ppm. ^13^C{^1^H} NMR (100 MHz, DMSO-*d*_6_): δ 160.9; 142.1; 134.1 (q, *J* = 32.4 Hz); 132.6; 129.1; 127.0 (q, *J* = 3.7 Hz); 124.9; 123.2 (q, *J* = 271.5 Hz); 115.0; 55.6 ppm. ^19^F NMR (376 MHz, DMSO-*d*_6_): δ −61.81 ppm. IR 3108; 3078; 3057; 2967; 2940; 2922; 2846; 1604; 1509 cm^–1^. HRMS (ESI-TOF) *m*/*z*: [M + H]^+^ calcd for C_21_H_16_F_6_NO_5_S_2_, 540.0369; found, 540.0367.

##### 4-Methoxy-*N*-(4-methoxyphenyl)-*N*-((4-methoxyphenyl)sulfonyl)benzenesulfonamide **3d**

The product was obtained as a white powder (0.72 g) in 76% yield. mp 140–142 °C. ^1^H NMR (400 MHz, DMSO-*d*_6_): δ 7.71 (d, 4H, Ar*H*, *J* = 9.0 Hz); 7.18 (d, 4H, Ar*H*, *J* = 9.0 Hz); 6.95 (d, 2H, Ar*H*, *J* = 9.0 Hz); 6.87 (d, 2H, Ar*H*, *J* = 9.0 Hz); 3.89 (s, 6H, –OCH_3_); 3.78 (s, 3H, –OCH_3_) ppm. ^13^C{^1^H} NMR (100 MHz, DMSO-*d*_6_): δ 163.7; 160.3; 132.5; 130.4; 130.1; 126.0; 114.62; 114.58; 55.9; 55.5 ppm. IR 3102; 3077; 3048; 3005; 2981; 2843; 1592 cm^–1^. HRMS (ESI-TOF) *m*/*z*: [M + H]^+^ calcd for C_21_H_22_NO_7_S_2_, 464.0832; found, 464.0832.

##### *N*,*N*′-(Oxybis(4,1-phenylene))bis(4-nitro-*N*-((4-nitrophenyl)sulfonyl)benzenesulfonamide) **6**

The product was obtained as a beige powder (1.44 g) in 75% yield. mp 167–170 °C. ^1^H NMR (400 MHz, DMSO-*d*_6_): δ 8.52 (d, 8H, Ar*H*, *J* = 9.0 Hz); 8.13 (d, 8H, Ar*H*, *J* = 9.0 Hz); 7.25–7.14 (m, 8H, Ar*H*) ppm. ^13^C{^1^H} NMR (100 MHz, DMSO-*d*_6_): δ 157.6; 151.0; 142.9; 133.5; 129.9; 128.0; 125.1; 120.0 ppm. IR 3109; 3075; 3041; 1608; 1592; 1530 cm^–1^. HRMS (ESI-TOF) *m*/*z*: [M]^+^ calcd for C_36_H_24_N_6_O_17_S_4_, 940.0075; found, 940.0077.

#### Compounds **4**

*N*-(4-Methoxyfenyl)-4-nitrobenzensulfonamide **2Aa** (0.23 g, 0.75 mmol) was dissolved in 30 mL of acetonitrile, and 0.5 g (3.75 mmol) of anhydrous potassium carbonate was added. The mixture was stirred for 0.5 h before the corresponding sulfonyl chloride (0.9 mmol) was added. The resulting mixture was stirred for 3 days. The solvent was removed in vacuo. The residue was dissolved in 50 mL of ethyl acetate and washed with 1 M solution of NaOH (3 × 50 mL). The organic layer was dried over MgSO_4_, filtered, and the filtrate was evaporated to give asymmetrical products.

##### *N*-(4-Methoxyphenyl)-4-methyl-*N*-((4-nitrophenyl)sulfonyl)benzenesulfonamide **4a**

The product was obtained as a whitish powder (0.30 g) in 87% yield. mp 186–188 °C. ^1^H NMR (400 MHz, DMSO-*d*_6_): δ 8.50 (dd, 2H, Ar*H*, *J*_1_ = 9.0 Hz, *J*_2_ = 2.6 Hz); 8.11 (dd, 2H, Ar*H*, *J*_1_ = 9.0 Hz, *J*_2_ = 2.6 Hz); 7.70 (d, 2H, Ar*H*, *J* = 8.4 Hz); 7.51 (d, 2H, Ar*H*, *J* = 8.1 Hz); 7.01–6.94 (m, 4H, Ar*H*); 3.80 (s, 3H, –OCH_3_); 2.46 (s, 3H, –CH_3_) ppm. ^13^C{^1^H} NMR (100 MHz, DMSO-*d*_6_): δ 160.7; 150.7; 145.8; 143.9; 135.2; 132.6; 130.1; 129.6; 128.1; 125.2; 124.9; 114.9; 55.6; 21.2 ppm. IR 3111; 3072; 3034; 2965; 2922; 2845; 1603; 1525; 1502 cm^–1^. HRMS (ESI-TOF) *m*/*z*: [M]^+^ calcd for C_20_H_18_N_2_O_7_S_2_, 462.0550; found, 462.0544.

##### *N*-(4-Methoxyphenyl)-4-nitro-*N*-((4-(trifluoromethyl)phenyl)sulfonyl)benzenesulfonamide **4b**

The product was obtained as a white powder (0.35 g) in 90% yield. mp 178–180 °C. ^1^H NMR (400 MHz, DMSO-*d*_6_): δ 8.51 (dd, 2H, Ar*H*, *J*_1_ = 9.0 Hz, *J*_2_ = 2.6 Hz); 8.18–8.10 (m, 4H, Ar*H*); 8.05 (d, 2H, Ar*H*, *J* = 8.4 Hz); 7.06–6.99 (m, 4H, Ar*H*); 3.81 (s, 3H, –OCH_3_) ppm. ^13^C{^1^H} NMR (100 MHz, DMSO-*d*_6_): δ 161.0; 150.8; 143.3; 142.0; 134.1 (q, *J* = 32.3 Hz); 132.7; 129.8; 129.1; 127.0 (q, *J* = 3.6 Hz); 125.0; 124.8; 123.2 (q, *J* = 271.5 Hz); 115.0; 55.6 ppm. ^19^F NMR (376 MHz, DMSO-*d*_6_): δ −61.81 ppm. IR 3113; 3091; 3018; 2958; 2937; 2913; 2840; 1608; 1540; 1510 cm^–1^. HRMS (APCI-TOF) *m*/*z*: [M + H]^+^ calcd for C_20_H_16_F_3_N_2_O_7_S_2_, 517.0346; found, 517.0347.

##### 4-Methoxy-*N*-(4-methoxyphenyl)-*N*-((4-nitrophenyl)sulfonyl)benzenesulfonamide **4c**

The product was obtained as a white powder (0.31 g) in 86% yield. mp 175–177 °C. ^1^H NMR (400 MHz, DMSO-*d*_6_): δ 8.49 (d, 2H, Ar*H*, *J* = 8.6 Hz); 8.11 (d, 2H, Ar*H*, *J* = 8.6 Hz); 7.72 (d, 2H, Ar*H*, *J* = 8.8 Hz); 7.20 (d, 2H, Ar*H*, *J* = 8.7 Hz); 6.98 (d, 2H, Ar*H*, *J* = 9.0 Hz); 6.95 (d, 2H, Ar*H*, *J* = 9.0 Hz); 3.90 (s, 3H, –OCH_3_), 3.80 (s, 3H, –OCH_3_) ppm. ^13^C{^1^H} NMR (100 MHz, DMSO-*d*_6_): δ 164.0; 160.7; 150.6; 144.1; 132.6; 130.6; 129.6; 129.1; 125.3; 124.9; 114.9; 56.0; 55.6 ppm IR 3101; 3071; 3038; 3000; 2954; 2911; 2846; 1597; 1533; 1500 cm^–1^. HRMS (ESI-TOF) *m*/*z*: [M]^+^ calcd for C_20_H_18_N_2_O_8_S_2_, 478.0499; found, 478.0487.

#### Calix[4]arene **7** (*cone*)

*p*-Aminocalixarene (*cone*) (0.43 g, 0.66 mmol) was dissolved in 40 mL of dichloromethane, and 1.50 g (10 eq., 6.6 mmol) of nosyl chloride and 1 mL (7.2 mmol) of Et_3_N were added. The reaction mixture was refluxed for 7 days and then evaporated to dryness in vacuo. The crude product was suspended in MeOH and filtered to obtain the title compound (770 mg, 55%) as a yellow powder. mp 285–295 °C. ^1^H NMR (400 MHz, CDCl_3_): δ 8.38 (d, 8H, Ar*H*, *J* = 8.9 Hz); 8.04 (d, 8H, Ar*H*, *J* = 8.4 Hz); 7.92 (d, 8H, Ar*H*, *J* = 8.5 Hz); 7.86 (d, 8H, Ar*H*, *J* = 8.9 Hz); 6.63 (s, 8H, Ar*H*); 4.51 (d, 4H, CH_ax_, *J* = 12.9 Hz); 3.99 (t, 8H, –OCH_2_, *J* = 7.8 Hz); 3.08 (d, 4H, CH_eq_, *J* = 13 Hz); 2.08 (sex, 8H, CH_2_, *J* = 7.9 Hz); 1.10 (t, 12H, CH_3_, *J* = 7.5 Hz) ppm. ^13^C{^1^H} NMR (100 MHz, DMSO-*d*_6_): δ 157.8; 150.8; 150.1; 144.4; 141.9; 134.8; 131.0; 129.8; 129.4; 126.7; 124.6; 124.2; 77.0; 29.8; 22.8; 10.1 ppm. IR 3109; 3074; 3040; 2964; 2933; 2875; 1607; 1533 cm^–1^. HRMS (ESI-TOF) *m*/*z*: [M + Na]^+^ calcd for C_88_H_76_N_12_O_36_S_8_Na, 2155.2143; found, 2155.2175.

#### Calix[4]arene **8** (*1*,*3-alternate*)

*p*-Aminocalixarene (*1*,*3-alt*) (1.10 g, 1.68 mmol) was dissolved in 100 mL of dichloromethane, and 3.70 g (10 eq., 16.8 mmol) of nosyl chloride and 2.30 mL (16.5 mmol) of Et_3_N and were added. The reaction was refluxed for 14 days. After evaporation of all the solvent, the residue was triturated with a small amount of methanol. The title compound was obtained by filtration as a yellow solid (2.0 g) in 56% yield. mp > 300 °C. ^1^H NMR (400 MHz, DMSO-*d*_6_): δ 8.46 (d, 16H, Ar*H*, *J* = 8.9 Hz); 8.24 (d, 8H, Ar*H*, *J* = 8.8 Hz); 7.82 (d, 8H, Ar*H*, *J* = 8.8 Hz); 6.89 (s, 8H, Ar*H*); 3.85 (s, 8H, ArCH_2_Ar); 3.26 (t, 8H, –OCH_2_, *J* = 8.2 Hz); 1.0 (sex, 8H, CH_2_, *J* = 8.2 Hz); 0.45 (t, 12H, CH_3_, *J* = 7.6 Hz) ppm. ^13^C{^1^H} NMR (100 MHz, DMSO-*d*_6_): δ 159.2; 150.9; 150.6; 145.1; 141.9; 134.0; 130.8; 130.0; 125.6; 124.8; 124.7; 72.1; 36.3; 21.6; 9.8 ppm. IR 3107; 3069; 3038; 2967; 2936; 2875; 1606; 1528 cm^–1^. HRMS (ESI-TOF) *m*/*z*: [M + Na]^+^ calcd for C_88_H_76_N_12_O_36_S_8_Na, 2155.2143; found, 2155.2190.

#### Calix[4]arene **9** (*1*,*2-alternate*)

*p*-Aminocalixarene (*1,2-alt*) (66 mg, 0.085 mmol) was dissolved in 10 mL of dichloromethane, and 0.38 g of nosyl chloride (20 equiv, 1.7 mmol) and 0.10 mL (0.71 mmol) of Et_3_N were added. The reaction mixture was refluxed for 7 days. The product was precipitated by the addition of a large amount of methanol (50 mL). Filtration of the precipitate yielded 106 mg of the product as a yellow solid (58%). mp 208–222 °C. ^1^H NMR (400 MHz, CDCl_3_): δ 8.39 (dd, 16H, Ar*H*, *J* = 8.6 Hz); 8.26 (d, 8H, Ar*H*, *J* = 8.9 Hz); 8.00 (d, 8H, Ar*H*, *J* = 8.9 Hz); 6.97 (d, 4H, Ar*H*, *J* = 2.7 Hz); 6.77 (d, 4H, Ar*H*, *J* = 2.7 Hz); 4.34 (d, 2H, CH_ax_, *J* = 12.8 Hz); 3.8 (s, 4H, ArCH_2_Ar); 3.51–3.43 (m, 4H, –OCH_2_); 3.41–3.34 (m, 4H, –OCH_2_); 3.09 (d, 2H, CH_eq_, *J* = 12.9 Hz); 1.43–1.32 (m, 4H, CH_2_); 1.14–1.03 (m, 4H, CH_2_); 0.64 (t, 12H, CH_3_, *J* = 7.3 Hz) ppm. ^13^C{^1^H} NMR (100 MHz, DMSO-*d*_6_): δ 158.4; 150.9; 150.7; 145.0; 142.0; 135.1; 133.7; 131.6; 130.9; 130.0; 129.5; 126.3; 124.8; 124.7; 77.6; 35.9; 28.3; 21.5; 9.8 ppm. IR 3108; 3074; 3039; 2963; 2935; 2874; 1607; 1533 cm^–1^. HRMS (ESI-TOF) *m*/*z*: [M + Na]^+^ calcd for C_88_H_76_N_12_O_36_S_8_Na, 2155.2143; found, 2155.2139.

### Electrochemistry

Electrochemical experiments (DC-polarography and cyclic voltammetry) were performed in DMSO (for DNA and peptide synthesis, Merck, containing max 0.025% H_2_O) using 0.1 M tetrabutylammonium hexafluorophosphate (>98.0%, TCI) as the supporting electrolyte at room temperature. In the case of experiments at low temperature (−44 °C), DMSO was exchanged for DMF (extra dry, H_2_O < 50 ppm, 99.8%, dried over molecular sieves, Lachner). Due to low conductivity, the three-electrode system was applied. As the working electrode, a dropping mercury electrode (drop time 1 s, scan rate 10 mV·s^–1^), hanging mercury drop electrode, glassy carbon (GC) electrode (diameter 3 mm), rotating disc glassy carbon electrode (RDE, diameter 1 mm), or Pt disk electrode (1 mm) were used. Scan rates used for cyclic voltammetry experiments: 100, 200, and 500 mV·s^–1^. Linear sweep voltammetry on RDE was measured at a scan rate 10 mV·s^–1^ with several rotation rates (100, 250, 500, 1000, and 2000 s^–1^). Before each measurement, the solid electrodes (Pt, GC) were mechanically cleaned using a polishing pad. As the reference electrode, a saturated calomel electrode (SCE) separated from the investigated sample by a salt-bridge filled by the blank (DMSO electrolyte solution) was used, and as the counter (auxiliary) electrode, Pt sheet was applied. All experiments were carried out in an undivided 20 mL cell. Oxygen was removed from the solution by passing a stream of argon (Ar, 99.998%, Messer). The polarographic experiments were conducted by analogue polarographic analyzer PA4 with XY recorder, both Laboratorní přístroje Praha (CZ). Cyclic voltammetry measurements were carried out using the computer-driven digital potentiostat PGSTAT101 (Autolab-Metrohm) controlled by software NOVA 1.11. The concentration of studied compounds depended on the number of sulfonyl groups and is for each compound specified in ESI in the descriptions.

### Electrochemistry under Preparative Conditions (Bulk Electrolysis)

Sulfonimides were cleaved by the controlled potential electrolysis. For this purpose, a standard electrochemical divided H-type cell, where anodic and cathodic compartments (each max volume 10 mL) are separated by dense frit, was applied. As working electrodes, Pt sheet (5 × 30 mm) and a mercury pool stirred during electrolysis by a magnetic bar, or glassy carbon sheet (5 × 30 mm) were used. The silver/silver chloride reference electrode and counter Pt wire, or mesh were utilized. The electrolysis was performed in DMSO (or DMS0-*d*_6_). Both compartments contain 60 mM solution of KPF_6_ (supporting electrolyte). In addition, the cathodic compartment contained 10 mM concentration of corresponding monoimides (in the case of bis- or tetrakis-imides **6–8**, the used concentration was 5 mM, and for calixarene **9**, 2.5 mM concentration) in 4 mL of the supporting electrolyte. In the cases when the splitting potential approached −2.0 V, the TBA-based supporting electrolyte was utilized instead of KPF_6_. During data collection, both compartments were deaerated by Ar. Measurements were carried out using the computer-driven digital potentiostat PGSTAT101 (Autolab-Metrohm) controlled by software NOVA 1.11 or 2.1. The applied potential was at least set up 100 mV behind the splitting potential (the first reduction step) and before the next reduction step obtained by cyclic voltammetry. The electrolysis proceeds until the current value has fallen below 90% of its initial value. Then, the crude mixture was collected, frozen by immersing the vial in −78 °C bath and lyophilized to dryness. The mixture was dissolved into ethyl-acetate and washed with an aqueous solution of HCl (3 × 50 mL). The organic layer was dried over MgSO_4_, filtered, and evaporated. The final products were separated by using a preparative TLC plate (EA/PE 1:1) and characterized.

### Batch Electrolysis Setup

A 20 mL undivided cell with working and counter (auxiliary) electrodes directly placed into the same compartment, equipped with mercury pool (working electrode), and the silver/silver chloride reference electrode were used. As a counter electrode, two different materials were tested—Pt sheet (5 × 30 mm) or magnesium bar. The electrolysis was performed in DMSO containing KPF_6_ as the supporting electrolyte (60 mM in 10 mL) and sulfonimide **2a** (ca. 20 mg). During electrolysis, the cell was deaerated by Ar, and solution was stirred by a magnetic bar. Measurements were carried out using the computer-driven digital potentiostat PGSTAT101 (Autolab-Metrohm) controlled by software NOVA 1.11. The applied potential was set up at −0.9 V, and electrolysis continued until the current value has fallen below 90% of the initial value. Then, the mixture was treated in the same way as described above.

### Compound **2Aa**

Product **2Aa** was obtained by cleavage of **2a** (19.7 mg, 39.9 μmol) as an orange powder (10.5 mg) in 85% yield. The spectral characterization is identical to that previously reported.^43^

### Compound **2Ab**

The product **2Ab** was obtained by cleavage of **2b** (20.9 mg, 45.1 μmol) as a yellow powder (10.9 mg) in 87% yield. ^1^H NMR (400 MHz, DMSO-*d*_6_): δ 10.59 (s, 1H, N*H*); 8.37 (dd, 2H, ArH, *J*_1_ = 8.9 Hz, *J*_2_ = 2.5 Hz); 7.98 (dd, 2H, Ar*H*, *J*_1_ = 9.0 Hz, *J*_2_ = 2.4 Hz); 7.26 (dd, 2H, Ar*H*, *J*_1_ = 8.4 Hz, *J*_2_ = 4.2 Hz); 7.11–7.06 (m, 3H, Ar*H*) ppm. The spectral characterization is identical to that previously reported.^40^

### Compound **2Ac**

The product **2Ac** was obtained by cleavage of **2c** (21.0 mg, 39.5 μmol) as a yellowish powder (12.0 mg) in 88% yield. ^1^H NMR (400 MHz, DMSO-*d*_6_): δ 11.19 (br s, 1H, N*H*); 8.39 (d, 2H, Ar*H*, *J* = 8.9 Hz); 8.06 (d, 2H, Ar*H*, *J* = 8.9 Hz); 7.63 (d, 2H, Ar*H*, *J* = 8.6 Hz); 7.31 (d, 2H, Ar*H*, *J* = 8.5 Hz) ppm. The spectral characterization is identical to that previously reported.^40^

### Compound **3Aa**

The product **3Aa** was obtained by cleavage of **3a** (21.8 mg, 44.2 μmol) as a yellowish powder (11.6 mg) in 87% yield. ^1^H NMR (400 MHz, DMSO-*d*_6_): δ 10.33 (s, 1H, N*H*); 7.99 (d, 1H, Ar*H*, *J* = 3,4 Hz); 7.88–7.76 (m, 3H, Ar*H*) 7.04 (d, 2H, Ar*H*, *J* = 8.8 Hz); 6.84 (d, 2H, Ar*H*, *J* = 8.8 Hz); 3.68 (s, 3H, –OCH_3_) ppm.

### Compound **4Aa** = **3Ab**

The product **3Ab** was obtained by cleavage of **3b** (17.2 mg, 39.9 μmol) as a whitish powder in (9.4 mg) 85% yield, or **4a** (18.5 mg, 40.0 μmol) in 87% (9.7 mg) yield. ^1^H NMR (400 MHz, DMSO-*d*_6_): δ 9.82 (s, 1H, N*H*); 7.55 (d, 2H, Ar*H*, *J* = 8.2 Hz); 7.32 (d, 2H, Ar*H*, *J* = 8.0); 6.96 (d, 2H, Ar*H*, *J* = 9.0 Hz); 6.78 (d, 2H, Ar*H*, *J* = 9.0 Hz); 3.66 (s, 3H, –OCH_3_); 2.33 (s, 3H, CH_3_) ppm. The spectral characterization is identical to that previously reported.^43^

### Compound **4Ab** = **3Ac**

The product **3Ac** was obtained by cleavage of **3c** (21.6 mg, 40.0 μmol) as a whitish powder (10.6 mg) in 80% yield, or **4b** (21.8 mg, 42.2 μmol) in 78% (10.9 mg) yield. ^1^H NMR (400 MHz, DMSO-*d*_6_): δ 10.14 (s, 1H, N*H*); 7.94 (d, 2H, Ar*H*, *J* = 8.4 Hz); 7.87 (d, 2H, Ar*H*, *J* = 8.3 Hz); 6.97 (d, 2H, Ar*H*, *J* = 9.0 Hz); 6.81 (d, 2H, Ar*H*, *J* = 9.0 Hz); 3.67 (s, 3H, –OCH_3_) ppm. The spectral characterization is identical to that previously reported.^44^

### Compound **4Ac** = **3Ad**

The product **3Ad** (**4Ac** respectively) was obtained by cleavage of **3d** (18.5 mg, 39.9 μmol) as an orange powder (9.4 mg) in 80% yield, or **4c** (20.0 mg, 41.8 μmol) in 89% (10.9 mg) yield. ^1^H NMR (400 MHz, DMSO-*d*_6_): δ 9.74 (s, 1H, N*H*); 7.60 (d, 2H, Ar*H*, *J* = 8.8 Hz); 7.03 (d, 2H, Ar*H*, *J* = 8.9 Hz); 6.96 (d, 2H, Ar*H*, *J* = 8.9 Hz); 6.79 (d, 2H, Ar*H*, *J* = 8.9 Hz); 3.79 (s, 3H, –OCH_3_) 3.66 (s, 3H, –OCH_3_) ppm. The spectral characterization is identical to that previously reported.^45^

### Compound **6A**

The product **6A** was obtained by cleavage of compound **6** (19.1 mg, 20.3 μmol) as a yellowish powder (9.4 mg) in 81% yield. mp 67–70 °C. ^1^H NMR (400 MHz, DMSO-*d*_6_): δ 10.48 (s, 2H, N*H*); 8.37 (d, 4H, Ar*H*, *J* = 8.9 Hz); 7.94 (d, 4H, Ar*H*, *J* = 8.9 Hz); 7.04 (d, 4H, Ar*H*, *J* = 8.9 Hz); 6.84 (d, 4H, Ar*H*, *J* = 9.0 Hz) ppm. ^13^C{^1^H} NMR (100 MHz, DMSO-*d*_6_): δ 153.9; 149.9; 145.0; 132.3; 128.4; 124.7; 123.6; 119.4 ppm. IR 3261; 3105; 3068; 3038; 2957; 2924; 2852; 1696; 1606; 1527 cm^–1^. HRMS (ESI-TOF) *m*/*z*: [M]^+^ calcd for C_24_H_18_N_4_O_9_S_2_, 570.0510; found, 570.0512; [M + Na]^+^ calcd for C_24_H_18_N_4_O_9_S_2_Na, 593.0407; found, 593.0414.

### Sulfonamide **7A**

The product **7A** was obtained by cleavage of compound **7** (41.1 mg, 19.3 μmol) as a yellow powder (21.5 mg) in 80% yield. mp 137–142 °C. ^1^H NMR (400 MHz, DMSO-*d*_6_): δ 9.88 (br s, 4H, N*H*); 8.34 (d, 8H, Ar*H*, *J* = 8.7 Hz); 7.86 (d, 8H, Ar*H*, *J* = 8.7 Hz); 6.34 (s, 8H, Ar*H*); 4.12 (d, 4H, CH_ax_, *J* = 13.2 Hz); 3.64 (t, 8H, –OCH_2_, *J* = 7.2 Hz); 2.92 (d, 4H, CH_eq_, *J* = 13.3 Hz); 1.73 (m, 8H, CH_2_); 0.86 (t, 12H, CH_3_, *J* = 7.4 Hz) ppm. ^13^C{^1^H} NMR (100 MHz, DMSO-*d*_6_): δ 153.5; 149.6; 145.4; 134.7; 130.5; 128.1; 124.4; 121.7; 76.3; 30.3; 22.6; 10.1 ppm. IR 3268; 3105; 2962; 2931; 2874; 1605; 1589; 1530 cm^–1^. HRMS (ESI-TOF) *m*/*z*: [M + H]^+^ calcd for C_64_H_65_N_8_O_20_S_4_, 1393.3193; found, 1393.3187; [M + Na]^+^ calcd for C_64_H_64_N_8_O_2_0S_4_Na, 1415.3012; found, 1415.3019.

### Sulfonamide **8A**

The product was obtained by cleavage of compound **8** (41.3 mg, 19.4 μmol) as a yellow powder (18.9 mg) in 70% yield. mp 145–149 °C. ^1^H NMR (400 MHz, DMSO-*d*_6_): δ 10.18 (s, 4H, N*H*); 8.36 (d, 8H, Ar*H*, *J* = 8.8 Hz); 7.96 (d, 8H, Ar*H*, *J* = 8.8 Hz); 6,71 (s, 8H, Ar*H*); 3.47 (s, 8H, ArCH_2_Ar); 2.99 (t, 8H, –OCH_2_, *J* = 7.3 Hz); 1.00 (sex, 8H, CH_2_, *J* = 7.4 Hz); 0.52 (t, 12H, CH_3_, *J* = 7.3 Hz) ppm. ^13^C{^1^H} NMR (100 MHz, DMSO-*d*_6_): δ 153.6; 149.6; 145.8; 134.0; 130.4; 128.2; 124.5; 121.8; 71.8; 37.3; 22.0; 9.8 ppm. IR 3234; 3105; 3038; 2967; 2937; 2873; 1602; 1529 cm^–1^. HRMS (ESI-TOF) *m*/*z*: [M + H]^+^ calcd for C_64_H_65_N_8_O_20_S_4_, 1393.3193; found, 1393.3170; [M + Na]^+^ calcd for C_64_H_64_N_8_O_2_0S_4_Na, 1415.3012; found, 1415.2992.

### Sulfonamide **9A**

The product was obtained by cleavage of compound **8** (21.8 mg, 10.2 μmol) as a yellow powder (10.8 mg) in 76% yield. mp 99–105 °C. ^1^H NMR (400 MHz, DMSO-*d*_6_): δ 10.30 (s, 4H, N*H*); 8.39 (d, 8H, Ar*H*, *J* = 8.8 Hz); 8.05 (d, 8H, Ar*H*, *J* = 8.8 Hz); 6.94 (d, 4H, Ar*H*, *J* = 2.2 Hz); 6.62 (d, 4H, Ar*H*, *J* = 2.0 Hz); 3.87 (d, 2H, CH_ax_, *J* = 12.5 Hz); 3.47 (s, 4H, ArCH_2_Ar); 3.09–2.98 (m, 8H, 2x –OCH_2_); 2.92 (d, 2H, CH_eq_, *J* = 12.9 Hz); 0.92–0.84 (m, 4H, CH_2_); 0.74–0.65 (m, 4H, CH_2_); 0.37 (t, 12H, CH_3_, *J* = 7.4 Hz) ppm. ^13^C{^1^H} NMR (100 MHz, DMSO-*d*_6_): δ 153.3; 149.7; 146.6; 134.1; 132.7; 130.7; 128.4; 127.0; 124.5; 123.4; 121.7; 121.4; 74.3; 36.9; 29.1; 28.6; 22.0; 21.1; 9.93 ppm. IR 3357; 3104; 3072; 3039; 2961; 2929; 2872; 2858; 1715; 1649; 1603; 1527 cm^–1^. HRMS (ESI-TOF) *m*/*z*: [M]^+^ calcd for C_64_H_64_N_8_O_20_S_4_, 1392.3119; found, 1392.3061; [M + Na]^+^ calcd for C_64_H_64_N_8_O_20_S_4_Na, 1415.3012; found, 1415.3010.

## Calculations

The DFT geometry optimizations have been performed in Gaussian 16W^46^ using B3LYP functional with 6-31** basis. To visualize molecular orbitals and charge distribution, the GaussView 6.0 was used.

## Data Availability

The data underlying this study are available in the published article and its online Supporting Information.

## Supporting Information Available

Copies of^1^H NMR,^13^C NMR, IR, and HRMS spectra of all newly synthesized compounds; electrochemical data (cyclic voltammetry, DC polarography, and RDE measurements); and results of DFT calculations (PDF)
