# Supplementary material for: Chemoselective Electrochemical Cleavage of Sulfonimides as a Direct Way to Sulfonamides
Source: J Org Chem. 2024 Jan 10;89(3):1425–37. doi: 10.1021/acs.joc.3c01932 (PMC10845148; doi:10.1021/acs.joc.3c01932)
Supplement: Supplementary file 1 — jo3c01932_si_001.pdf [file jo3c01932_si_001.pdf]

Supporting information

Chemoselective Electrochemical Cleavage of Sulfonimides As a Direct Way to Sulfonamides

Karolína Salvadori,[a,b,c*] Michal Churý,[d] Jan Budka,[d] Jakub Harvalík,[b] Pavel Matějka,[b] Ludmila Šimková,[a] and Pavel Lhoták[d*]

[a.] *J. Heyrovský Institute of Physical Chemistry of Czech Academy of Sciences v.v.i., Dolejškova 2155/3, 182 23 Prague 8, Czech Republic.*
[b.] *Department of Physical Chemistry, University of Chemistry and Technology, Prague (UCTP), Technická 5, 166 28 Prague 6, Czech Republic.*
[c.] *Institute of Chemical Process Fundamentals of Czech Academy of Sciences v.v.i., Rozvojová 135, 165 02 Prague 6, Czech Republic.*
[d.] *Department of Organic Chemistry, UCTP, Technická 5, 166 28 Prague 6, Czech Republic.*

Corresponding Author
* E-mail: salvadok@vscht.cz
* E-mail: pavel.lhotak@vscht.cz

Content

1. Spectral characterisation of studied sulfonimides S2
2. Spectral characterisation of obtained sulfonamides S36
3. DFT calculations S49
4. Electrochemical study S52
   4.1 Summary S52
   4.2 Detail description of derivative 2a S53
      4.2.1 Polarography and cyclic voltammetry S53
      4.2.2 Electrolysis S55
      4.2.3 Further electrochemical investigation S58
   4.3 Cyclic voltammograms S60
   4.4 Mixture containing sulfonimide with sulfonamide S68

1. Spectral characterisation of studied sulfonimides

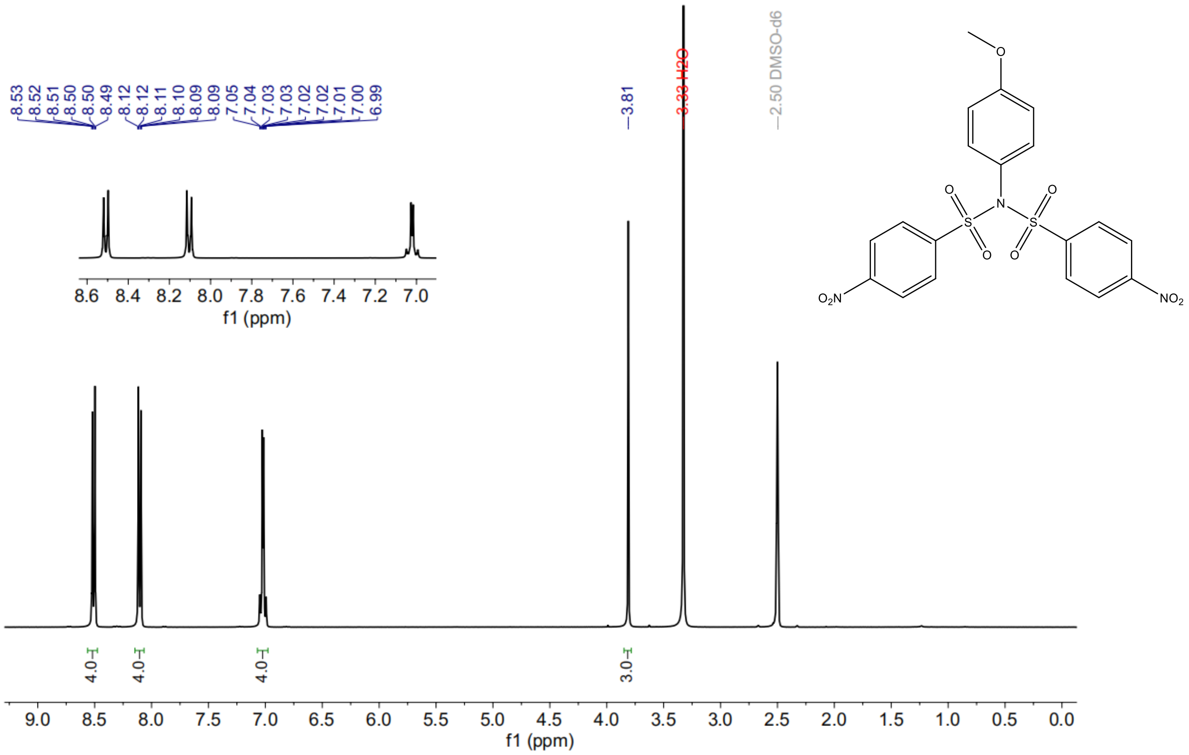

Fig. S1: ^{1}H NMR of compound **2a** (DMSO-d_6, 400 MHz).

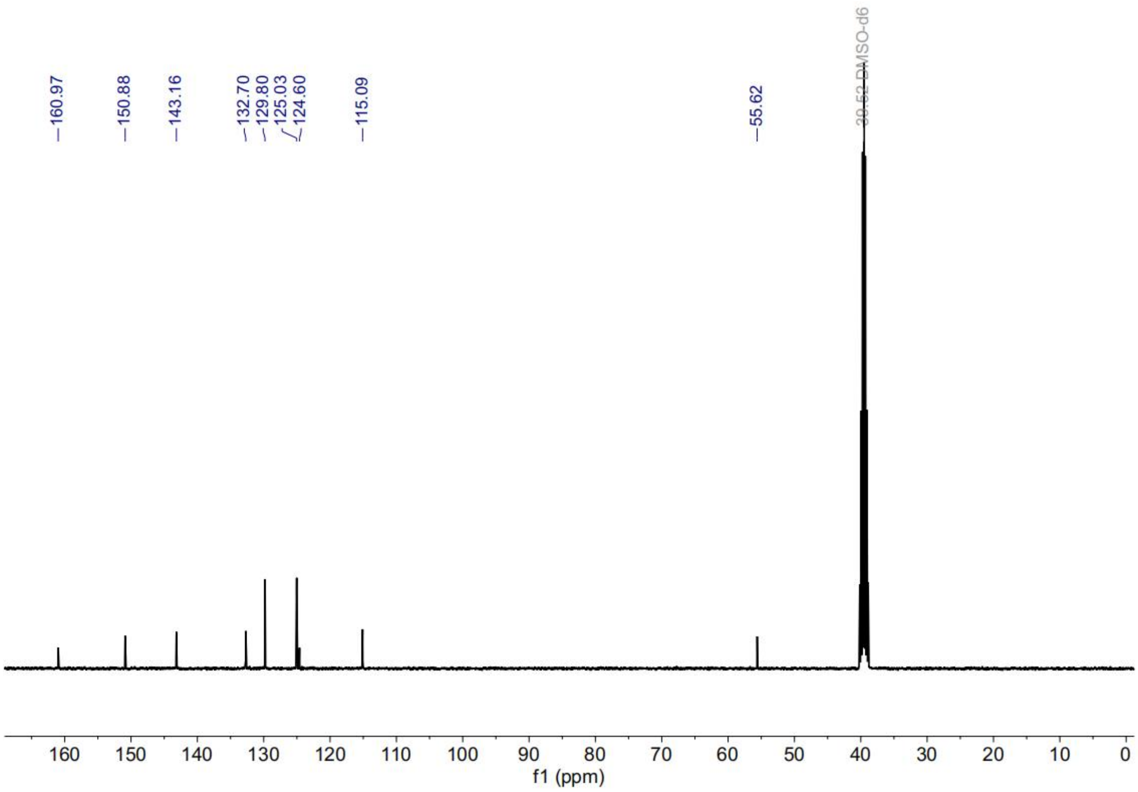

Fig. S2: ^{13}C{^{1}H} NMR of compound **2a** (DMSO-d_6, 100 MHz).

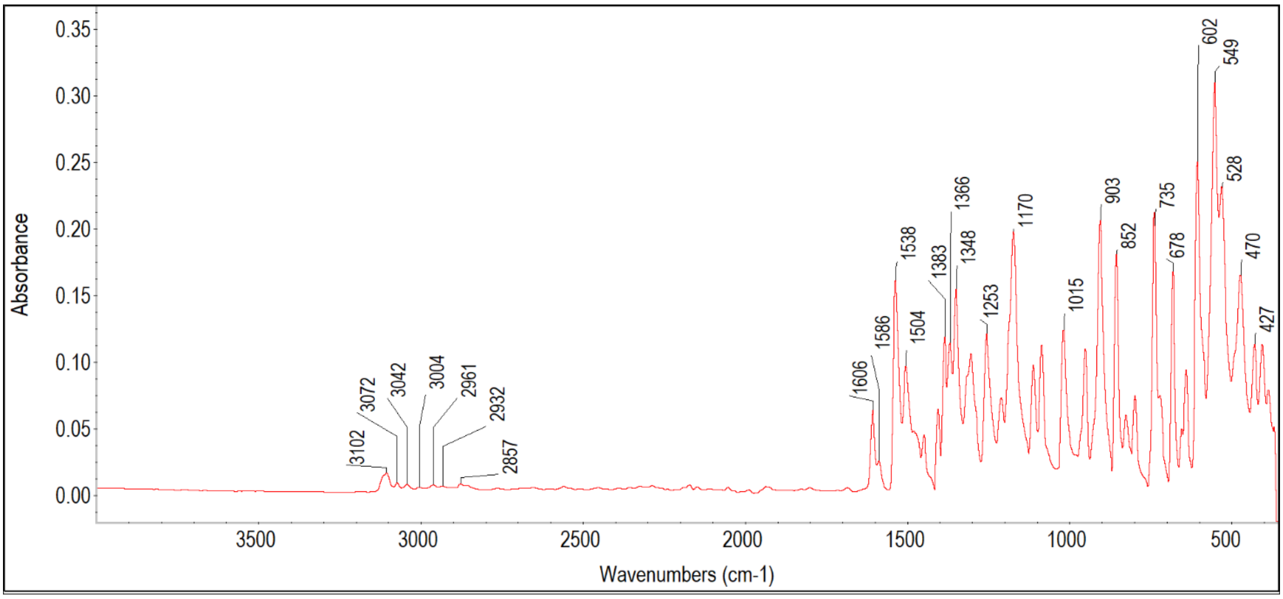

Fig. S3: IR spectrum of compound **2a** (ATR).

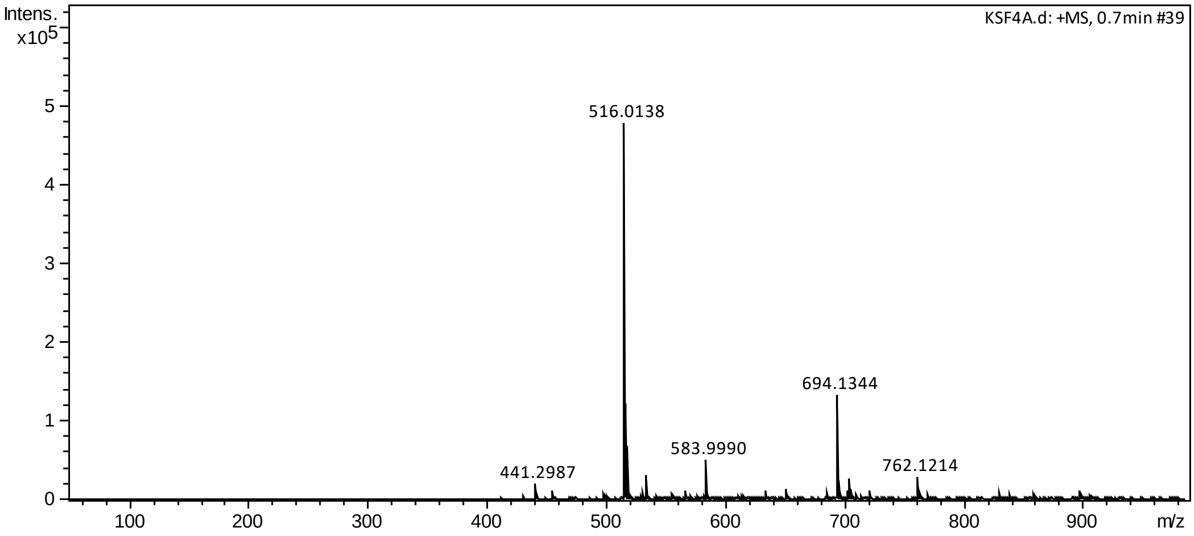

Fig. S4: HRMS of compound **2a** calc $[C_{19}H_{15}N_3O_9S_2+Na]^+$ 516.0141; found *m/z* 516.0138 $[M+Na]^+$.

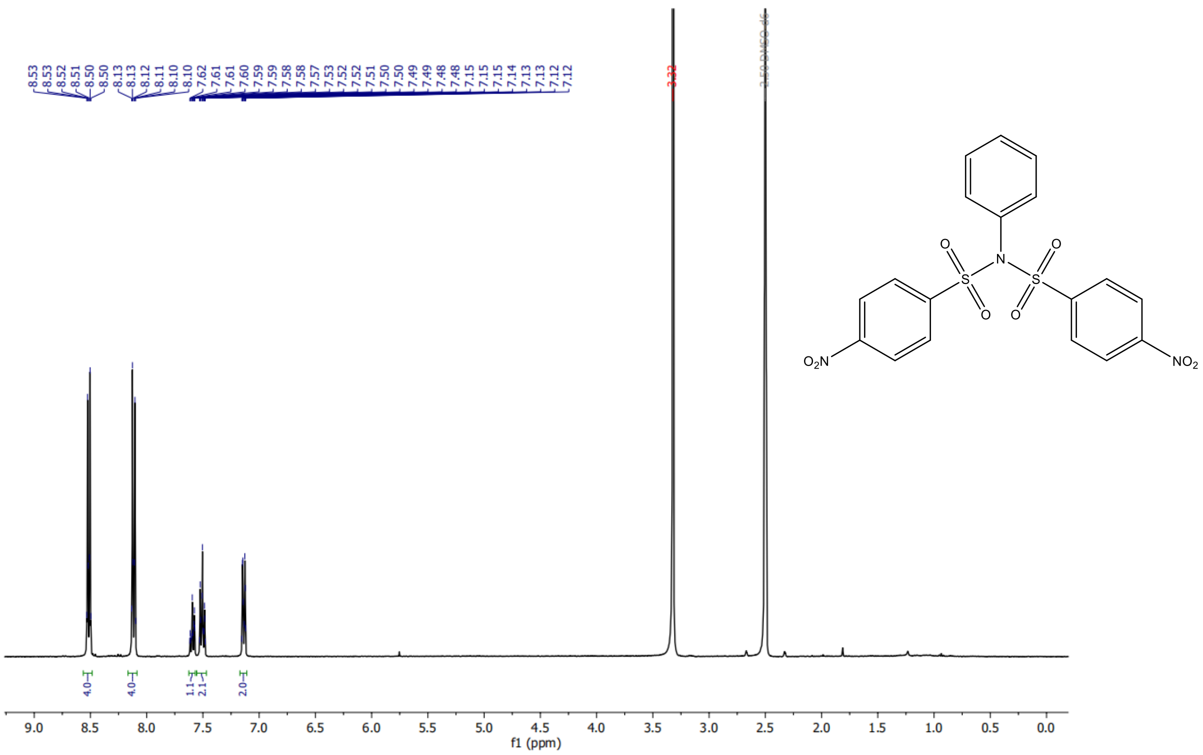

Fig. S5: ^{1}H NMR of compound **2b** (DMSO-d_6, 400 MHz).

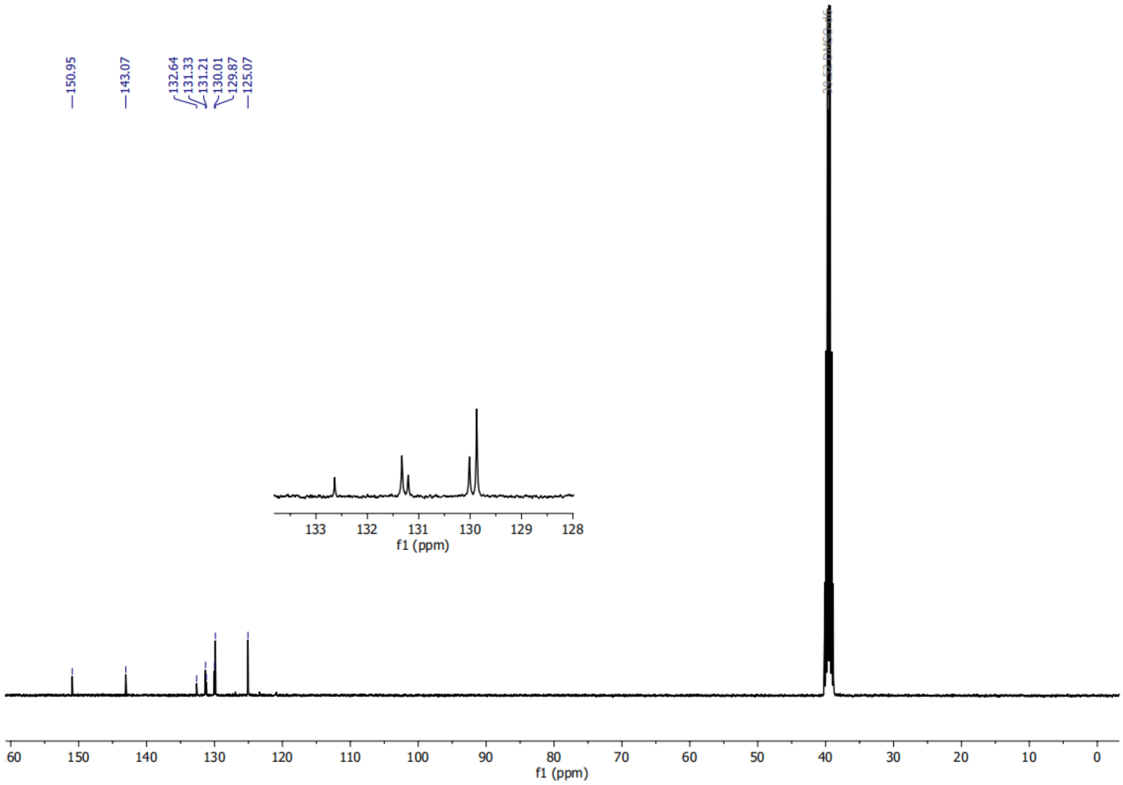

Fig. S6: ^{13}C{^{1}H} NMR of compound **2b** (DMSO-d_6, 100 MHz).

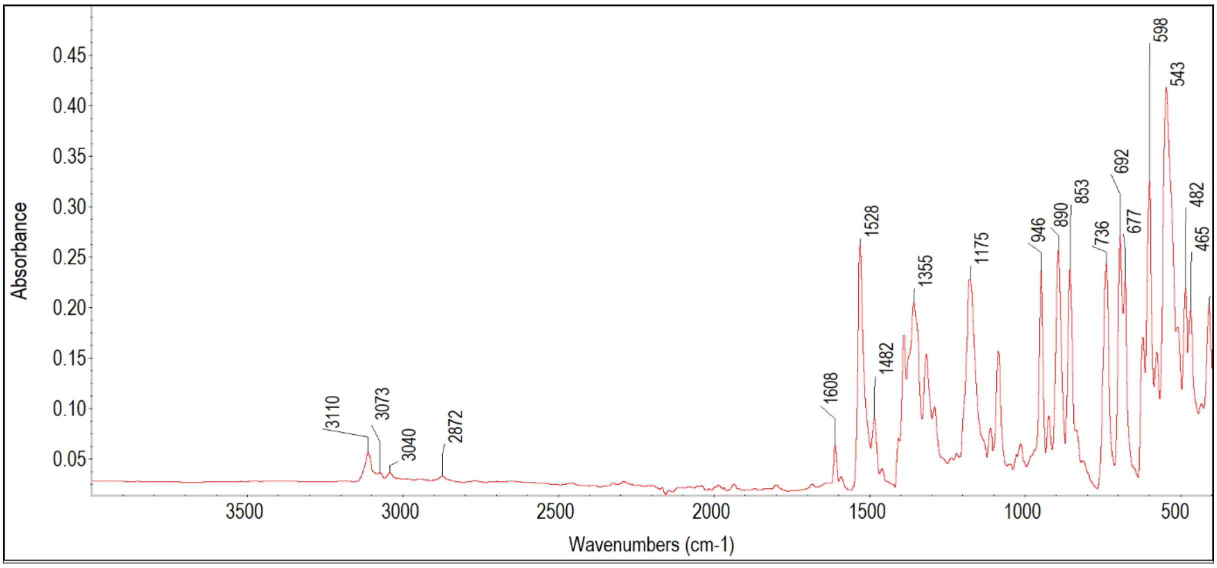

Fig. S7: IR spectrum of compound **2b** (ATR).

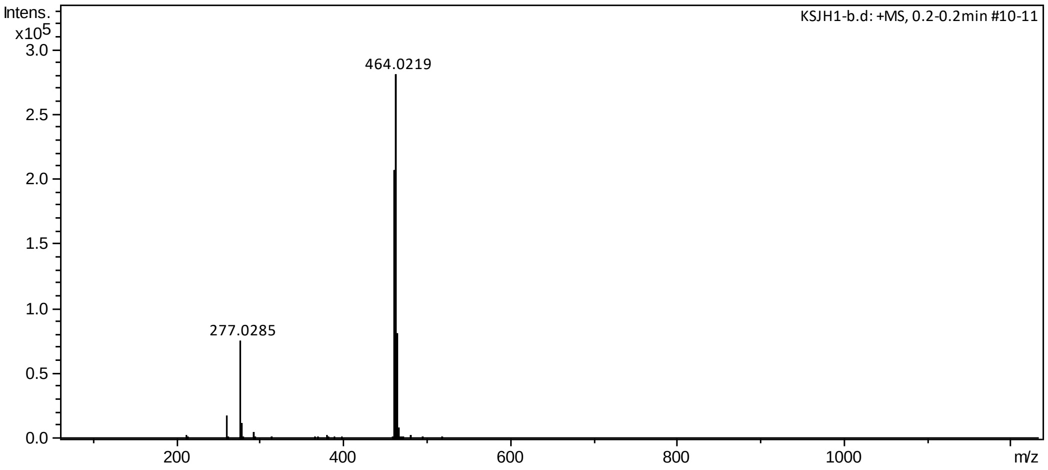

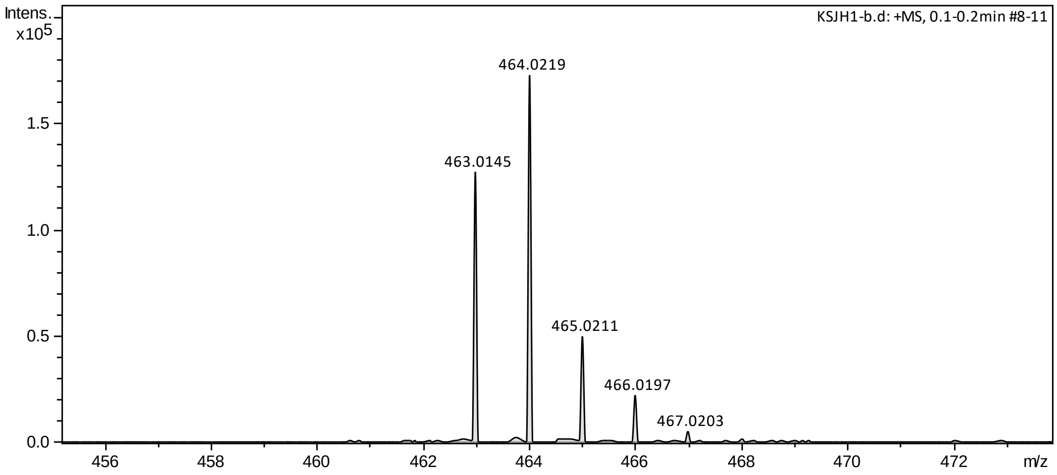

Fig. S8: HRMS of compound **2b** calc $[C_{18}H_{14}N_3O_8S_2]^+$ 464.0217; found *m/z* 464.0219 $[M+H]^+$.

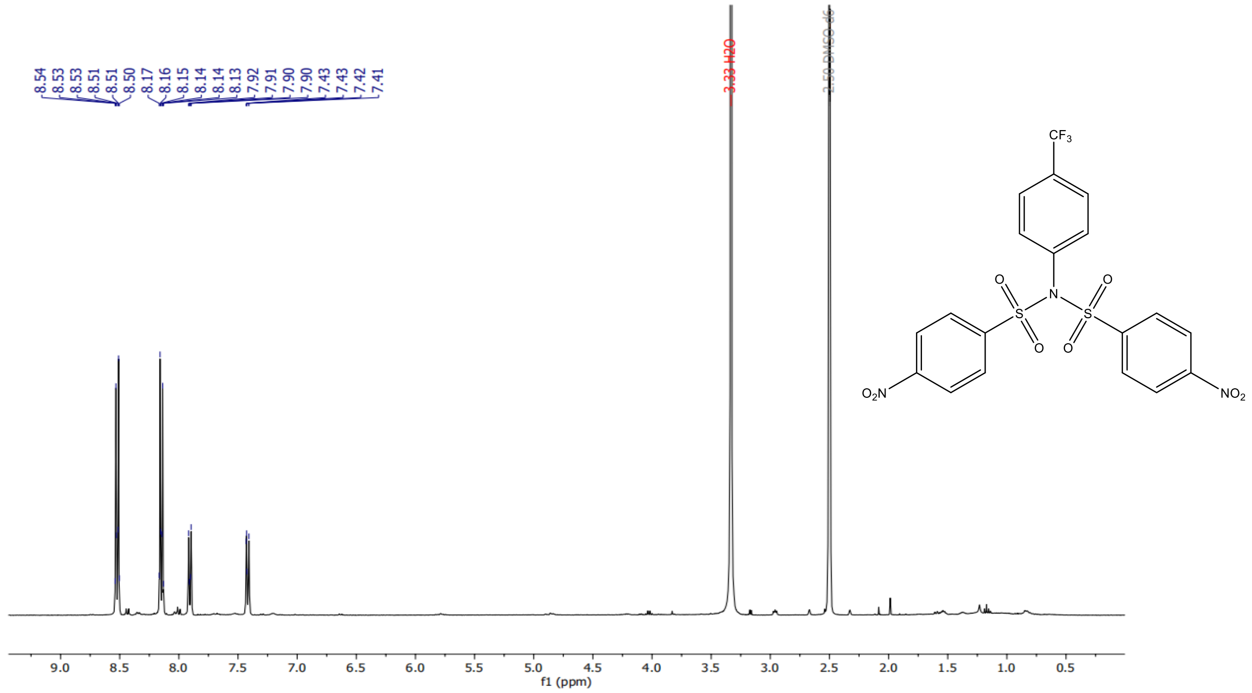

Fig. S9: ^{1}H NMR of compound **2c** (DMSO-d_6, 400 MHz).

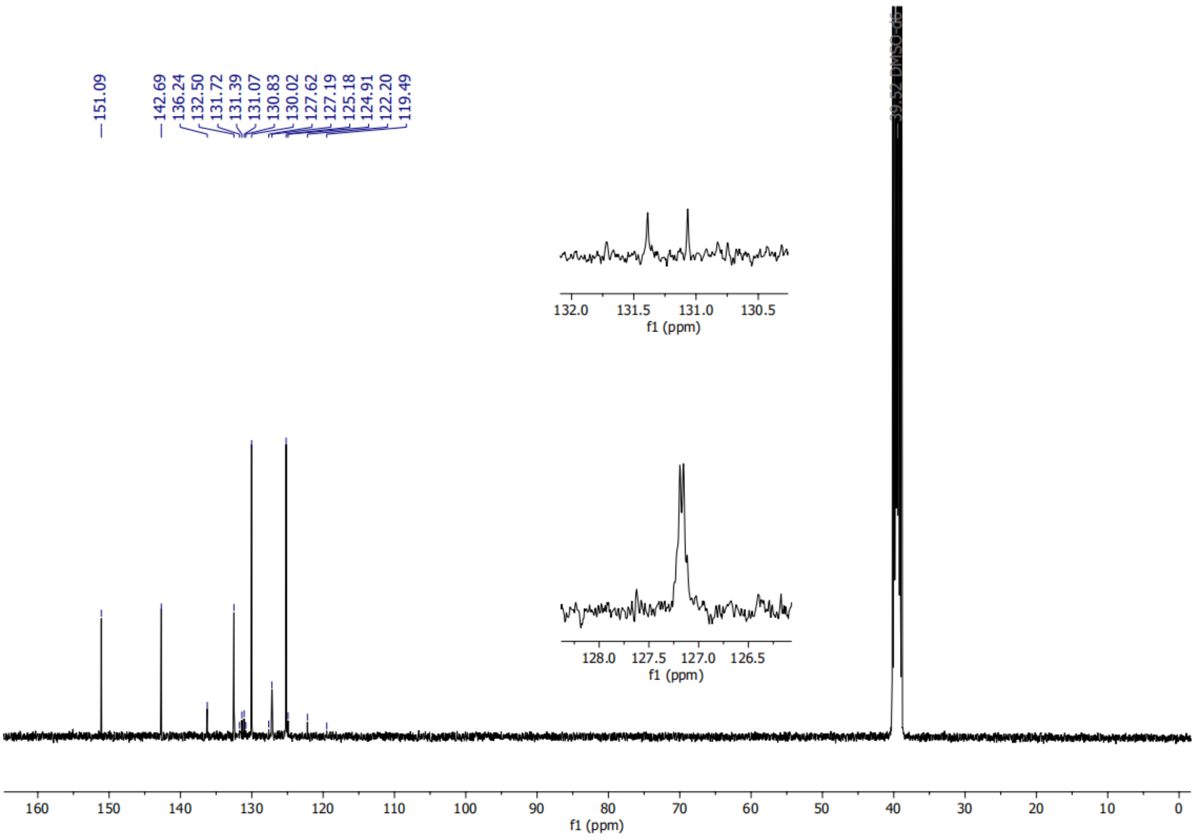

Fig. S10: ^{13}C{^{1}H} NMR of compound **2c** (DMSO-d_6, 100 MHz).

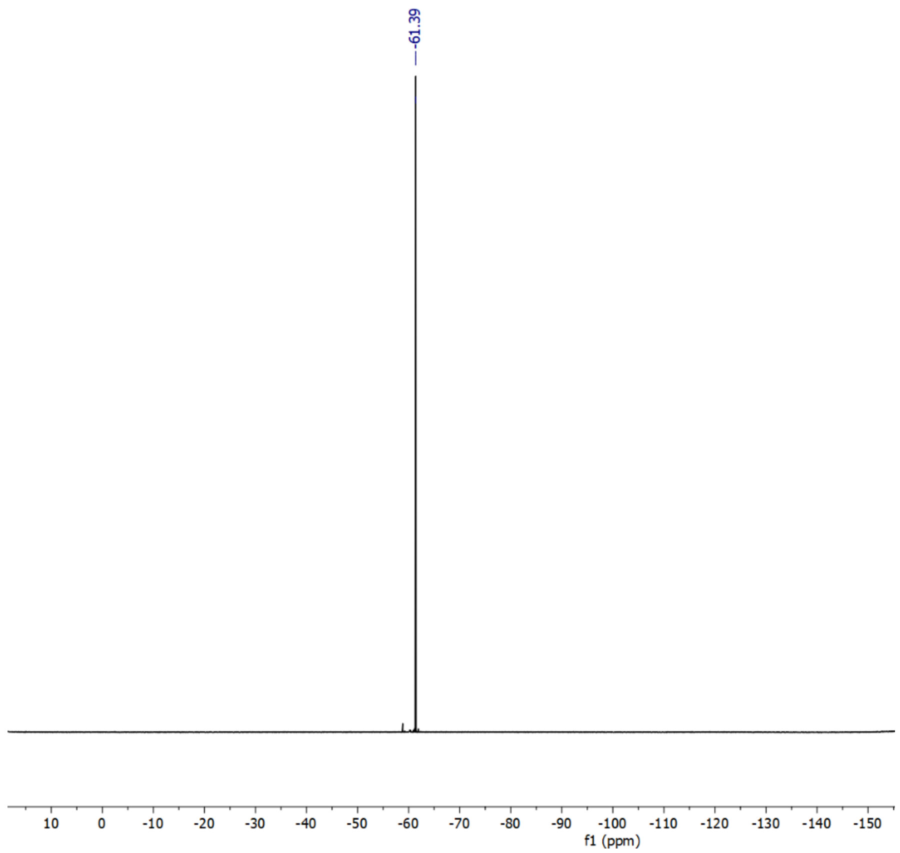

Fig. S11: ^{19}F NMR of compound **2c** (DMSO-d_6, 376 MHz).

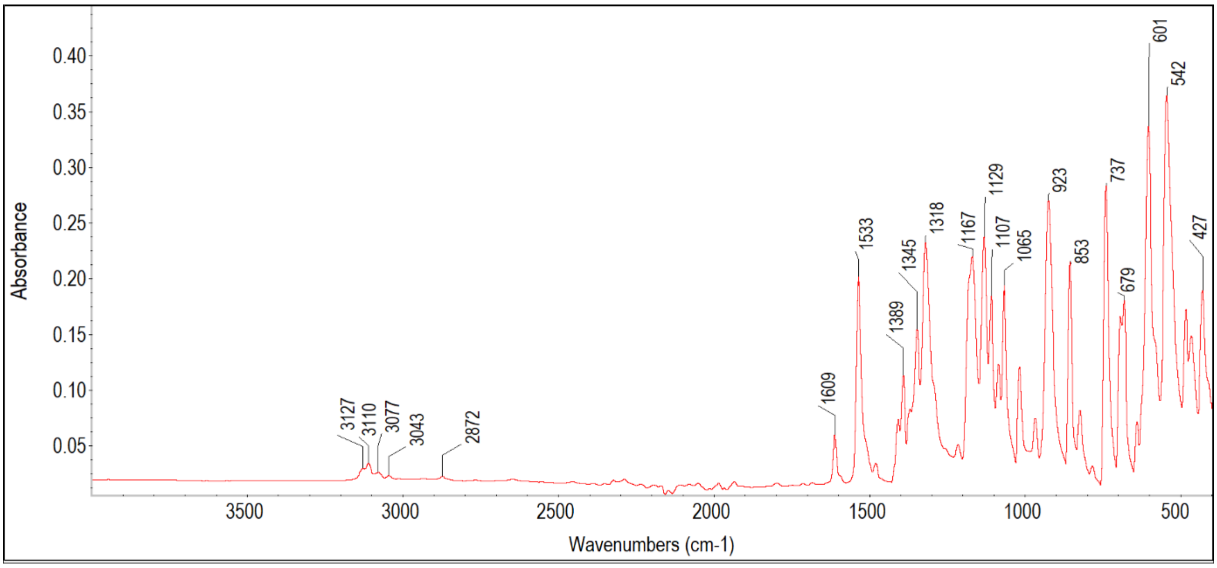

Fig. S12: IR spectrum of compound **2c** (ATR).

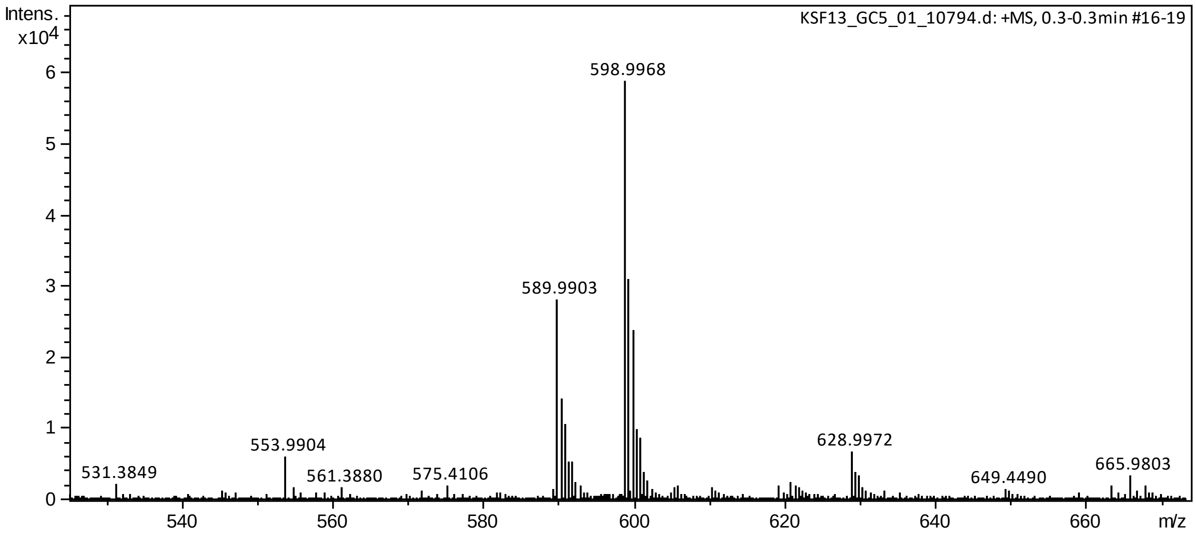

Fig. S13: HRMS of compound **2c** calc $[C_{19}H_{12}F_3N_3O_8S_2+Na]^+$ 553.9910; found *m/z* 553.9904 $[M+Na]^+$.

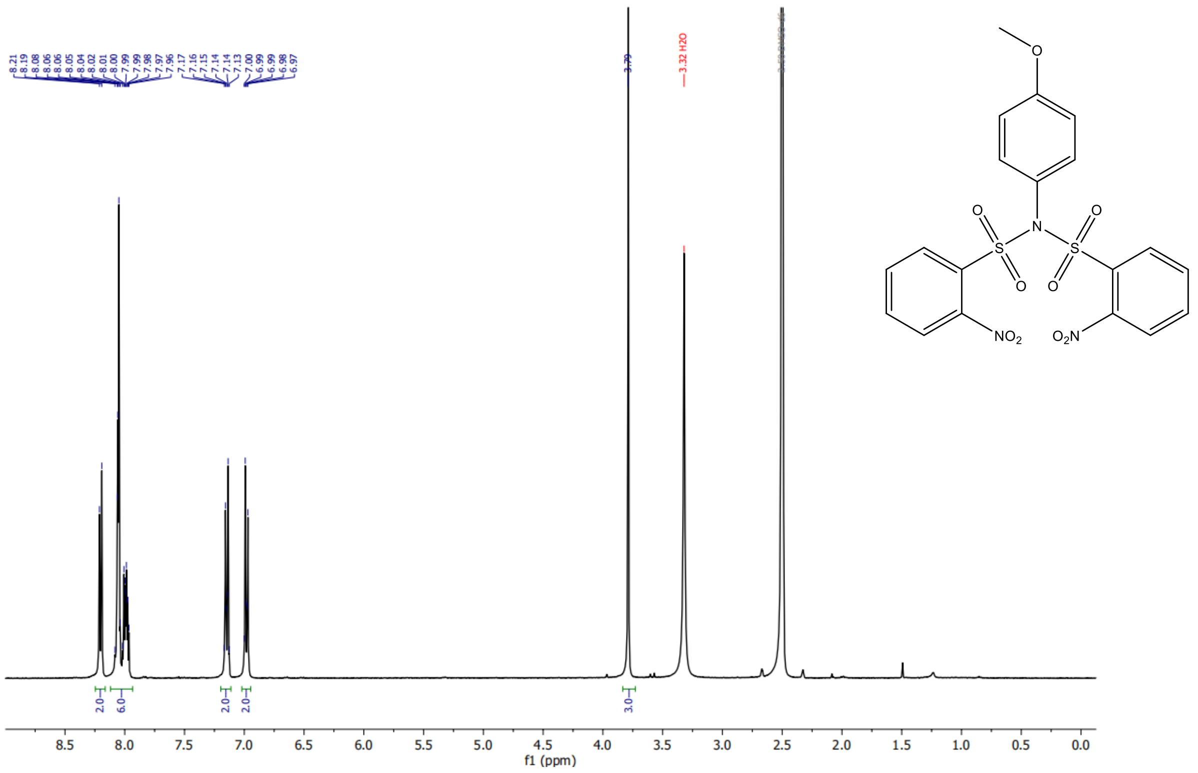

Fig. S14: ^{1}H NMR of compound **3a** (DMSO-*d*$_6$, 400 MHz).

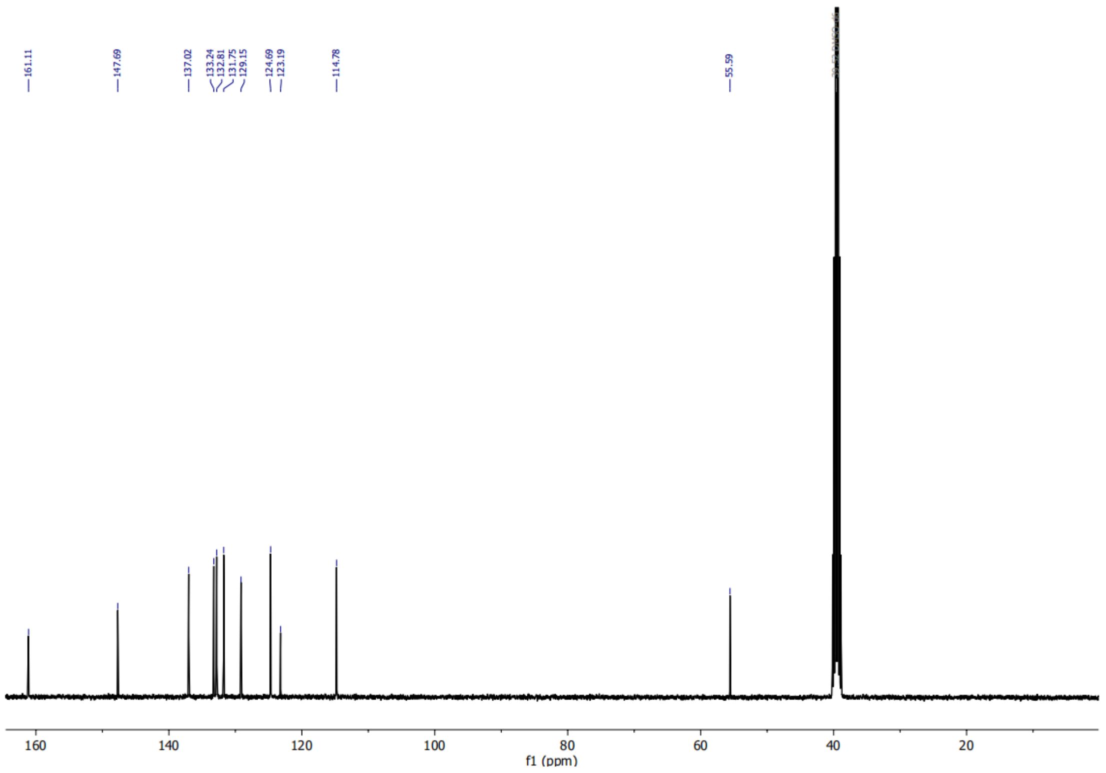

Fig. S15: ^{13}C{^{1}H} NMR of compound **3a** (DMSO-*d*$_6$, 100 MHz).

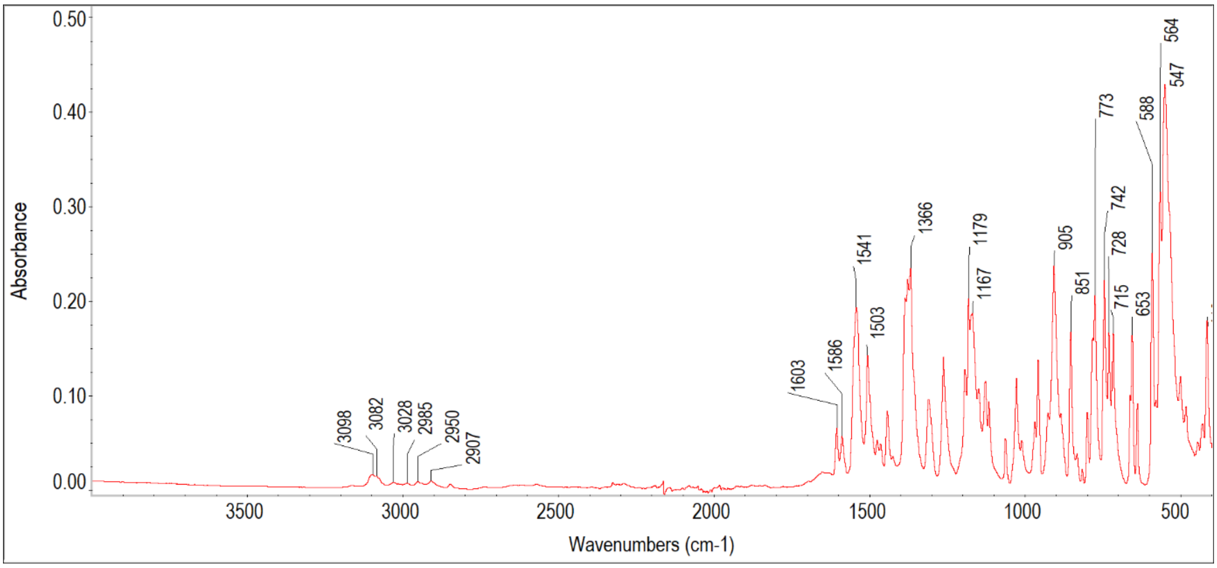

Fig. S16: IR spectrum of compound **3a** (ATR).

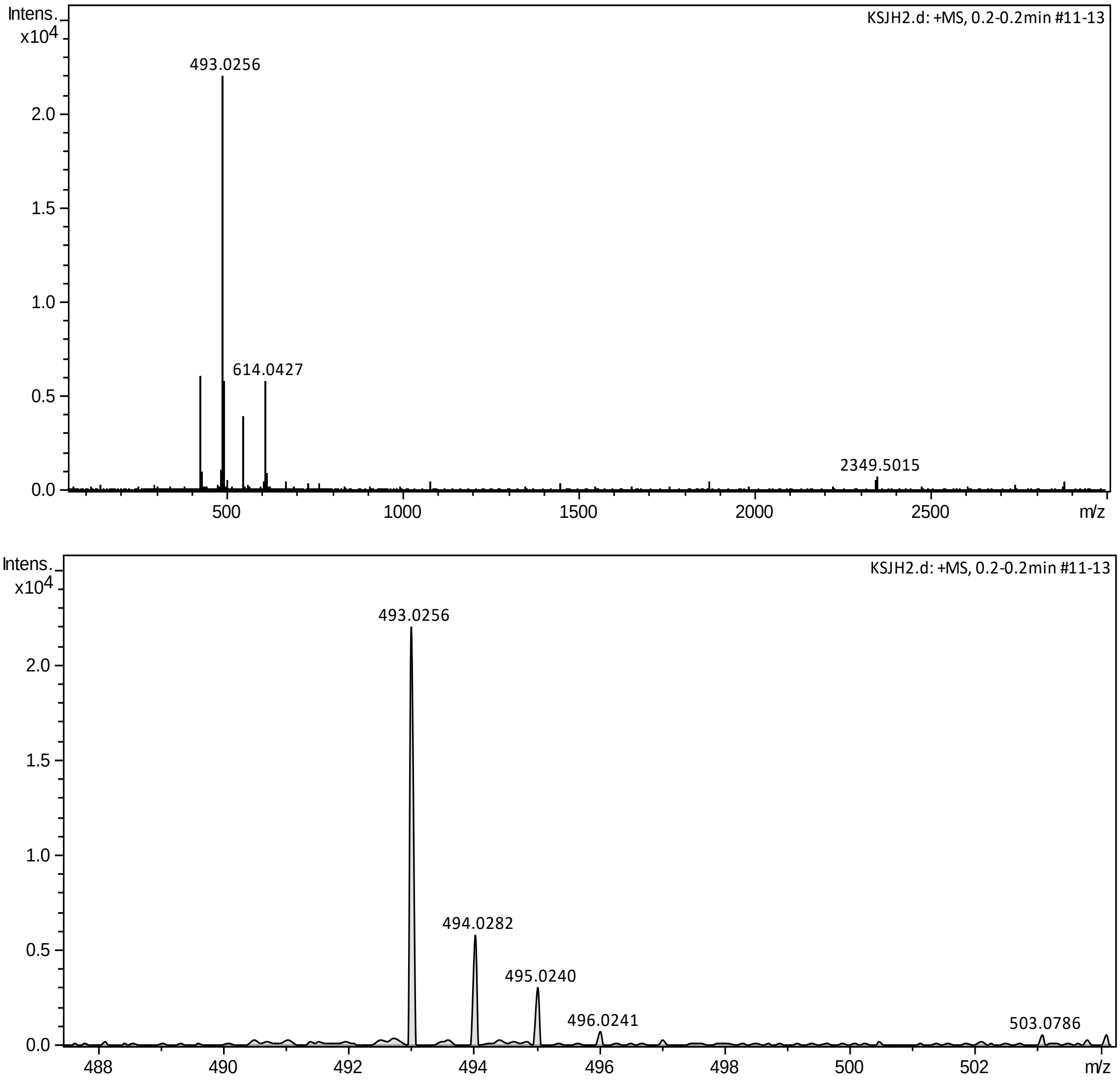

Fig. S17: HRMS of compound **3a** calc $[C_{19}H_{15}N_3O_9S_2]^+$ 493.0244; found *m/z* 493.0256 $[M]^+$.

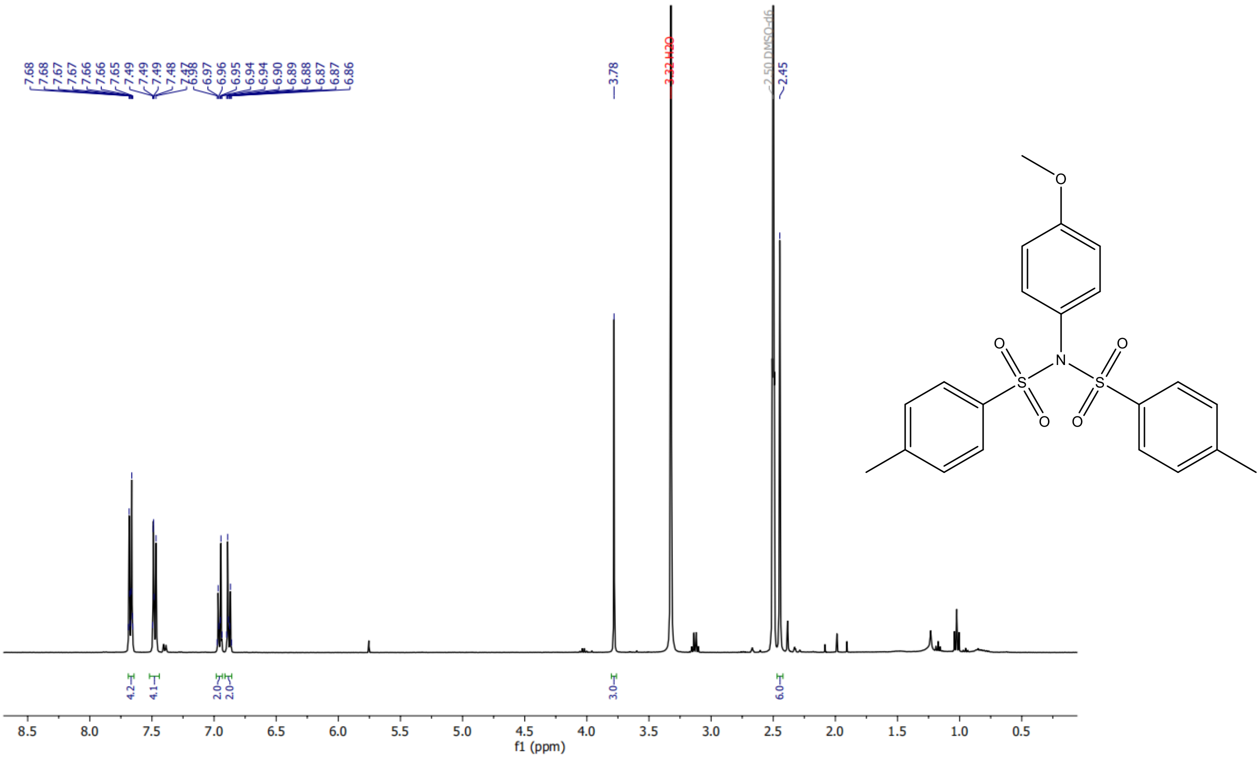

Fig. S18: ^{1}H NMR of compound **3b** (DMSO-d_6, 400 MHz).

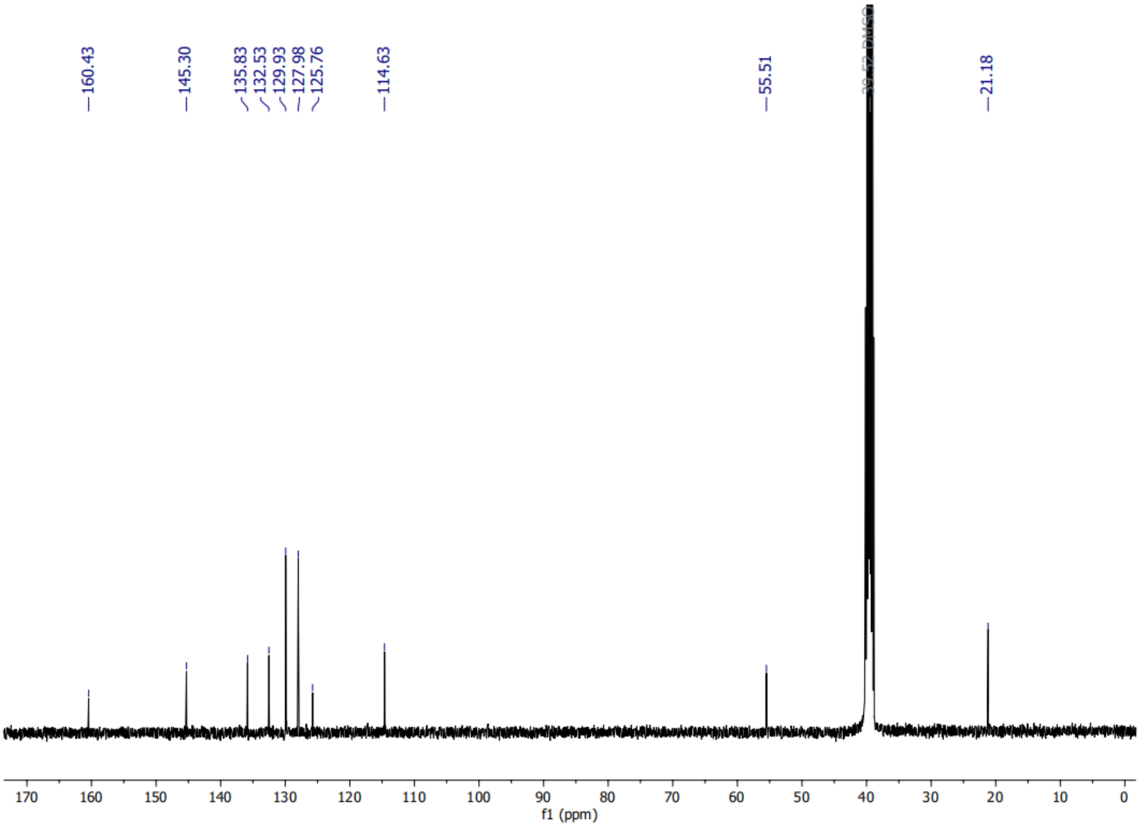

Fig. S19: ^{13}C{^{1}H} NMR of compound **3b** (DMSO-d_6, 100 MHz).

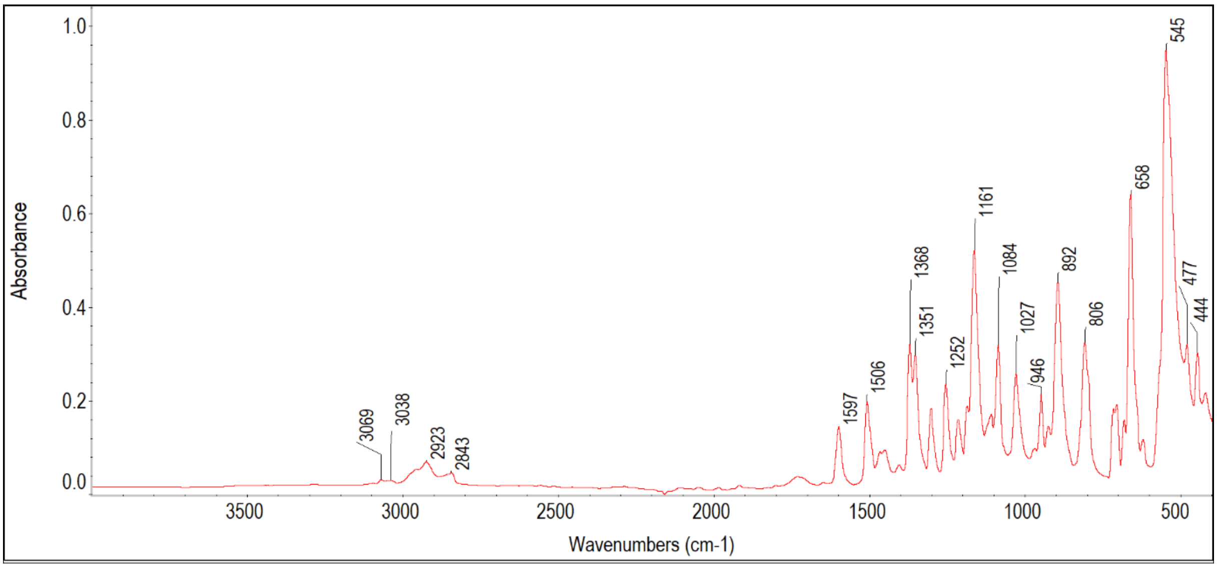

Fig. S20: IR spectrum of compound **3b** (ATR).

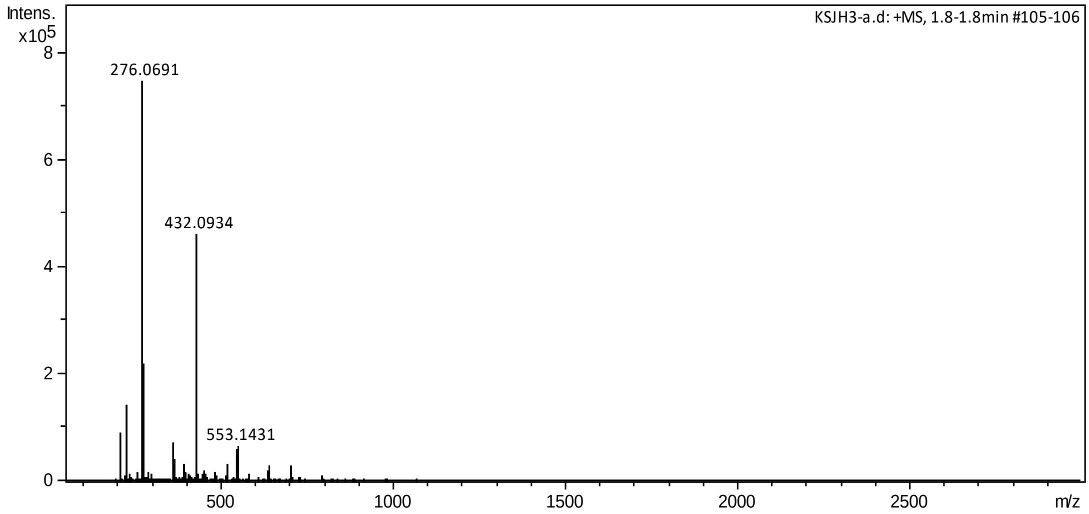

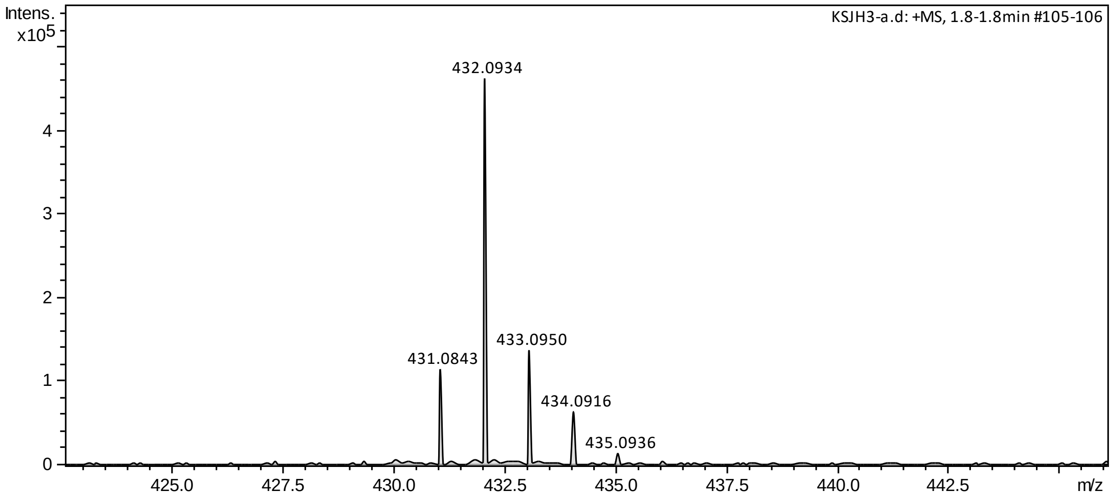

Fig. S21: HRMS of compound **3b** calc $[C_{21}H_{22}NO_5S_2]^+$ 432.0934; found *m/z* 432.0934 $[M+H]^+$.

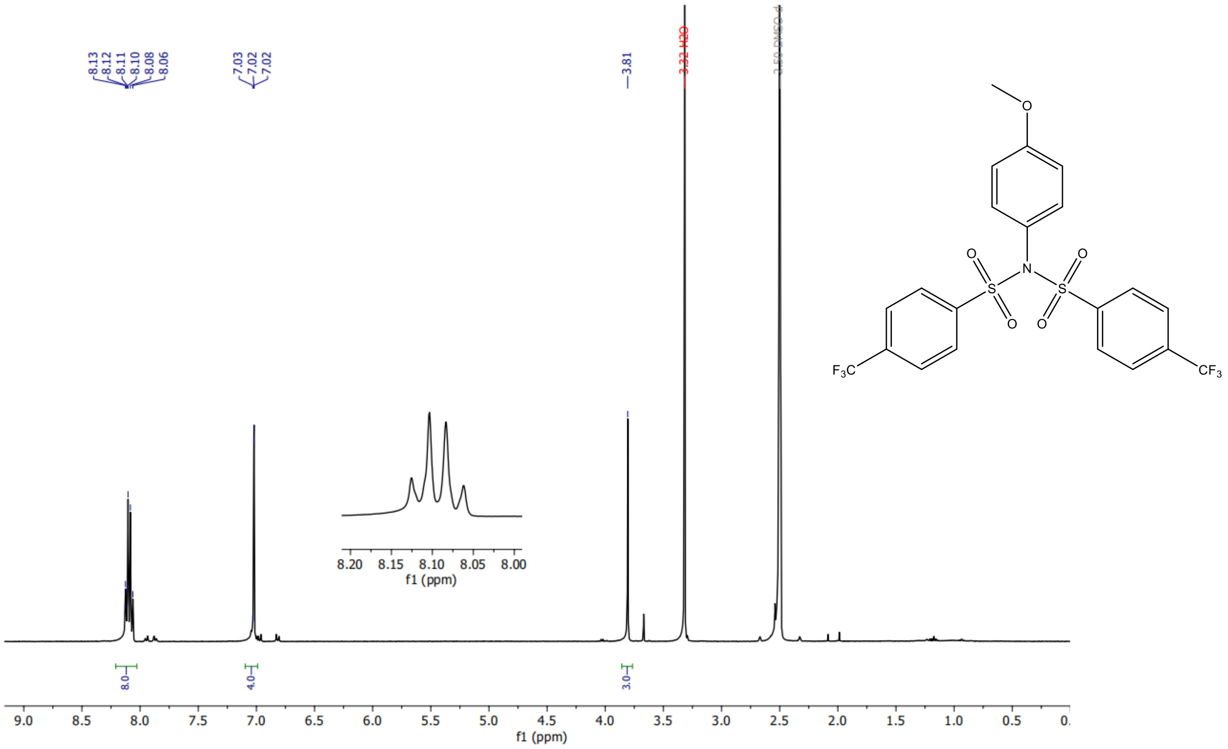

Fig. S22: ^{1}H NMR of compound **3c** (DMSO-*d*$_6$, 400 MHz).

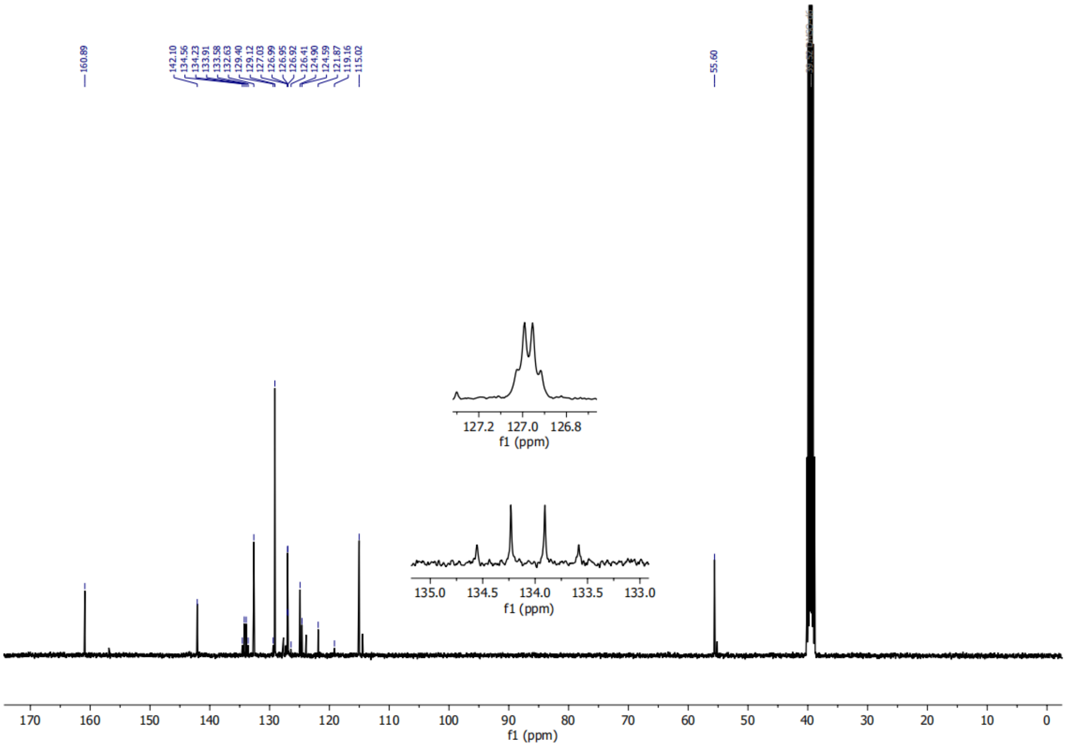

Fig. S23: ^{13}C{^{1}H} NMR of compound **3c** (DMSO-*d*$_6$, 100 MHz).

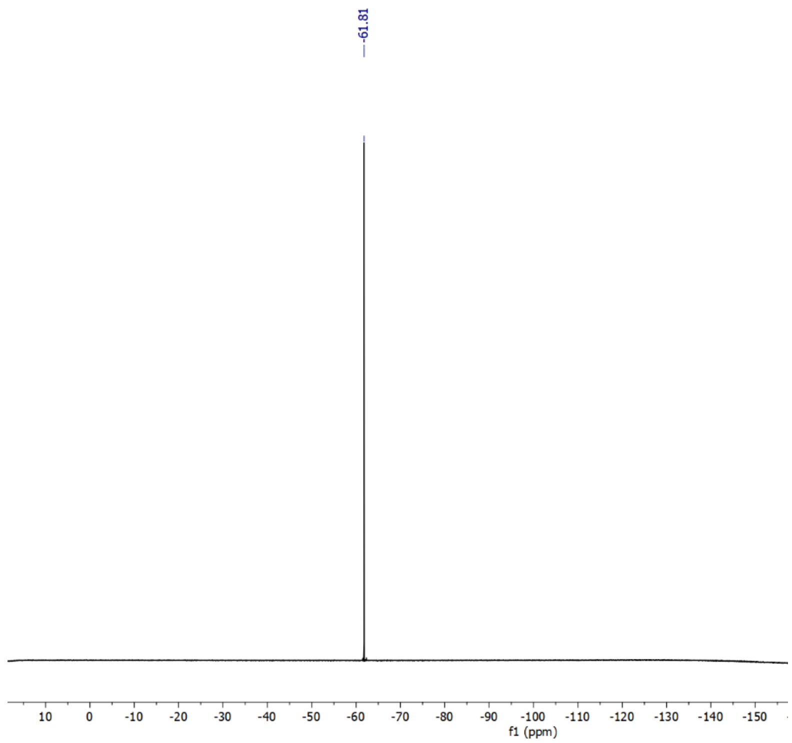

Fig. S24: ^{19}F NMR of compound **3c** (DMSO-d_6, 376 MHz).

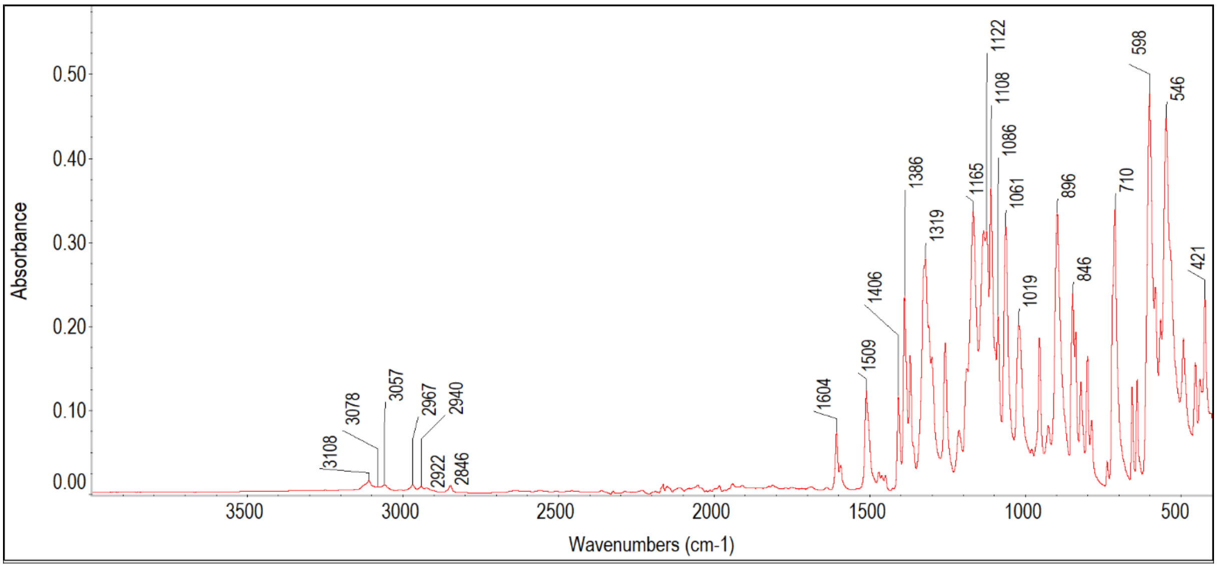

Fig. S25: IR spectrum of compound **3c** (ATR).

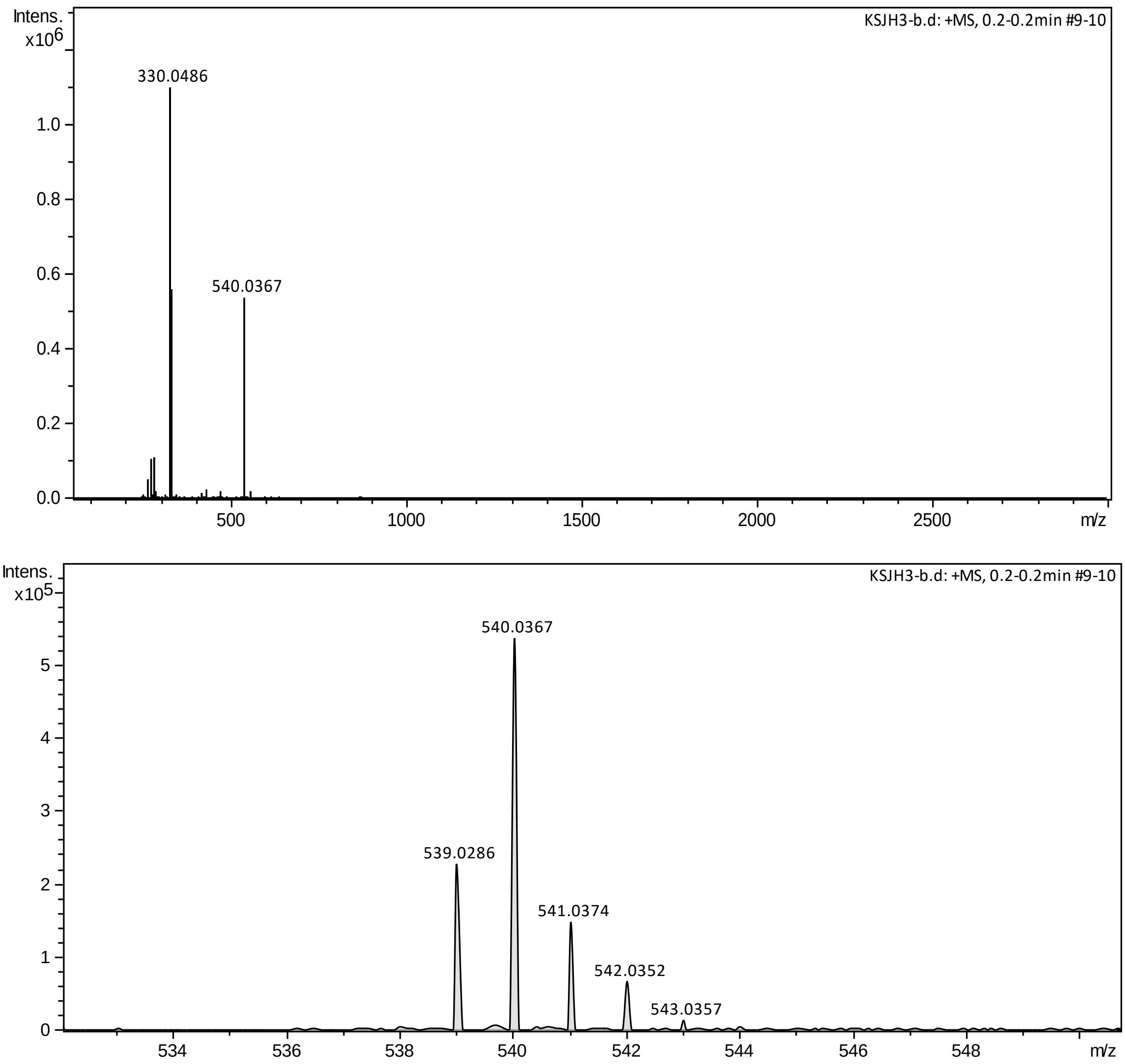

Fig. S26: HRMS of compound **3c** calc $[C_{21}H_{16}F_6NO_5S_2]^+$ 540.0369; found *m/z* 540.0367 $[M+H]^+$.

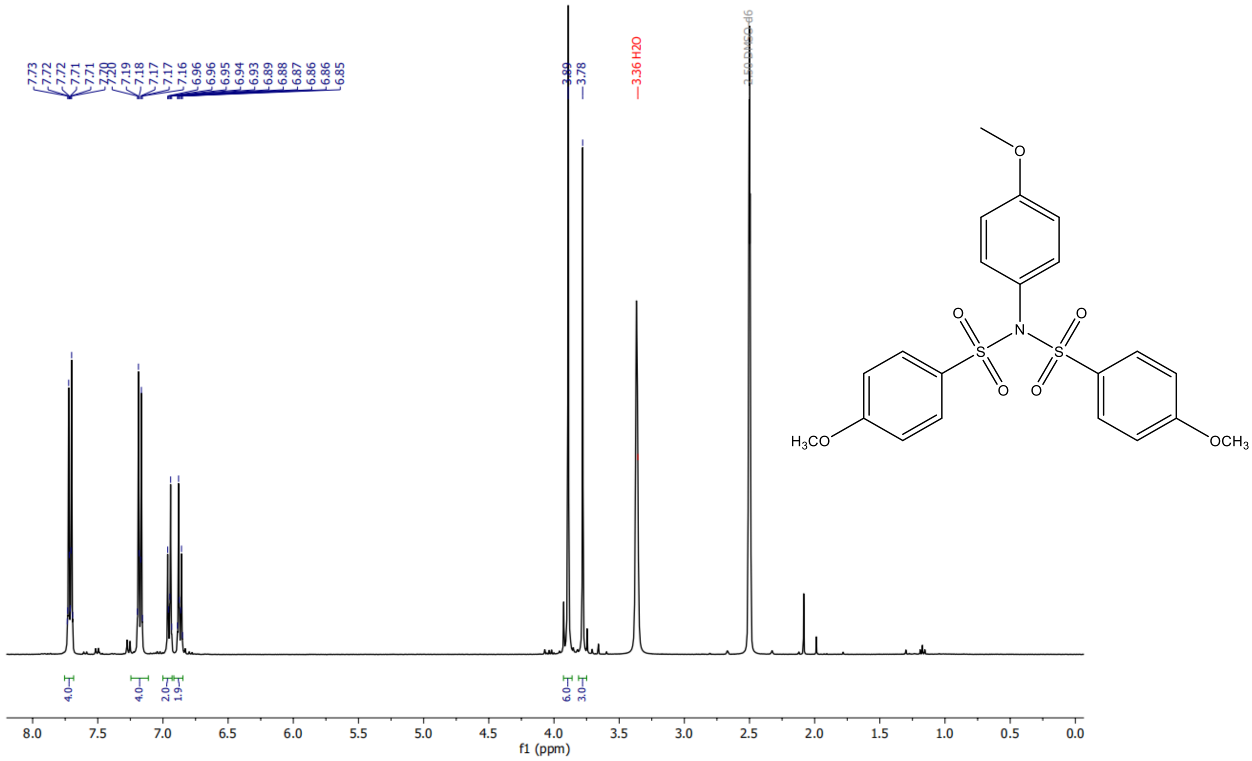

Fig. S27: ^{1}H NMR of compound **3d** (DMSO-d_6, 400 MHz).

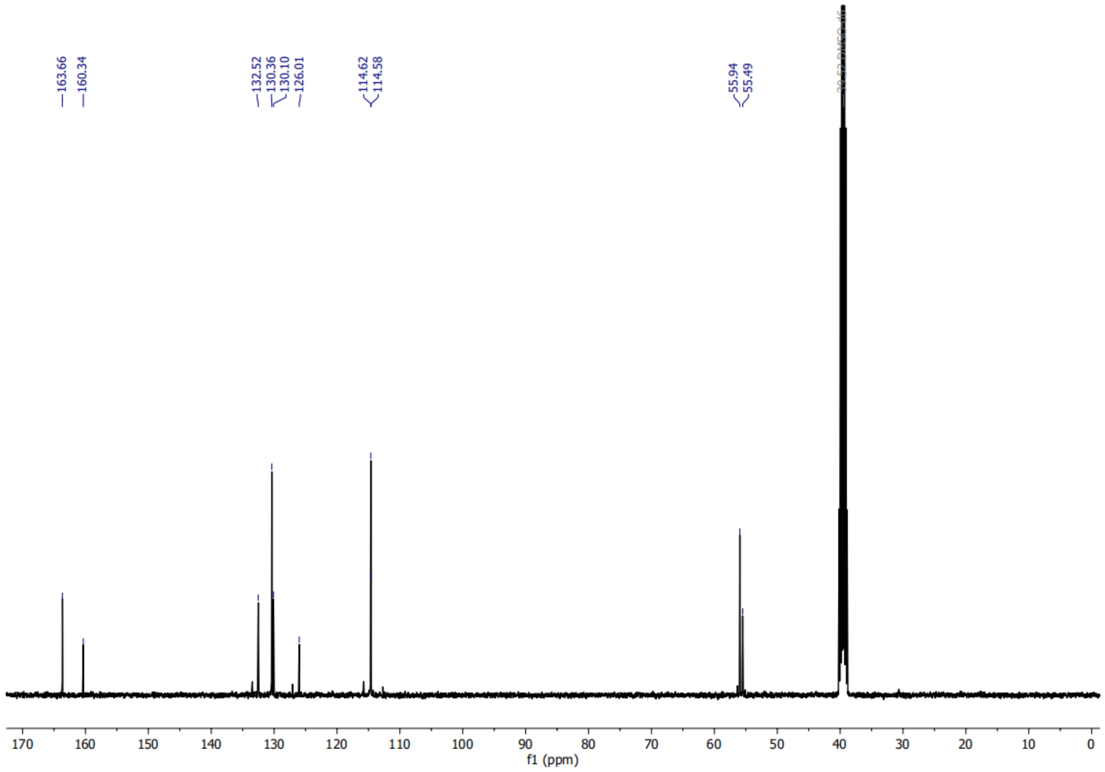

Fig. S28: ^{13}C{^{1}H} NMR of compound **3d** (DMSO-d_6, 100 MHz).

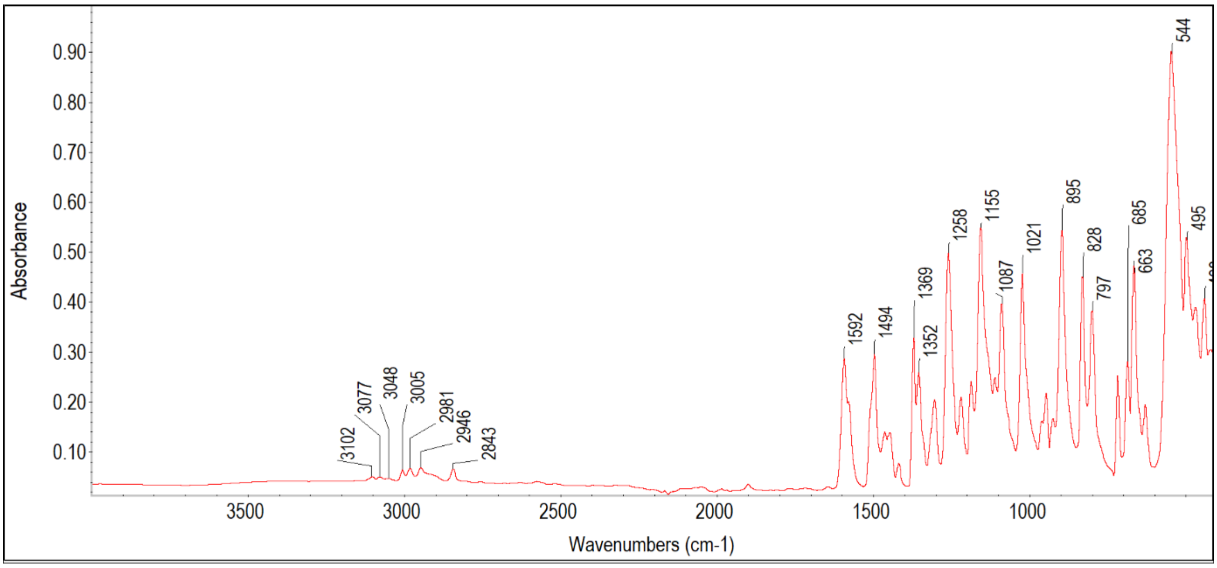

Fig. S29: IR spectrum of compound **3d** (ATR).

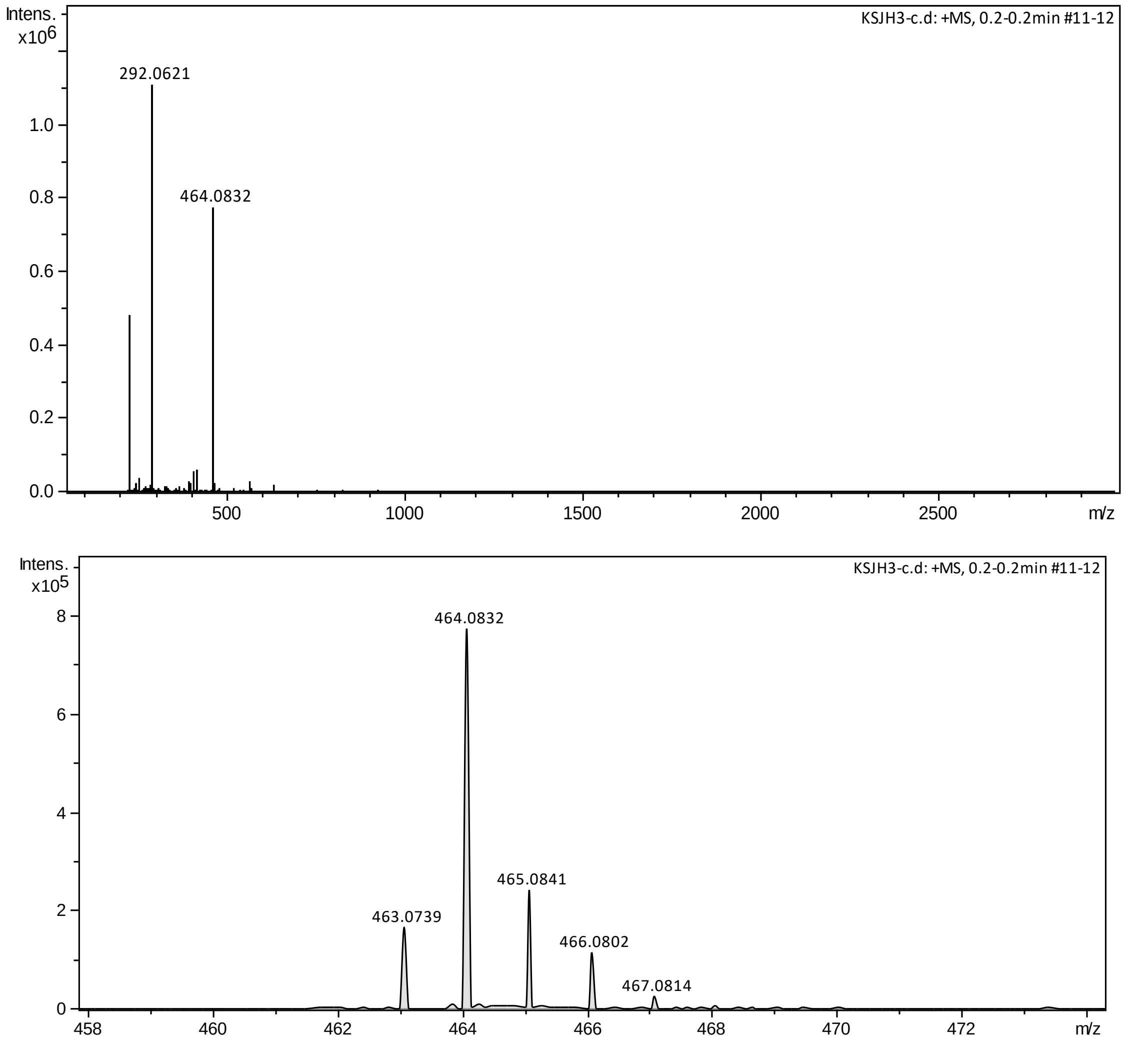

Fig. S30: HRMS of compound **3d** calc $[C_{21}H_{22}NO_7S_2]^+$ 464.0832; found *m/z* 464.0832 $[M+H]^+$.

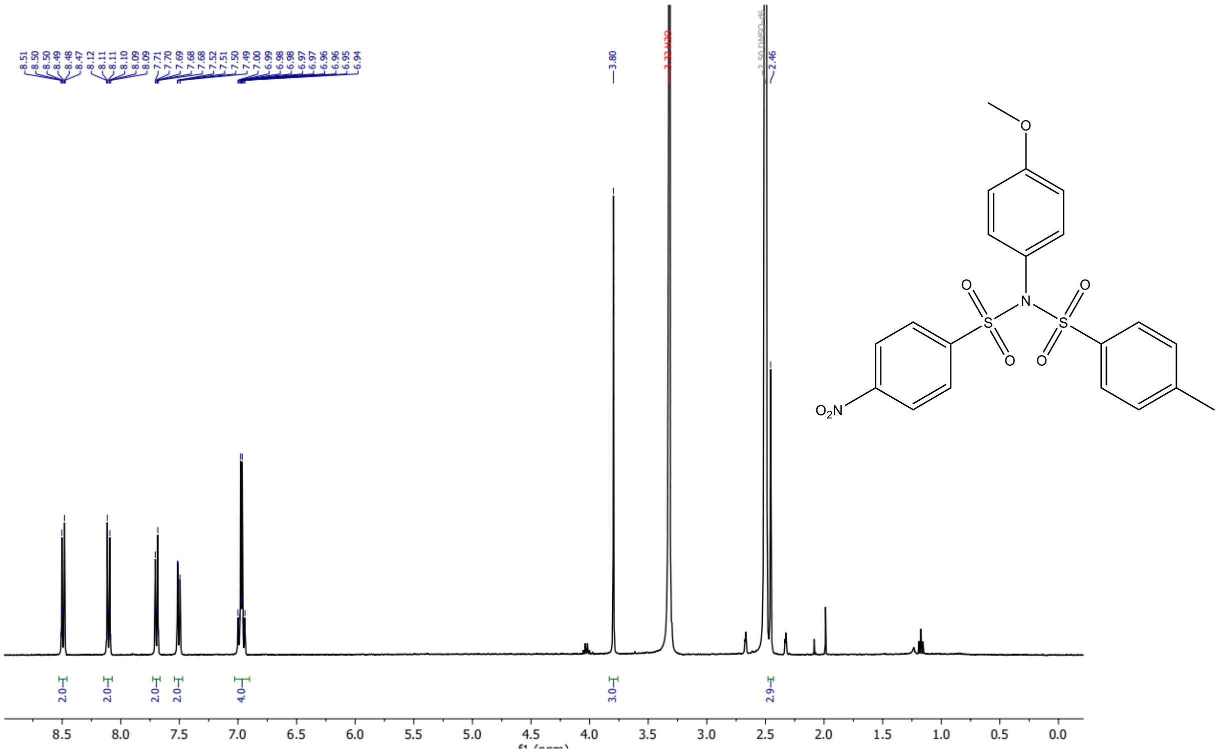

Fig. S31: ^{1}H NMR of compound **4a** (DMSO-d_6, 400 MHz).

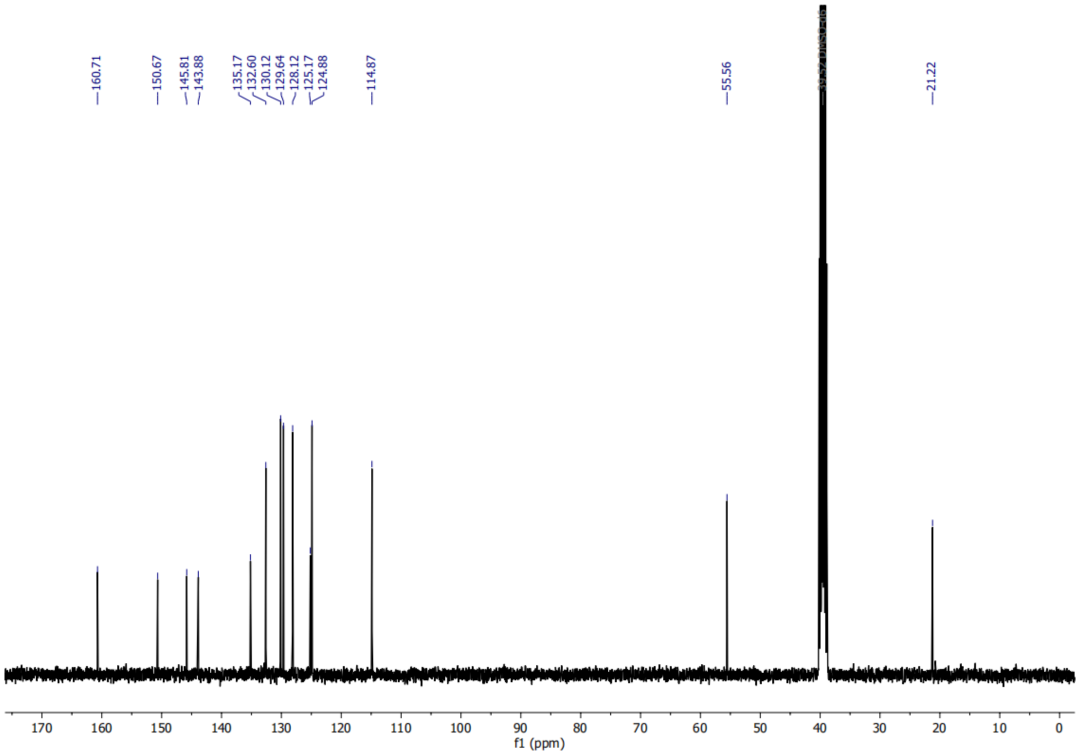

Fig. S32: ^{13}C{^{1}H} NMR of compound **4a** (DMSO-d_6, 100 MHz).

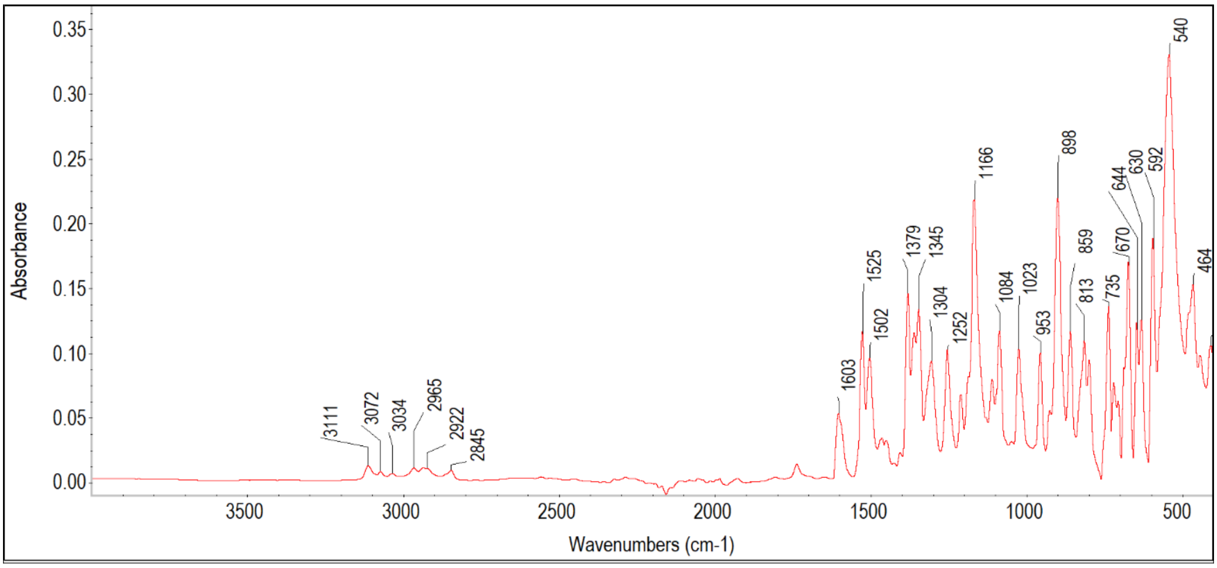

Fig. S33: IR spectrum of compound **4a** (ATR).

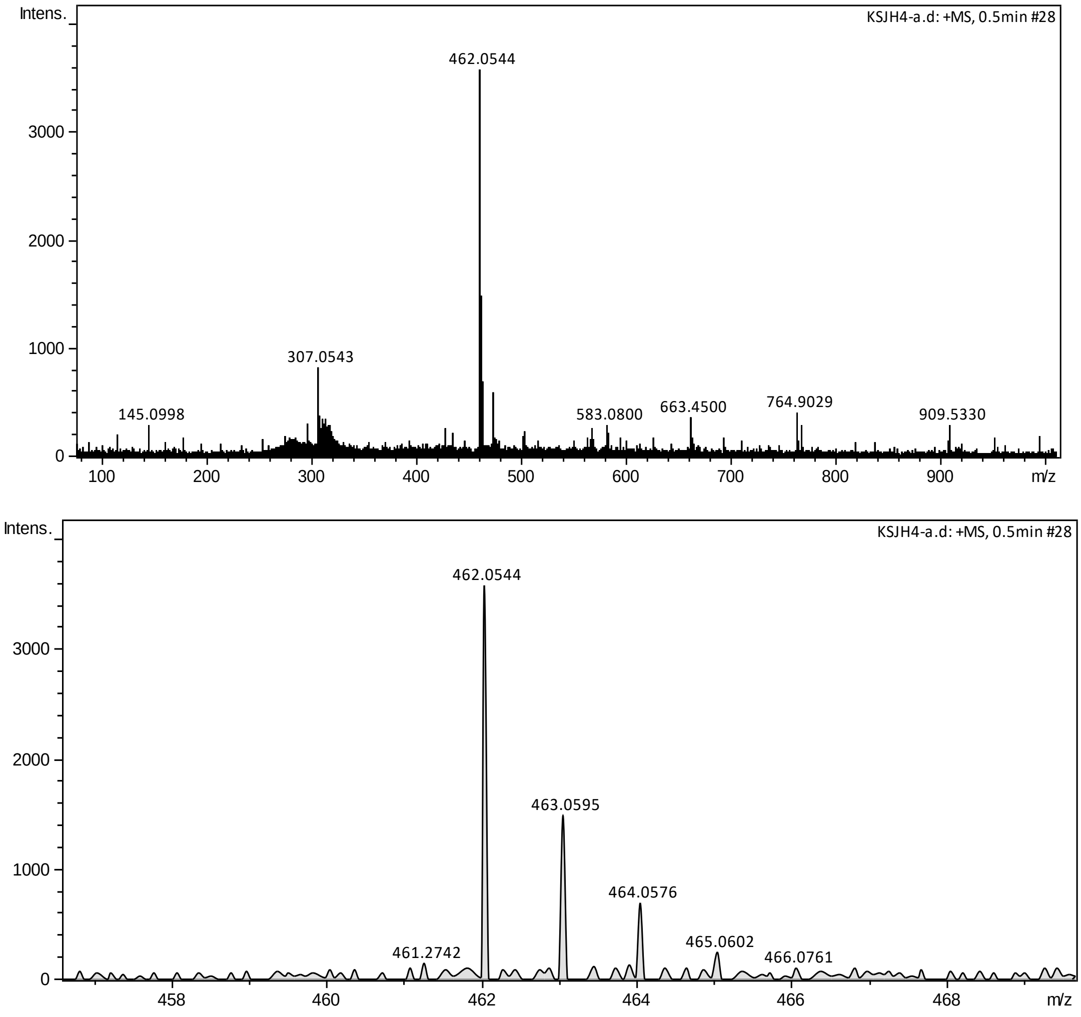

Fig. S34: HRMS of compound **4a** calc $[C_{20}H_{18}N_2O_7S_2]^+$ 462.0550; found *m/z* 462.0544 $[M]^+$.

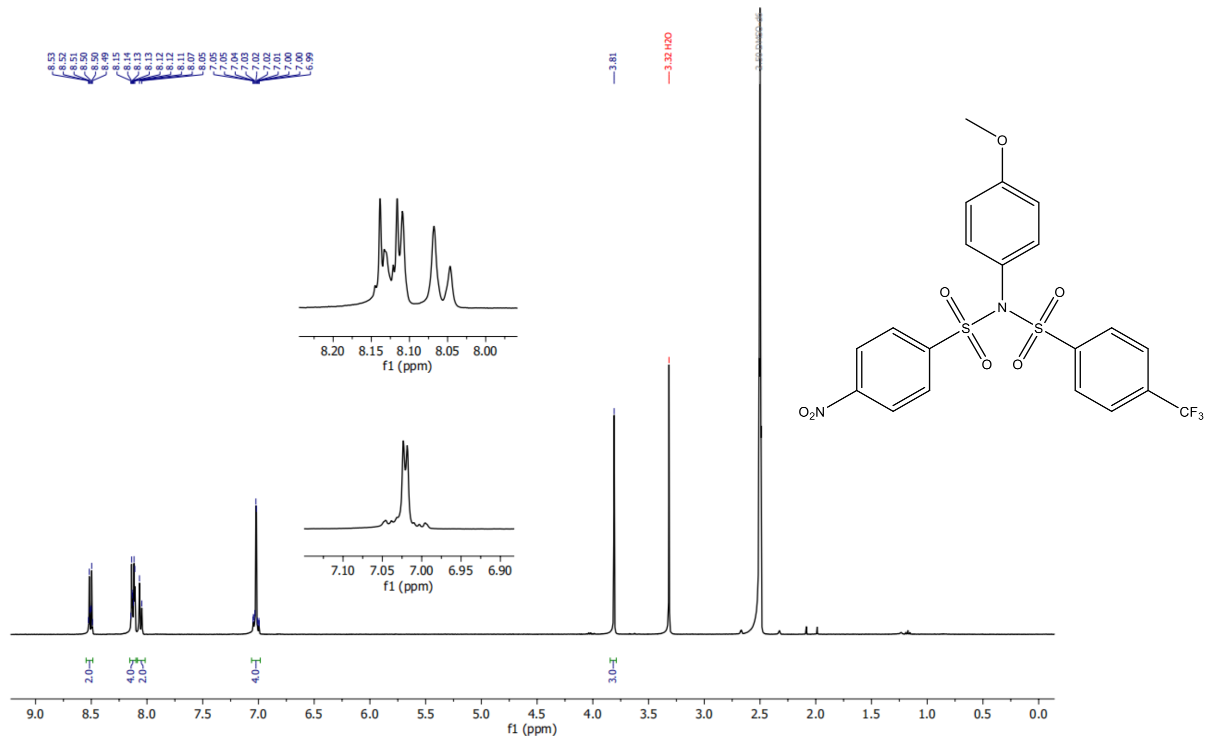

Fig. S35: ^{1}H NMR of compound **4b** (DMSO-d_6, 400 MHz).

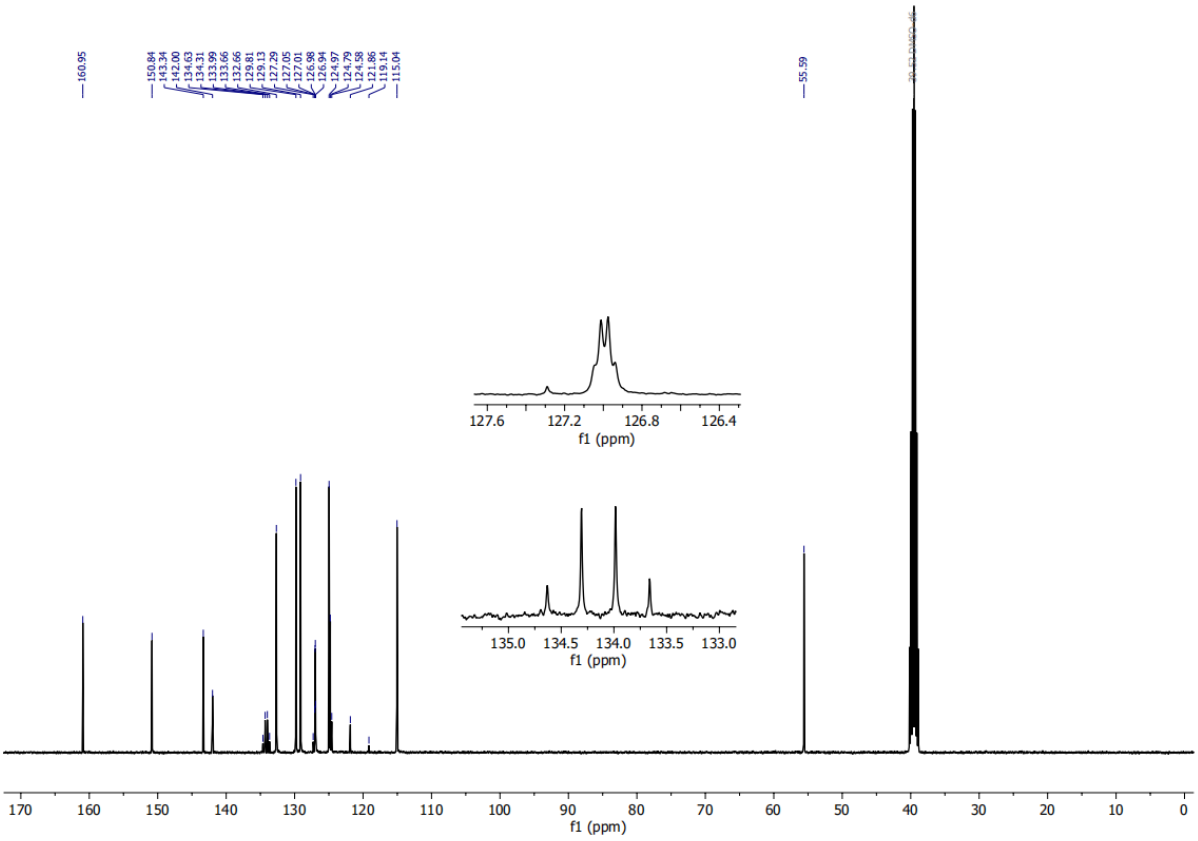

Fig. S36: ^{13}C{^{1}H} NMR of compound **4b** (DMSO-d_6, 100 MHz).

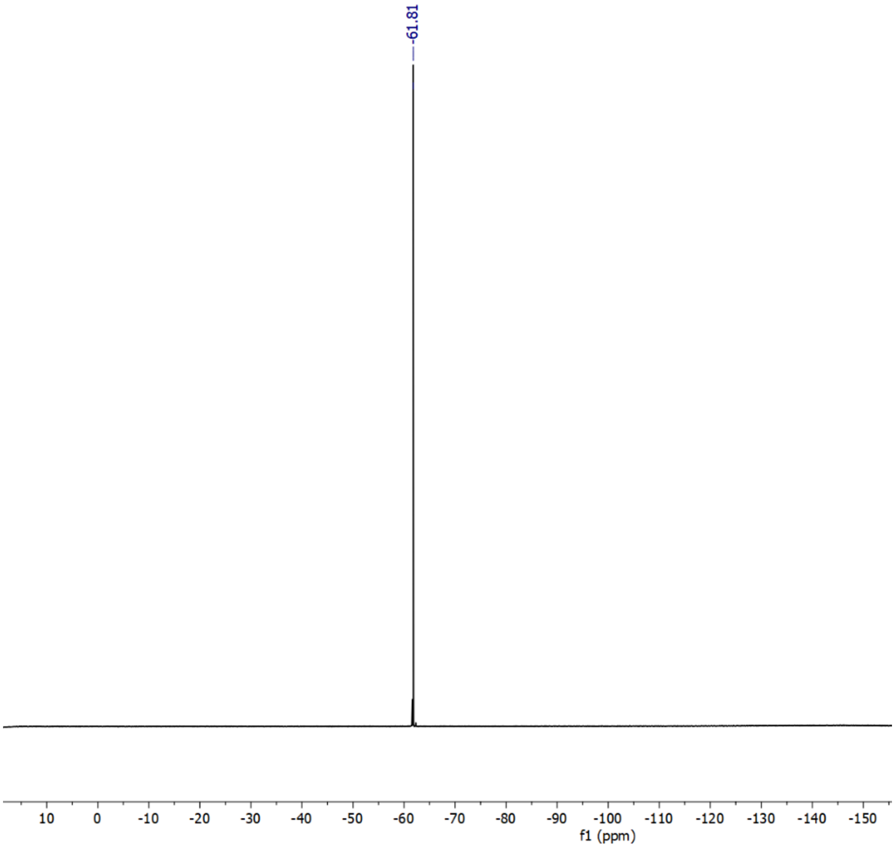

Fig. S37: ^{19}F NMR of compound **4b** (DMSO-d_6, 376 MHz).

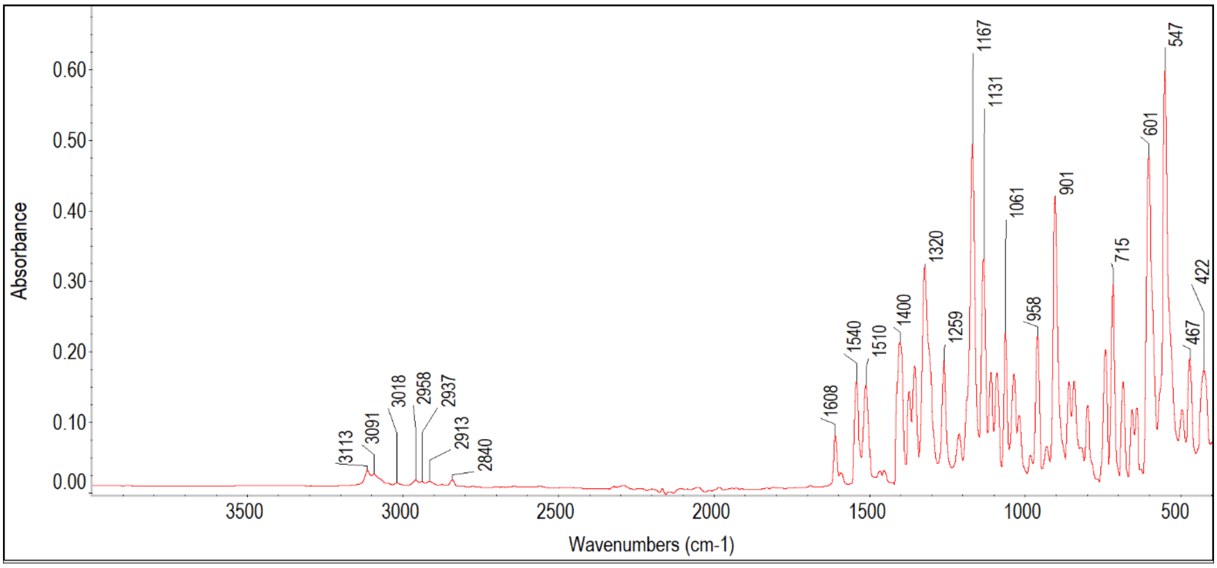

Fig. S38: IR spectrum of compound **4b** (ATR).

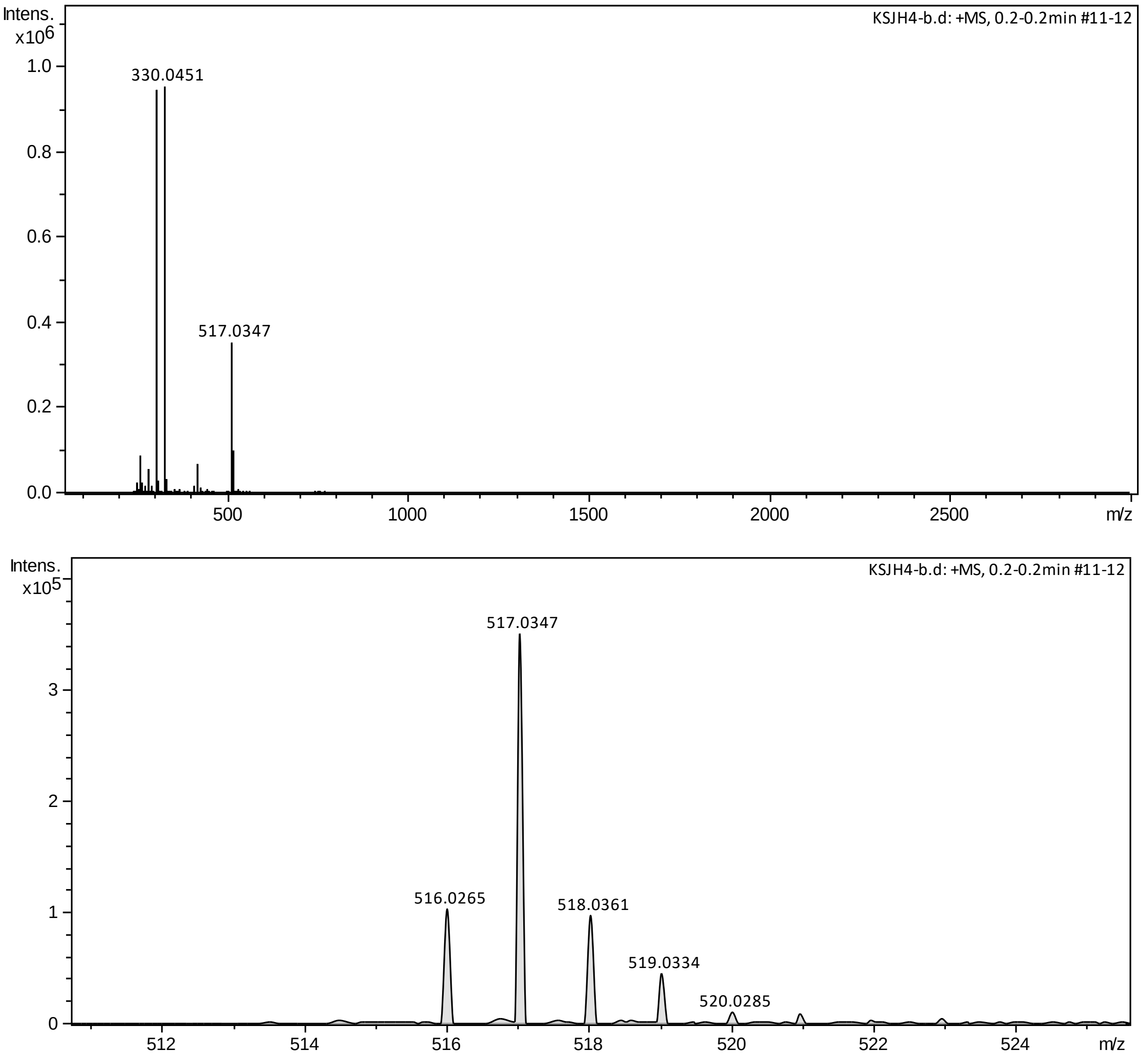

Fig. S39: HRMS of compound **4b** calc $[C_{20}H_{16}F_3N_2O_7S_2]^+$ 517.0346; found *m/z* 517.0347 $[M+H]^+$.

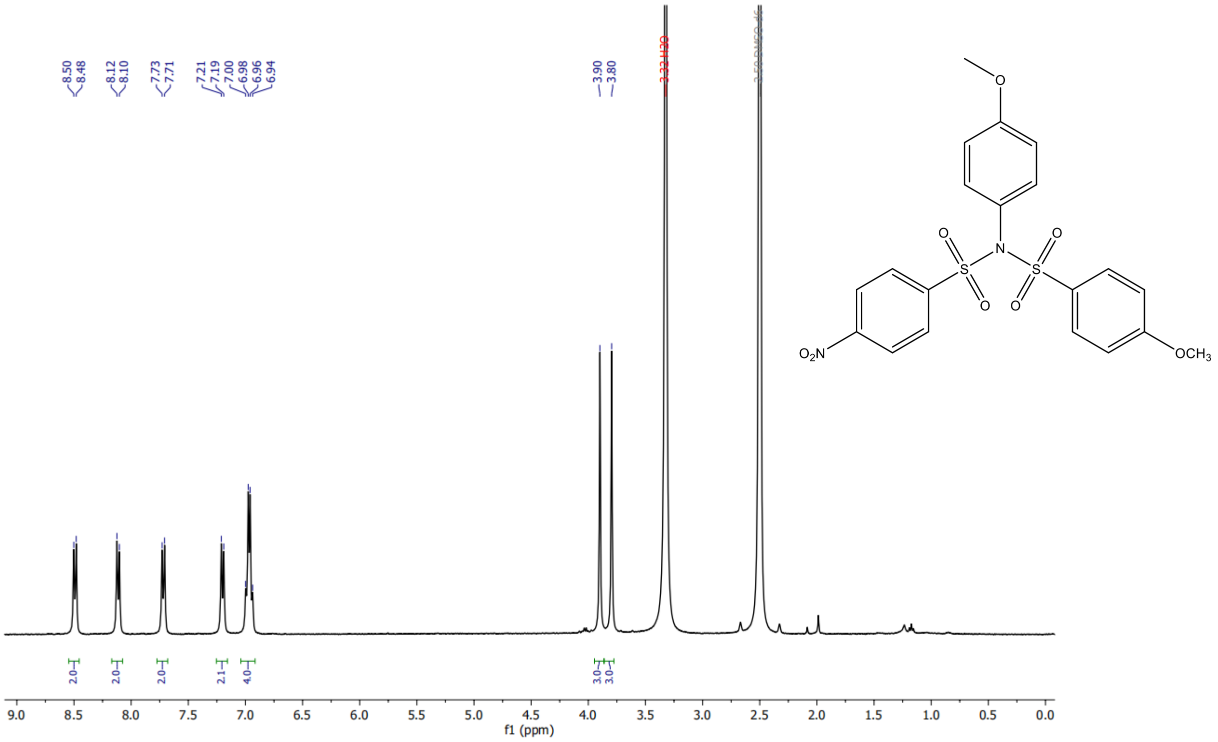

Fig. S40: ^{1}H NMR of compound **4c** (DMSO-d_6, 400 MHz).

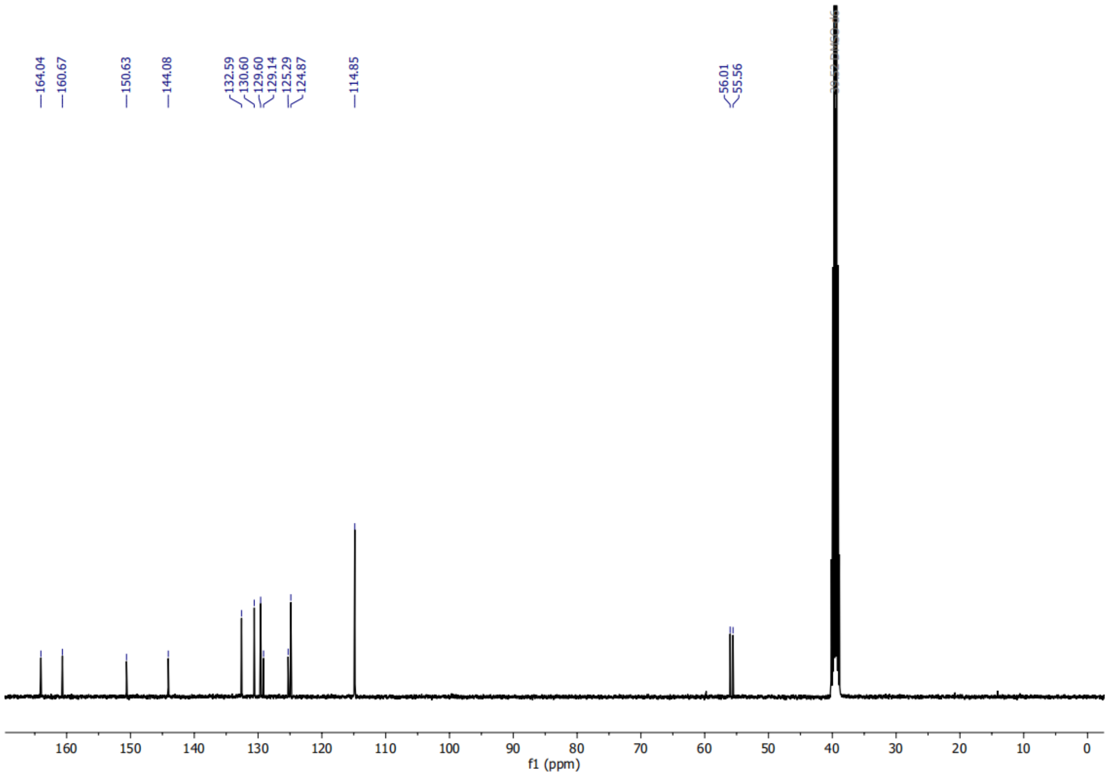

Fig. S41: ^{13}C{^{1}H} NMR of compound **4c** (DMSO-d_6, 100 MHz).

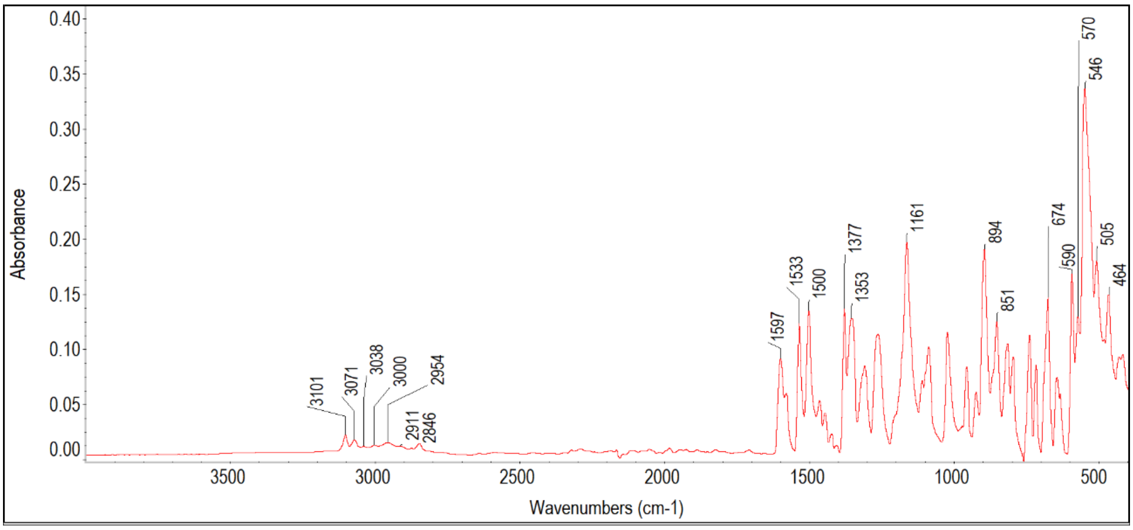

Fig. S42: IR spectrum of compound **4c** (ATR).

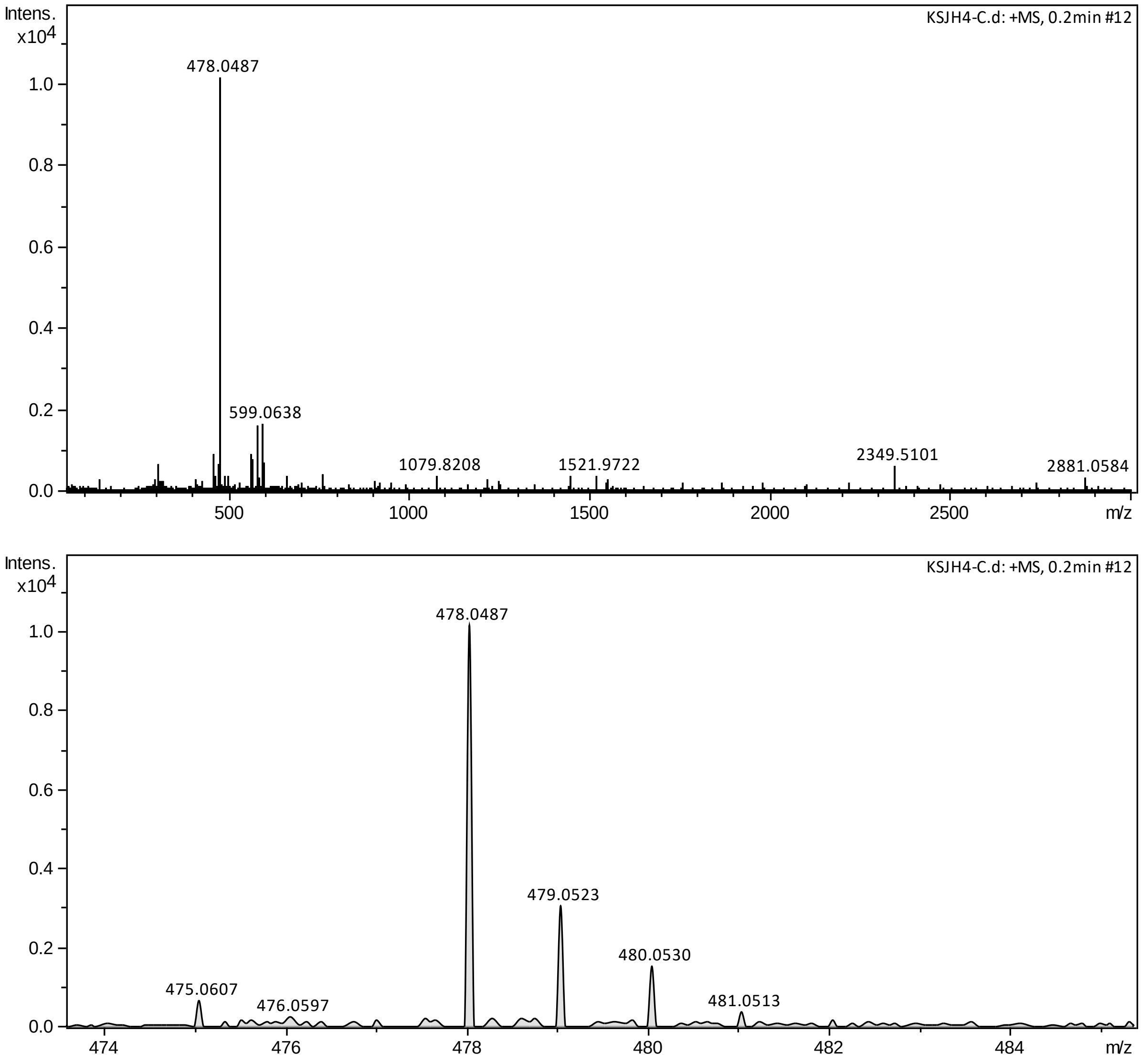

Fig. S43: HRMS of compound **4c** calc $[C_{20}H_{18}N_2O_8S_2]^+$ 478.0499; found *m/z* 478.0487 $[M]^+$.

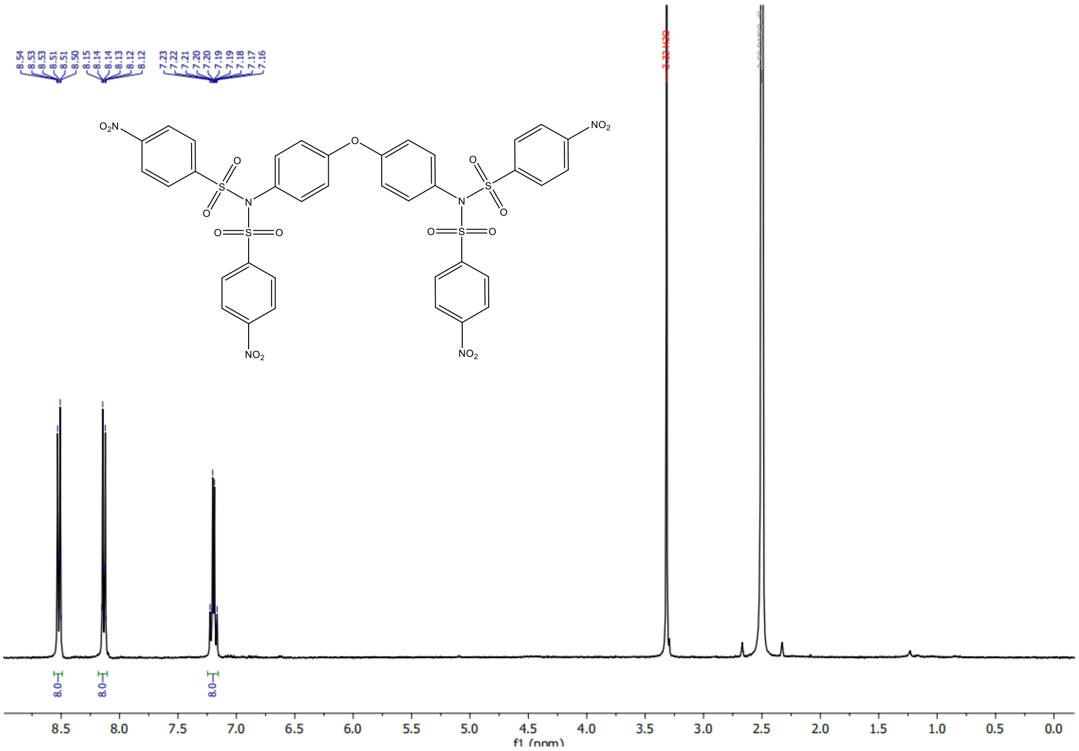

Fig. S44: ^{1}H NMR of compound **6** (DMSO-d_6, 400 MHz).

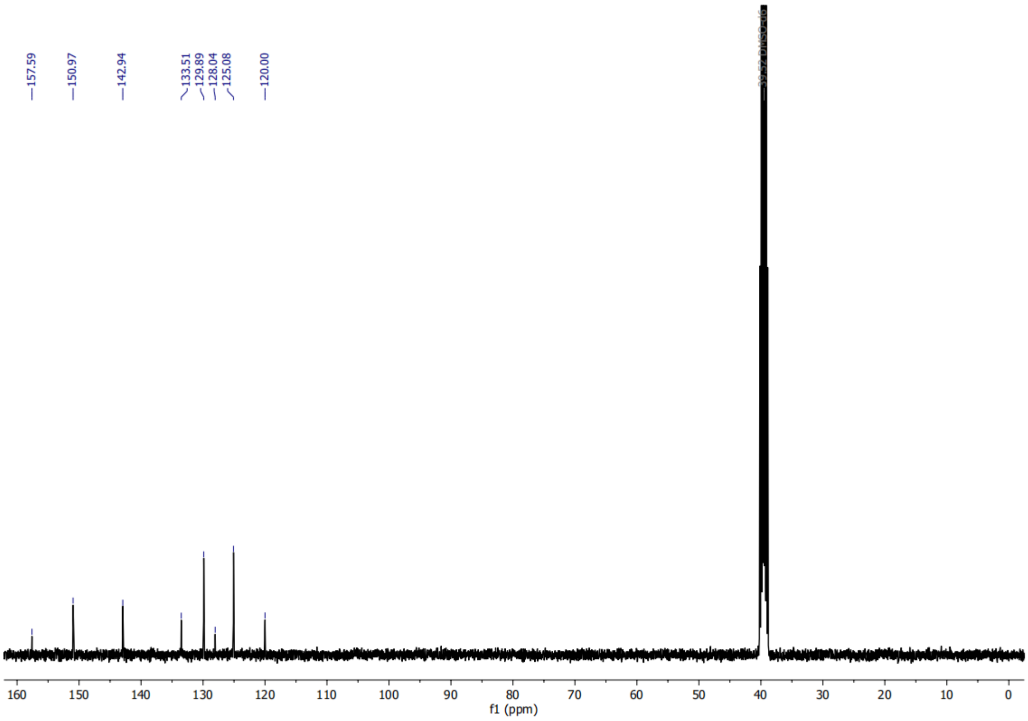

Fig. S45: ^{13}C{^{1}H} NMR of compound **6** (DMSO-d_6, 100 MHz).

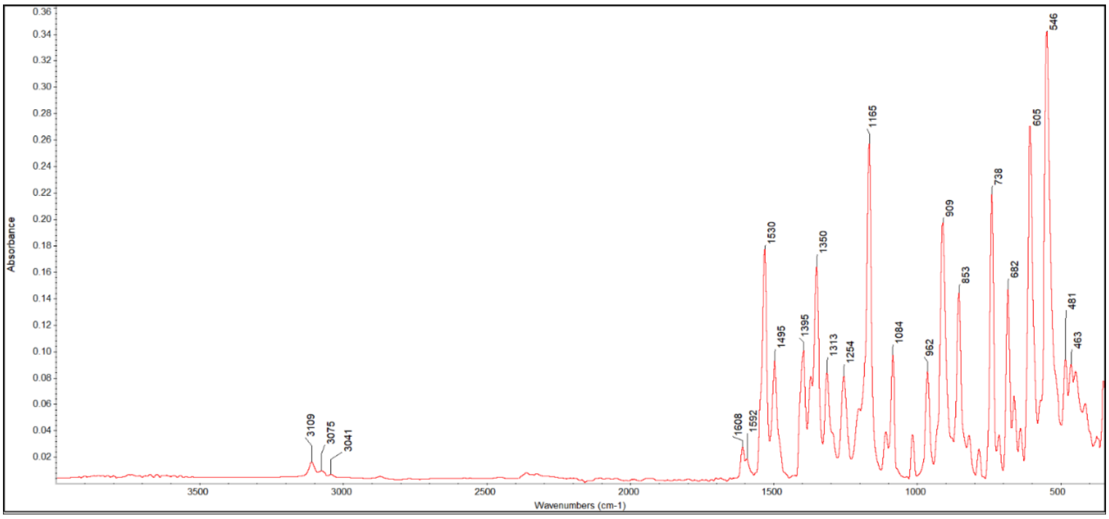

Fig. S46: IR spectrum of compound **6** (ATR).

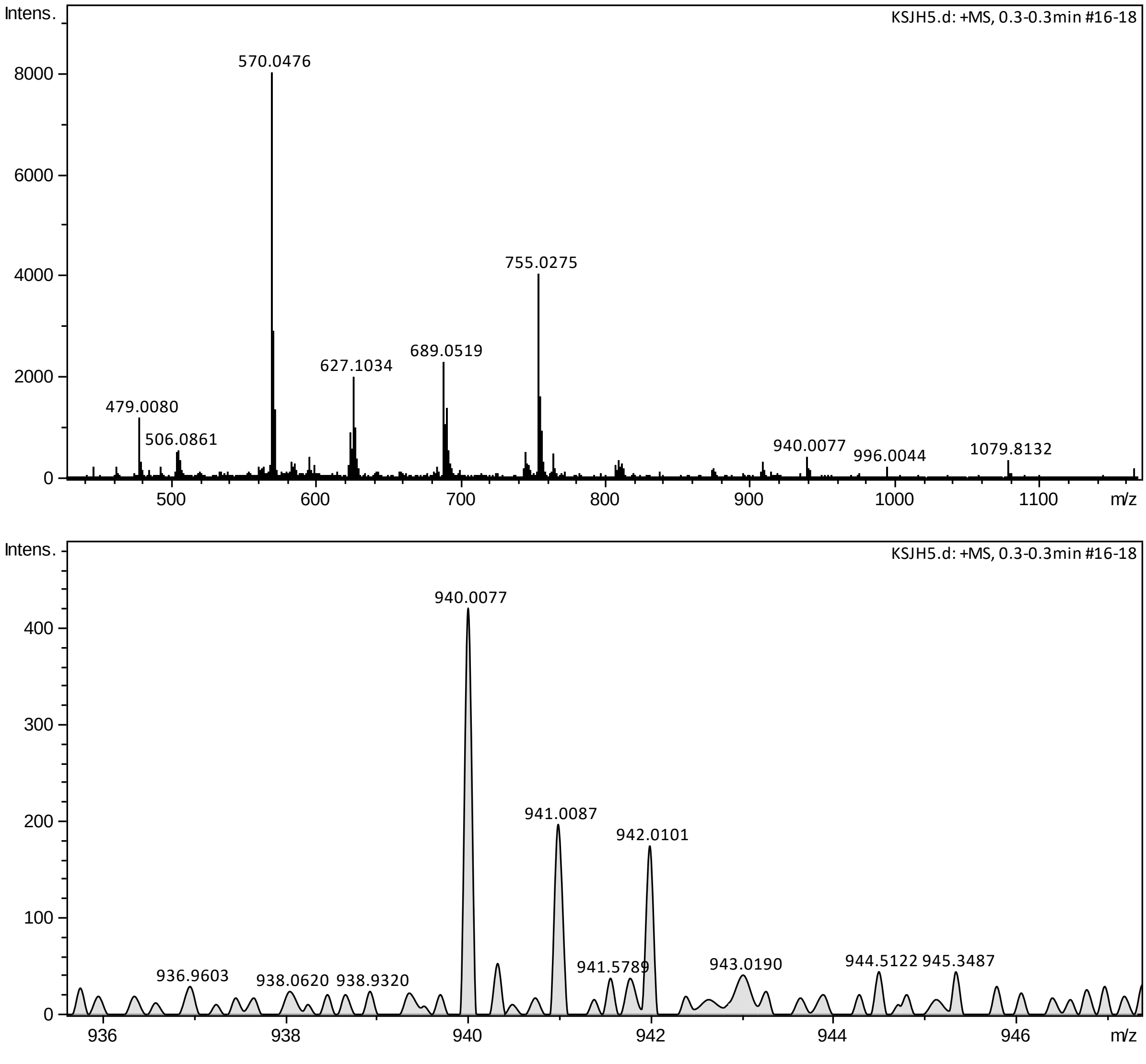

Fig. S47: HRMS of compound **6** calc $[C_{36}H_{24}N_6O_{17}S_4]^+$ 940.0075; found *m/z* 940.0077 $[M]^+$.

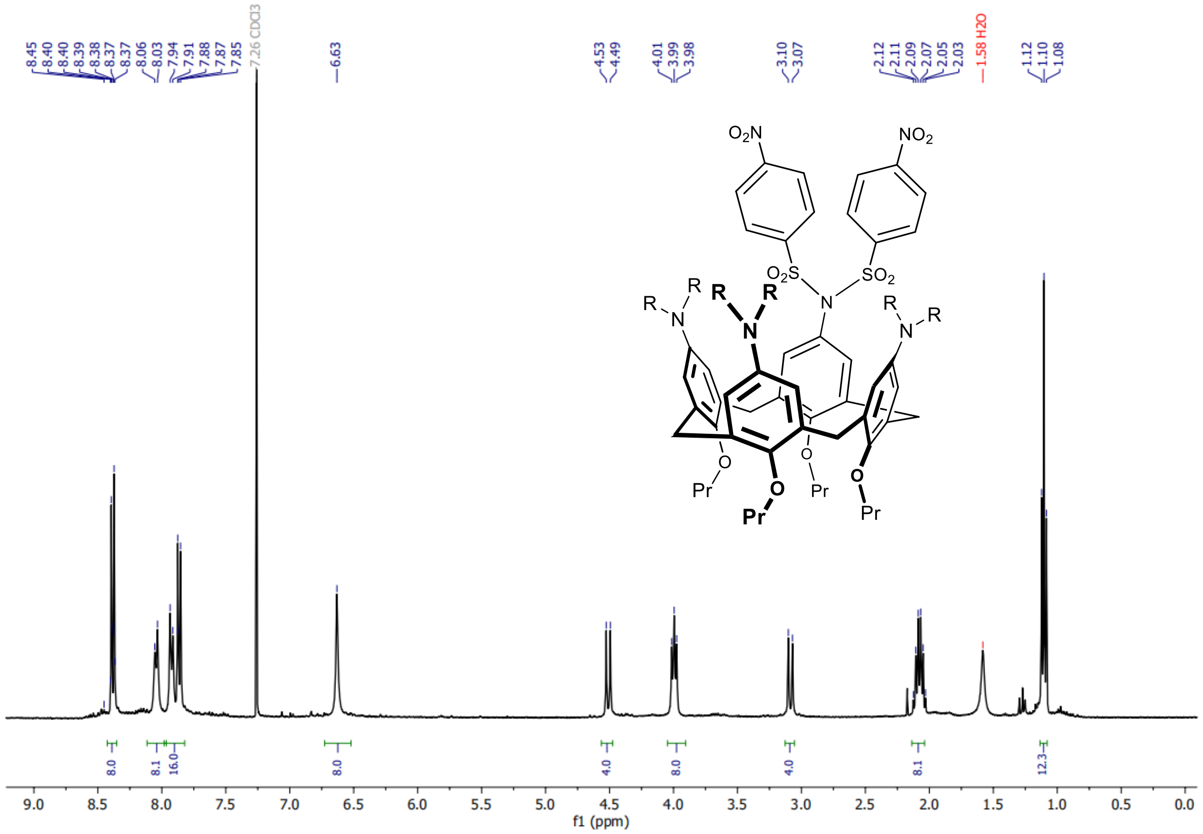

Fig. S48: ^{1}H NMR of compound **7** ($CDCl_3$, 400 MHz).

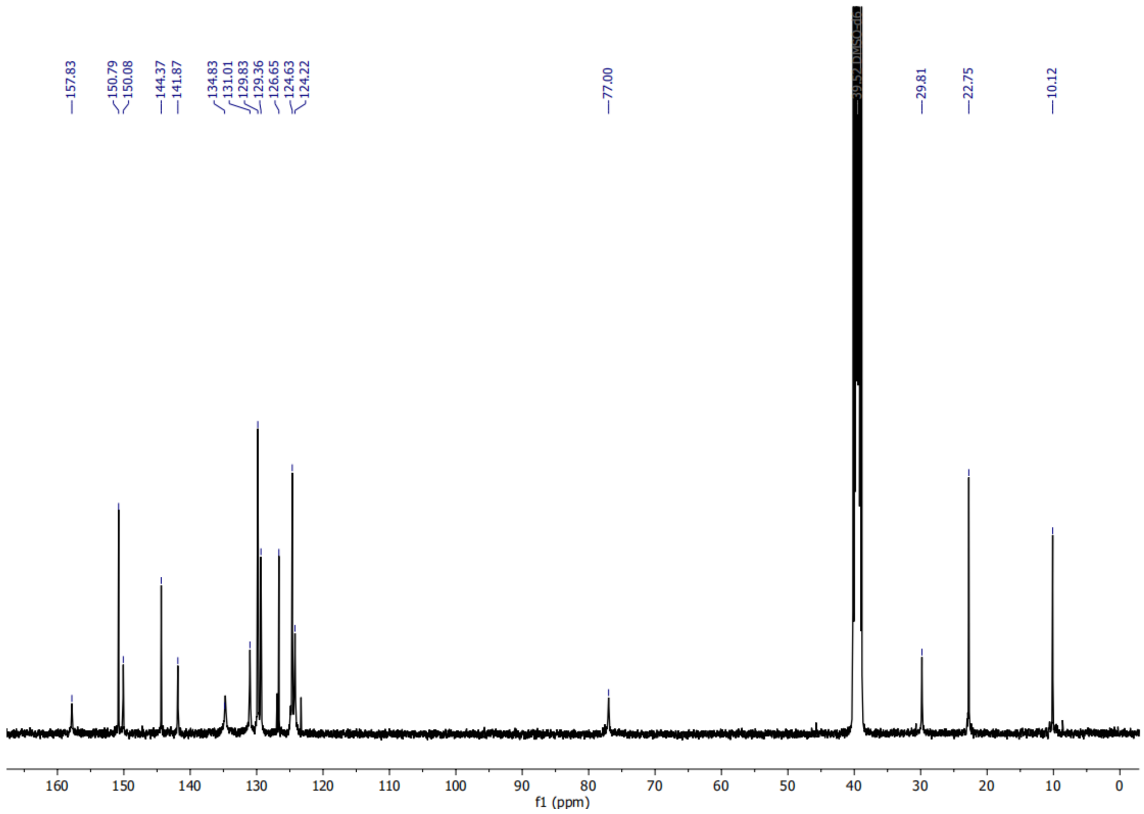

Fig. S49: $^{13}C\{^{1}H\}$ NMR of compound **7** (DMSO-d_6, 100 MHz).

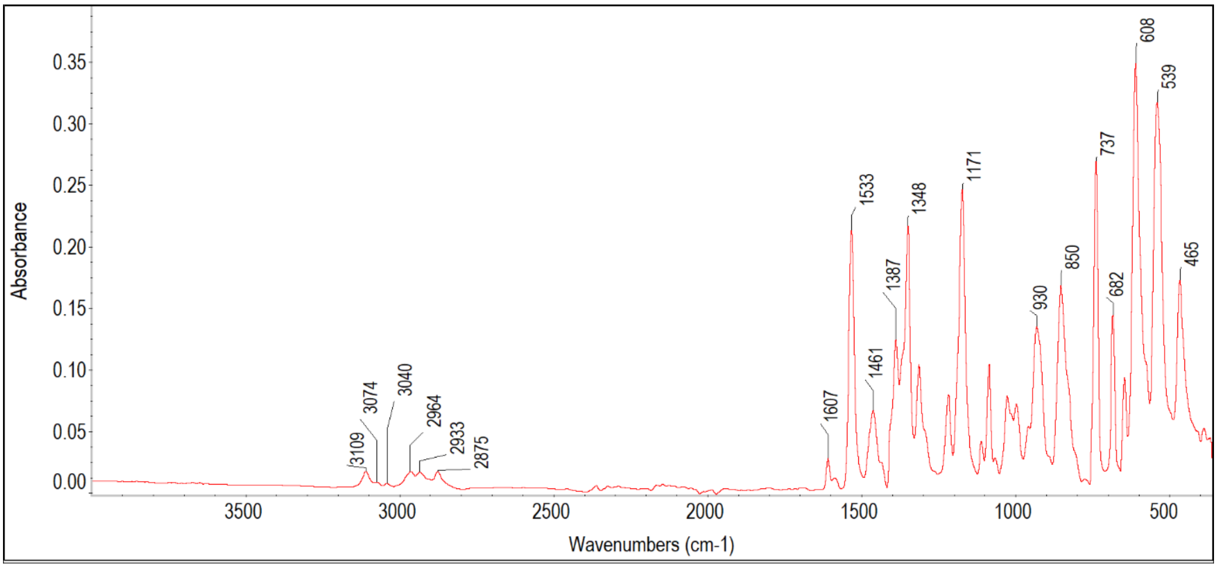

Fig. S50: IR spectrum of compound **7** (ATR).

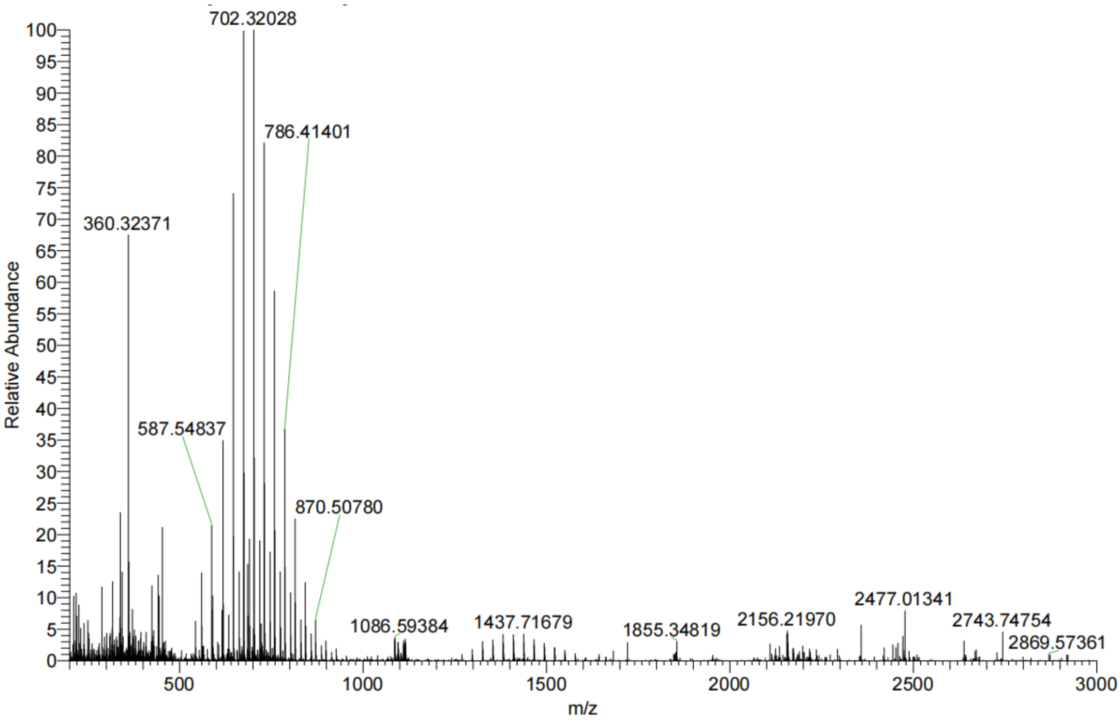

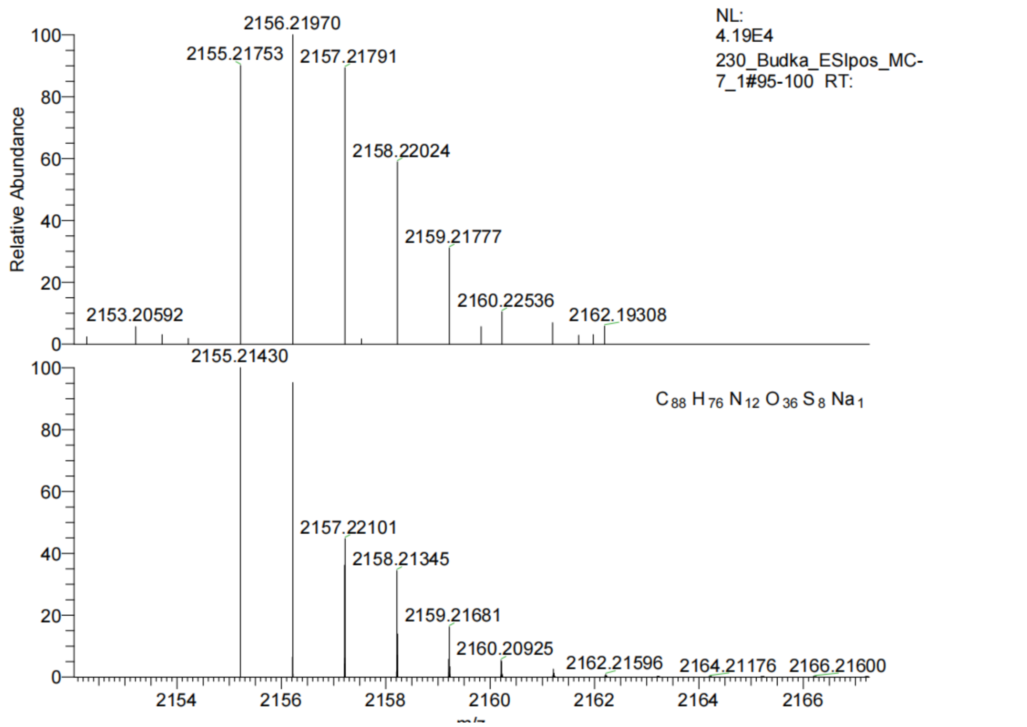

Fig. S51: HRMS of compound **7** calc $[C_{88}H_{76}N_{12}O_{36}S_8+Na]^+$ 2155.2143; found *m/z* 2155.2175 $[M+Na]^+$.

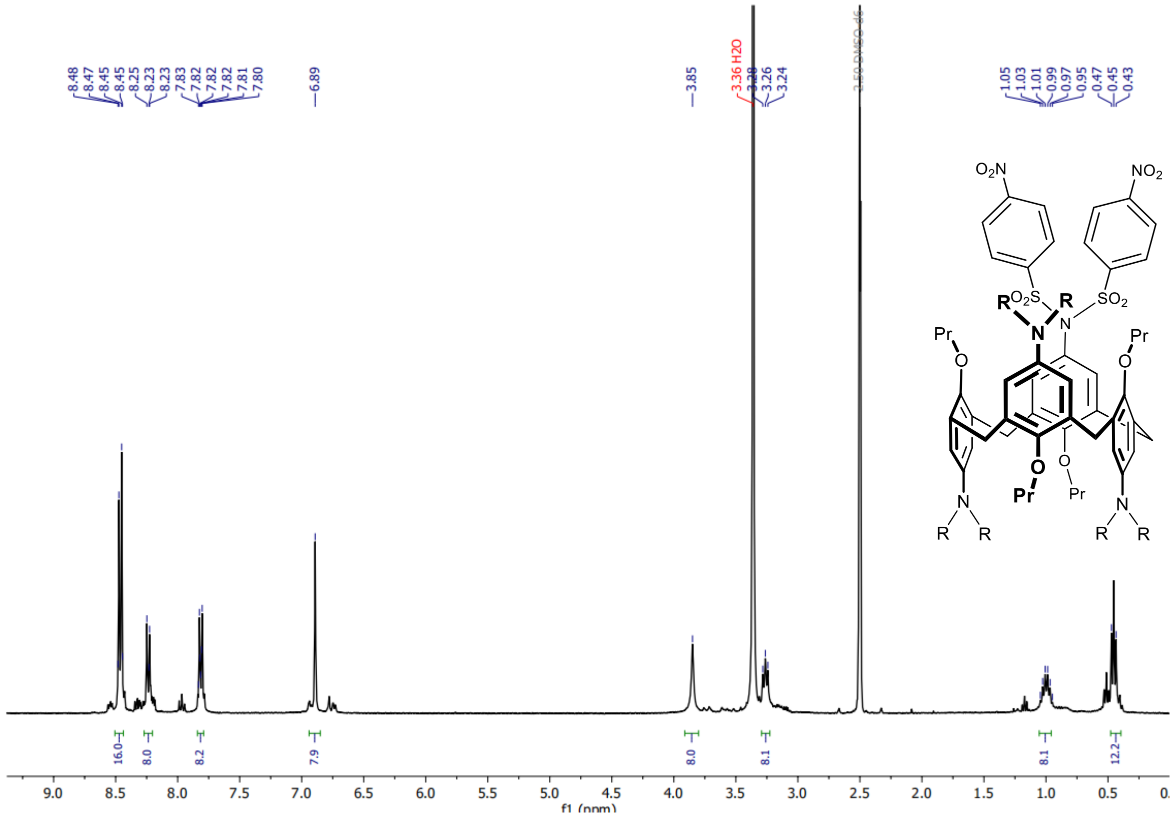

Fig. S52: ^{1}H NMR of compound **8** (DMSO-d_6, 400 MHz).

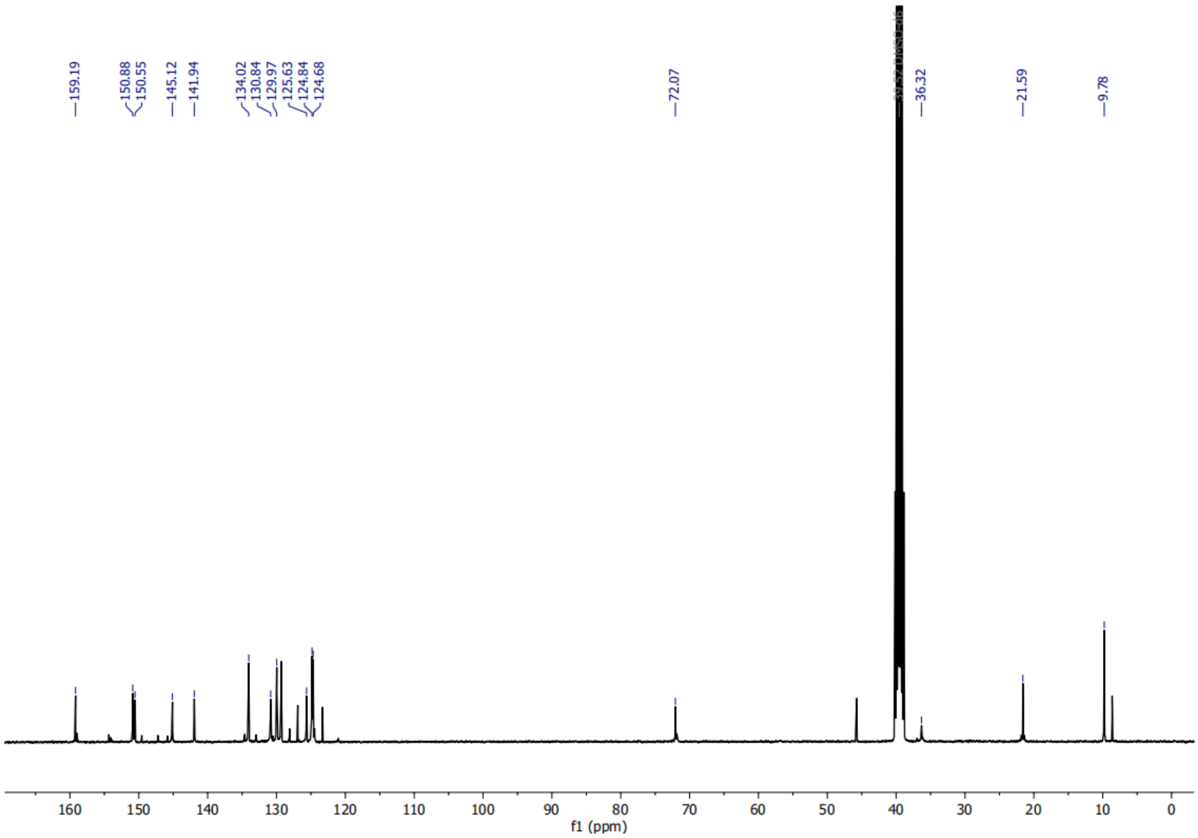

Fig. S53: $^{13}C\{^{1}H\}$ NMR of compound **8** (DMSO-d_6, 100 MHz).

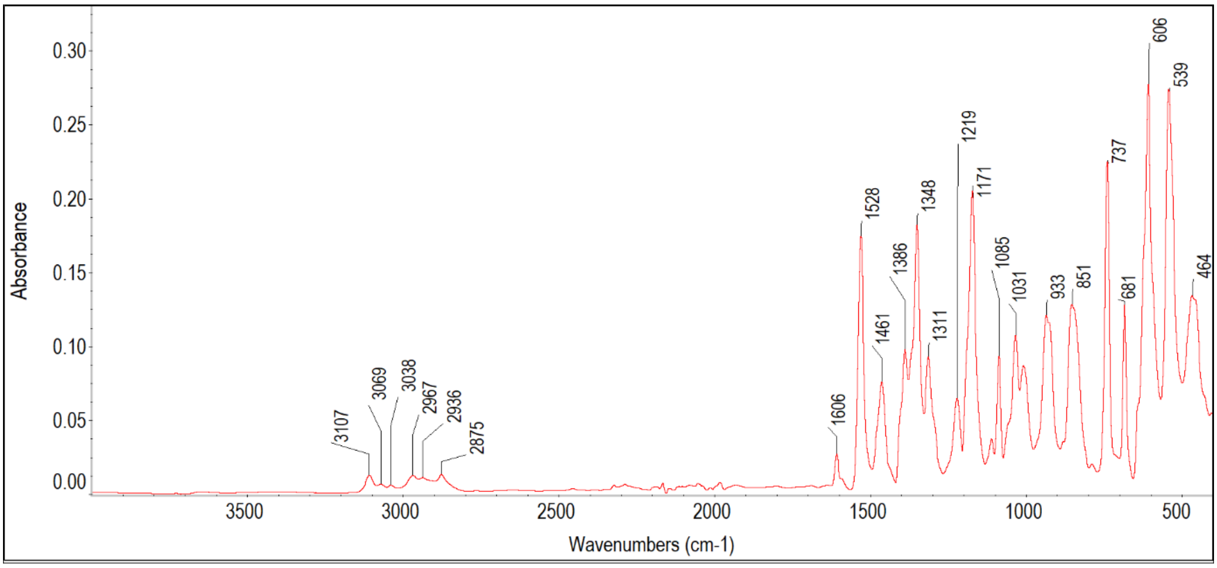

Fig. S54: IR spectrum of compound **8** (ATR).

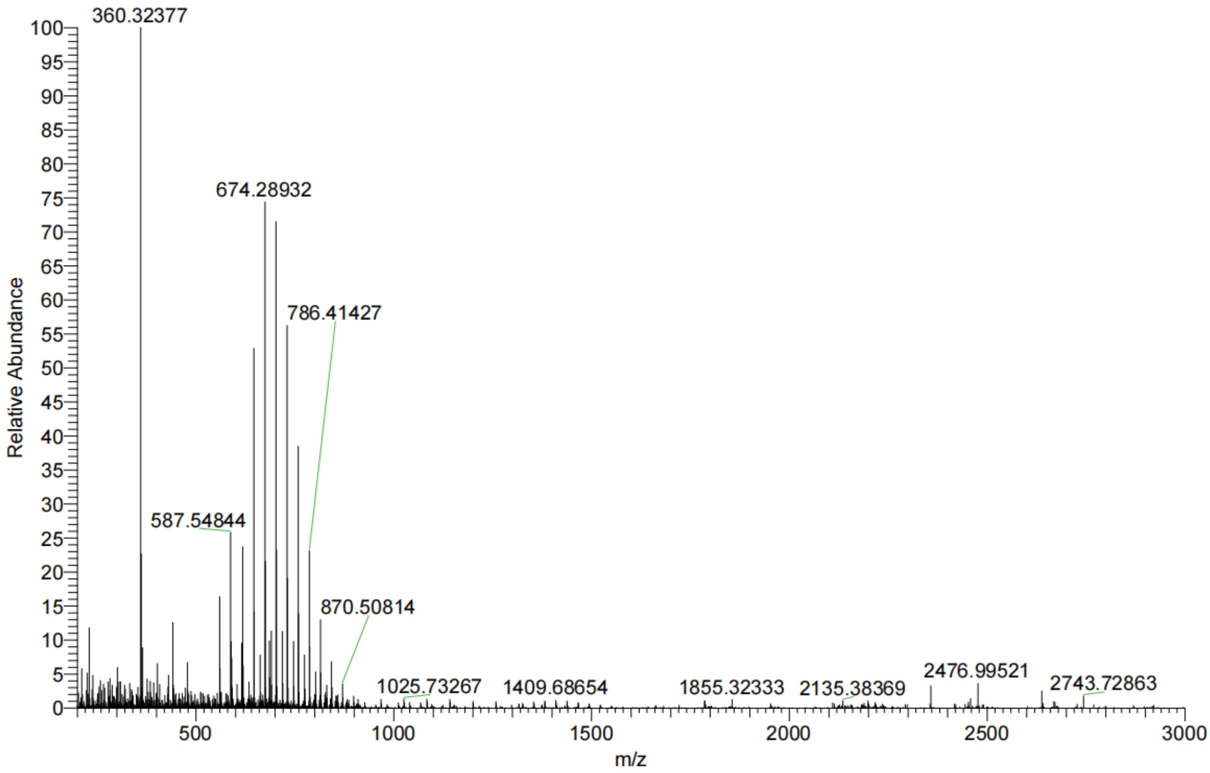

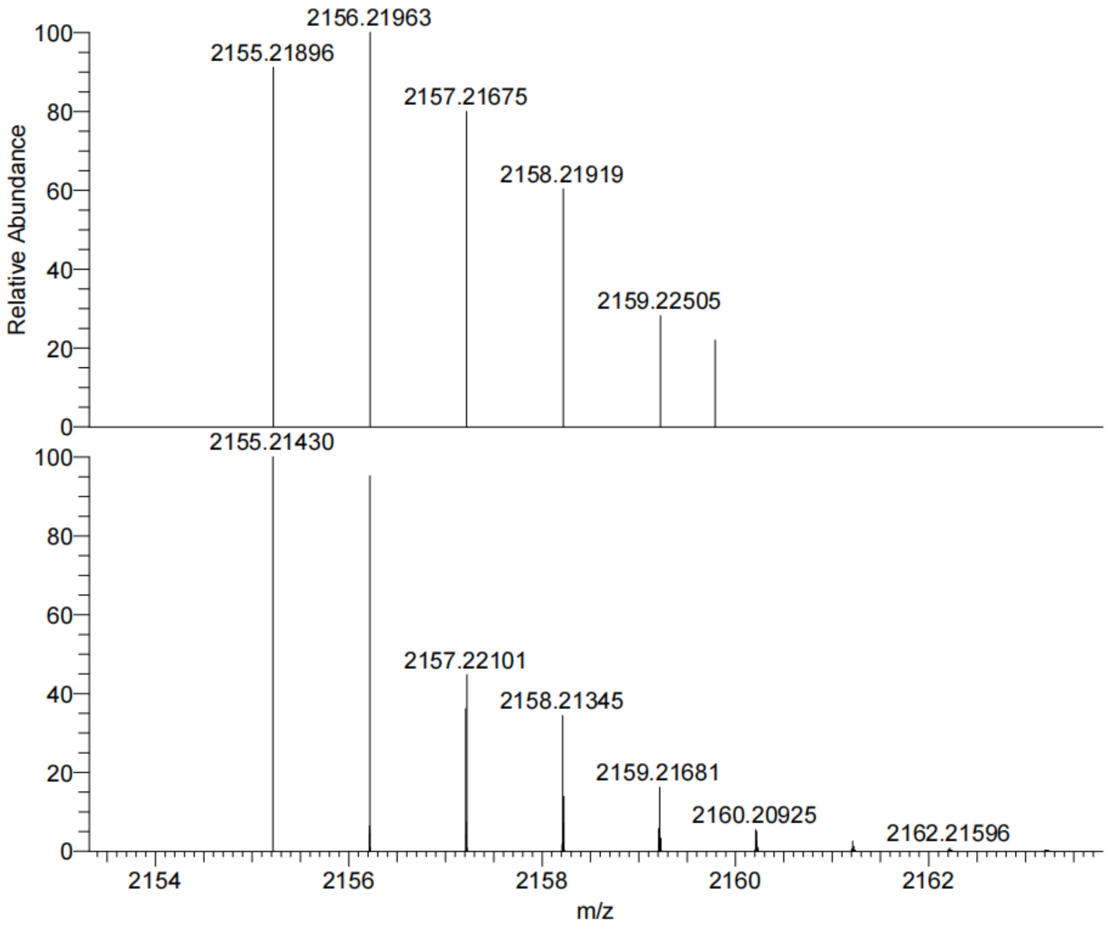

Fig. S55: HRMS of compound **8** calc $[C_{88}H_{76}N_{12}O_{36}S_8+Na]^+$ 2155.2143; found *m/z* 2155.2190 $[M+Na]^+$.

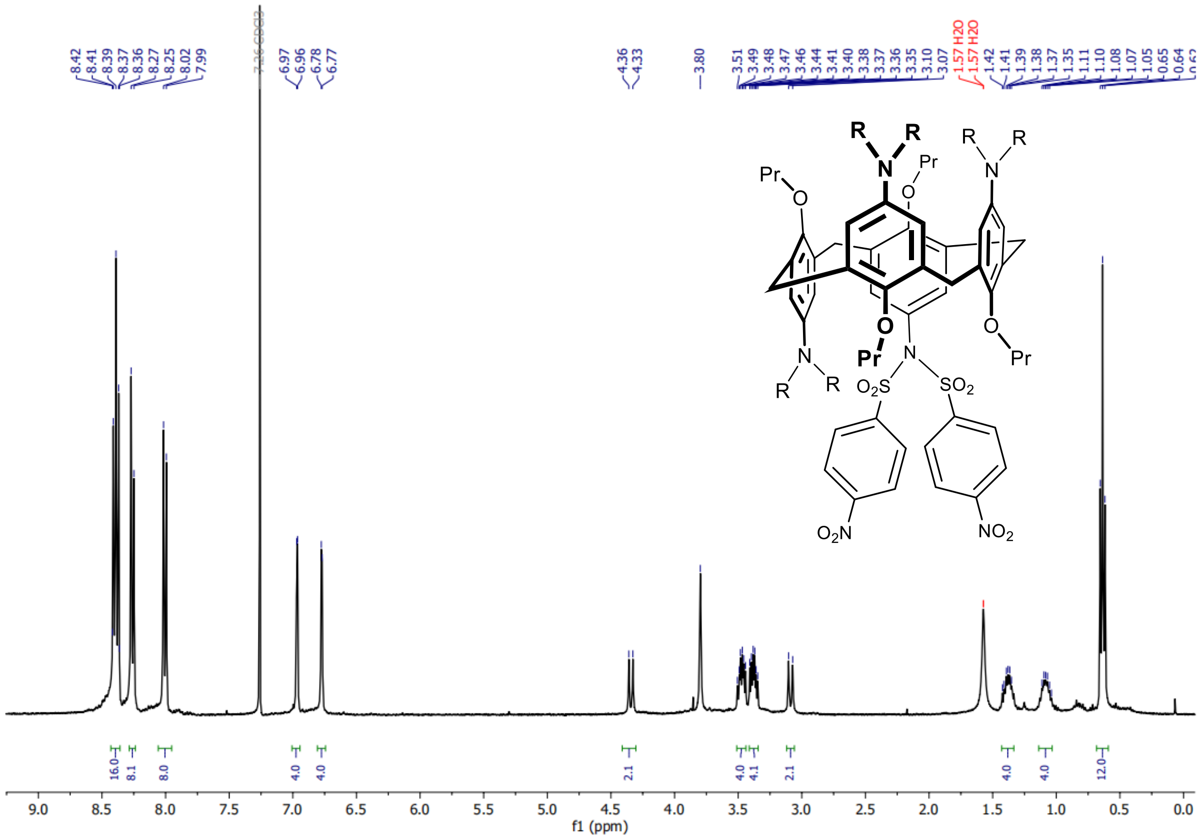

Fig. S56: ^{1}H NMR of compound **9** ($CDCl_3$, 400 MHz).

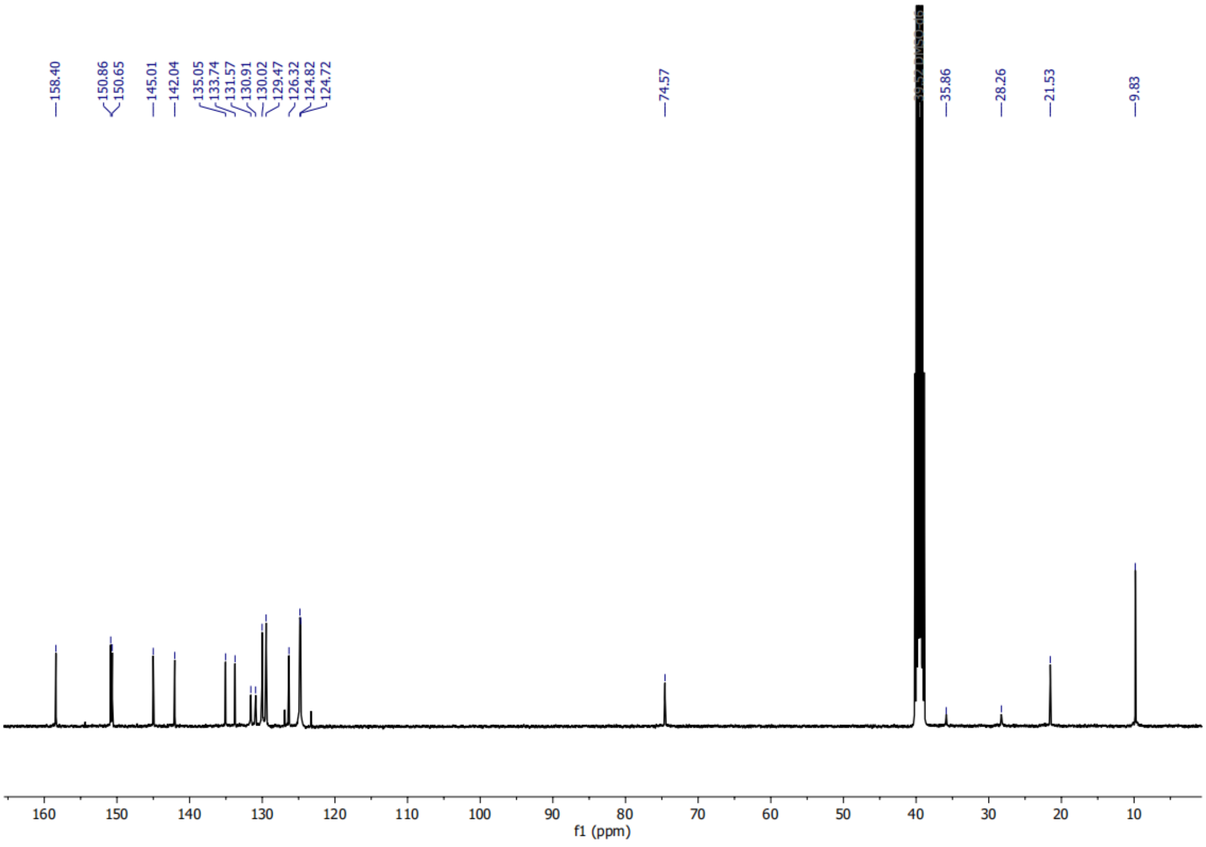

Fig. S57: $^{13}C\{^{1}H\}$ NMR of compound **9** (DMSO-d_6, 100 MHz).

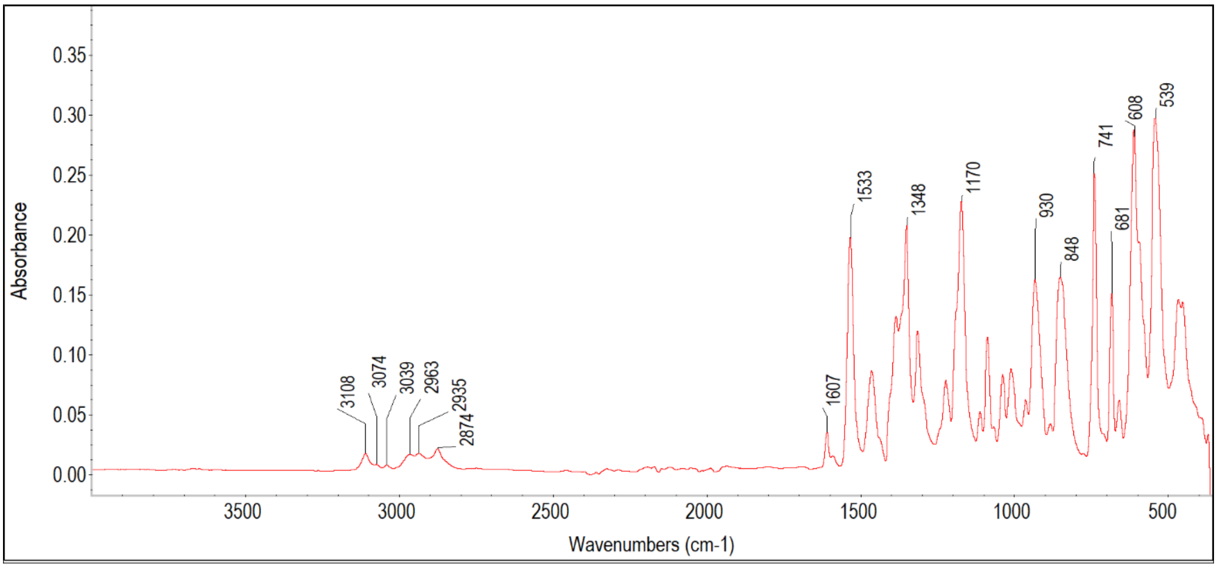

Fig. S58: IR spectrum of compound **9** (ATR).

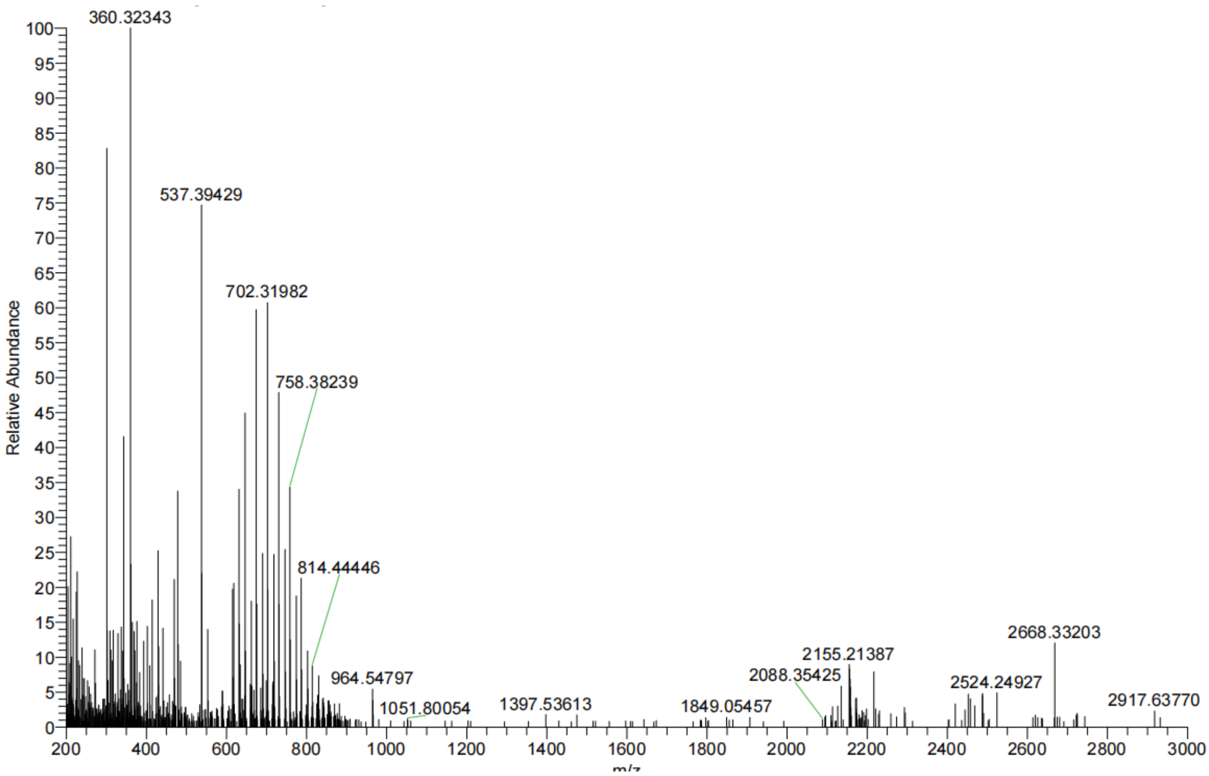

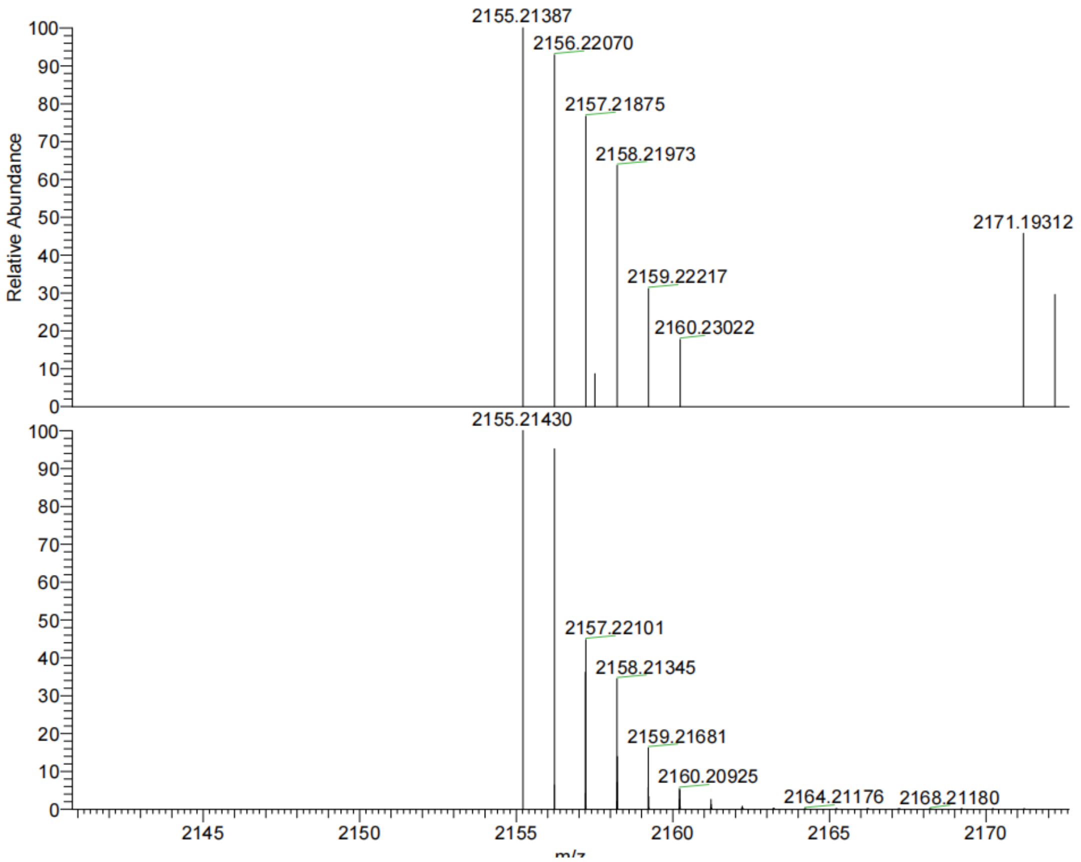

Fig. S59: HRMS of compound **9** calc $[C_{88}H_{76}N_{12}O_{36}S_8+Na]^+$ 2155.2143; found *m/z* 2155.2139 $[M+Na]^+$.

2. Spectral characterisation of obtained sulfonamides

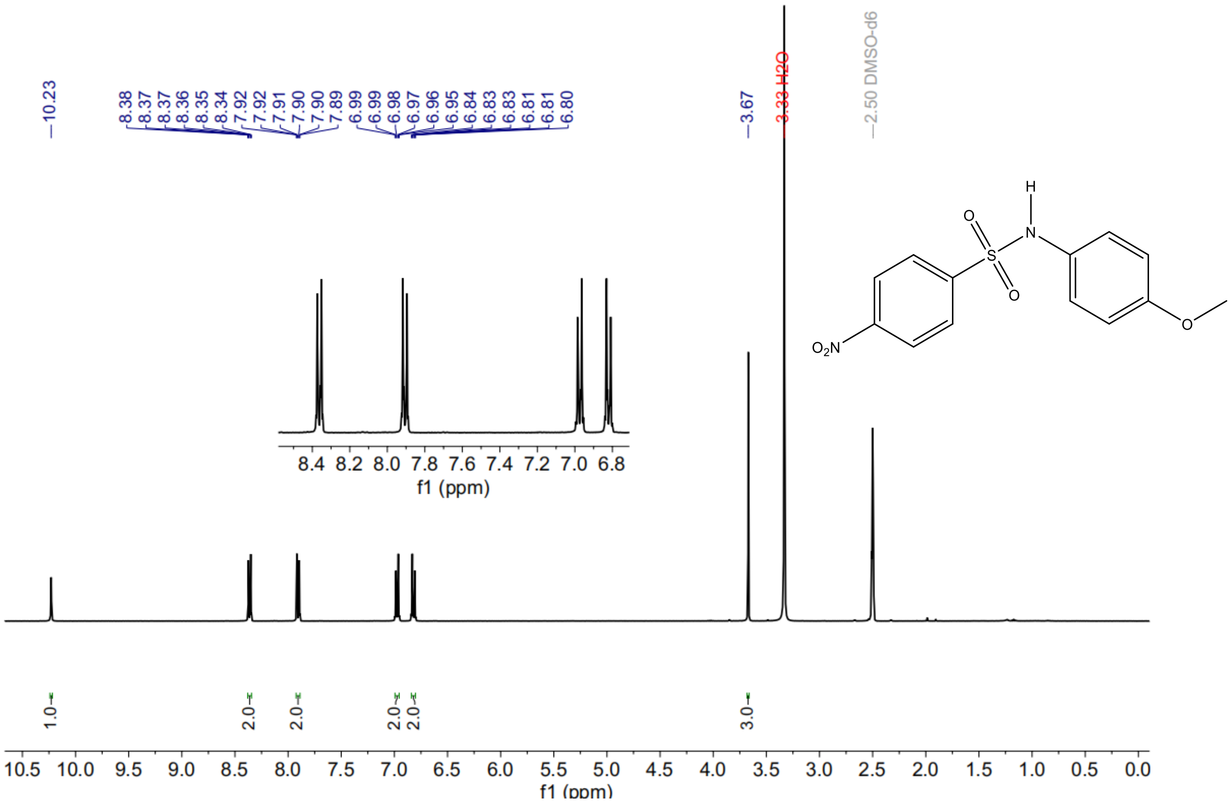

Fig. S60: ^{1}H NMR of compound **2Aa** (DMSO-d_6, 400 MHz).

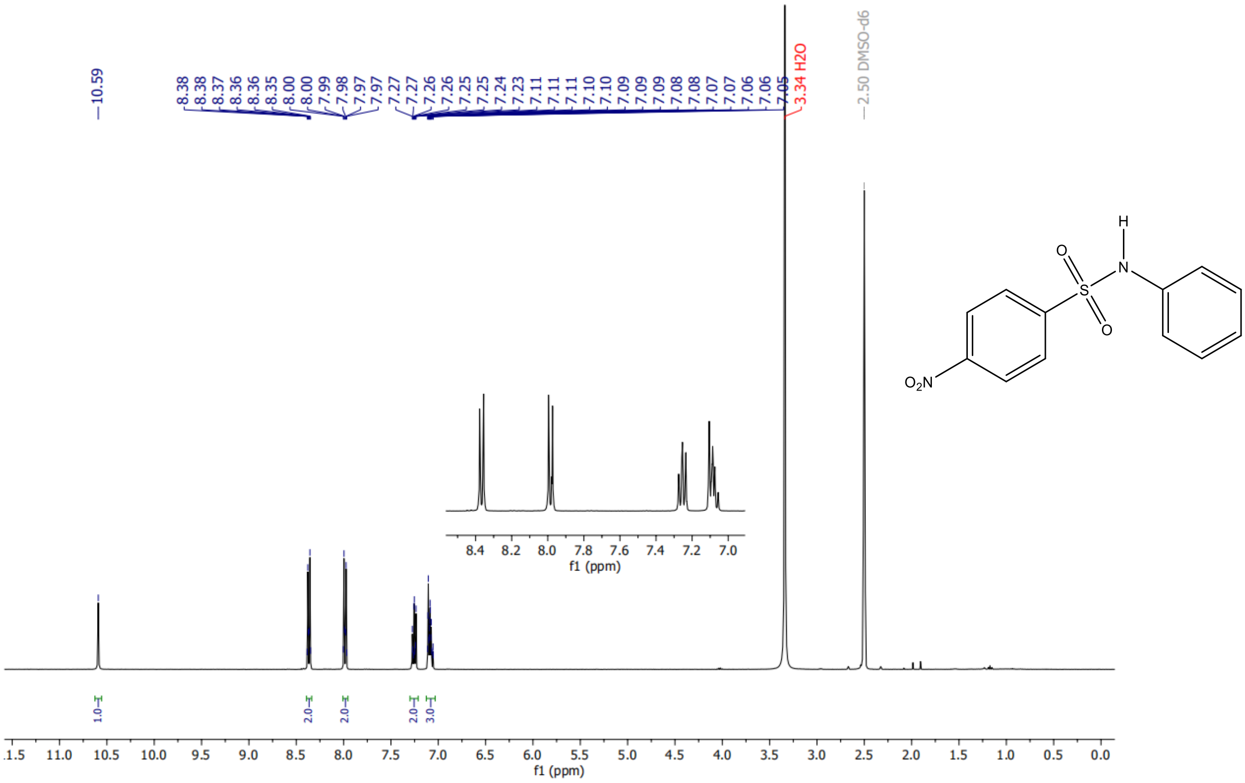

Fig. S61: ^{1}H NMR of compound **2Ab** (DMSO-d_6, 400 MHz).

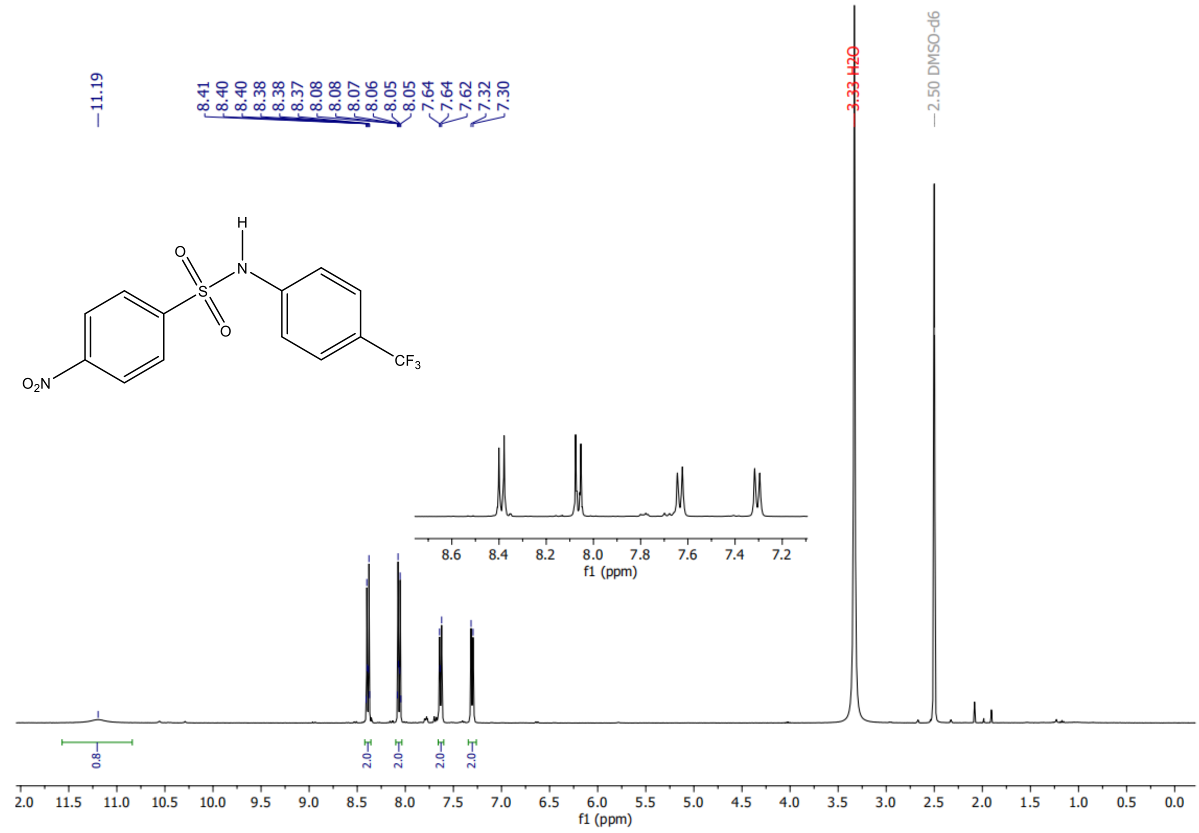

Fig. S62: ^{1}H NMR of compound **2Ac** (DMSO-d_6, 400 MHz).

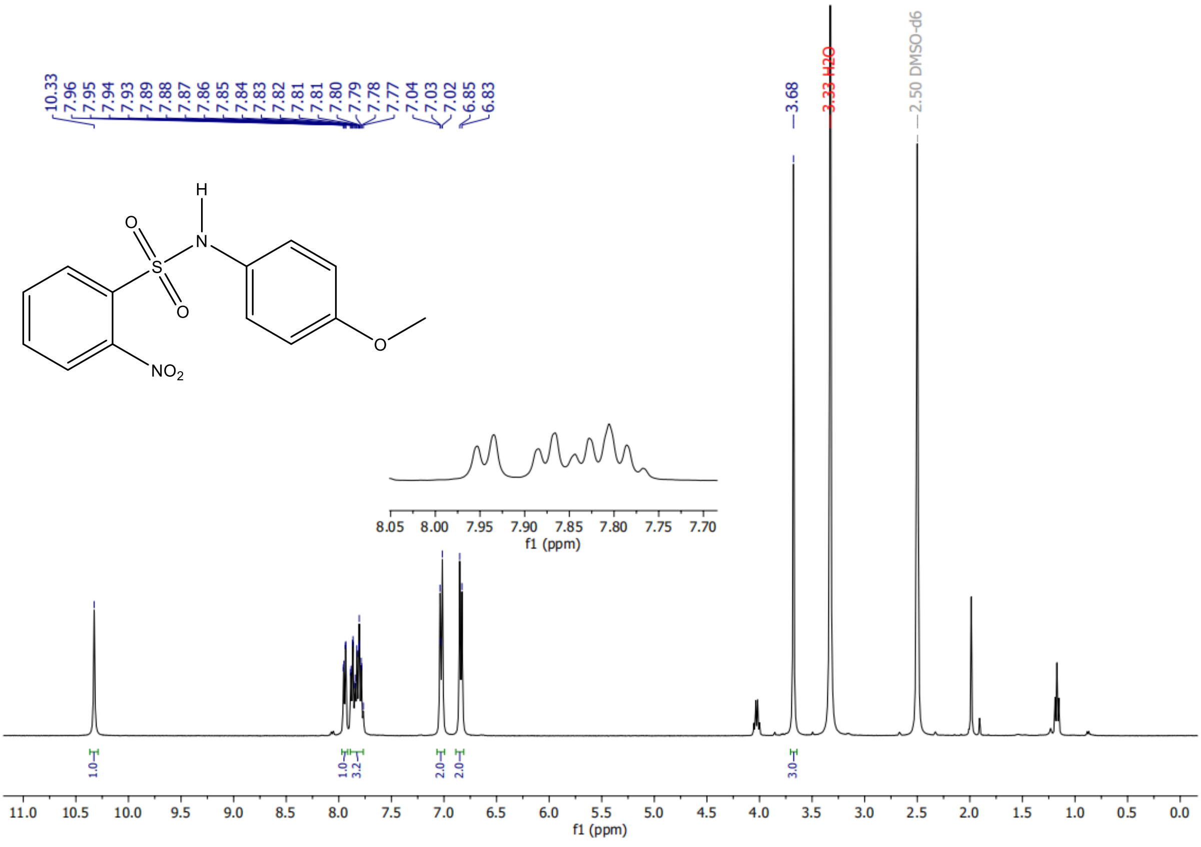

Fig. S63: ^{1}H NMR of compound **3Aa** (DMSO-d_6, 400 MHz).

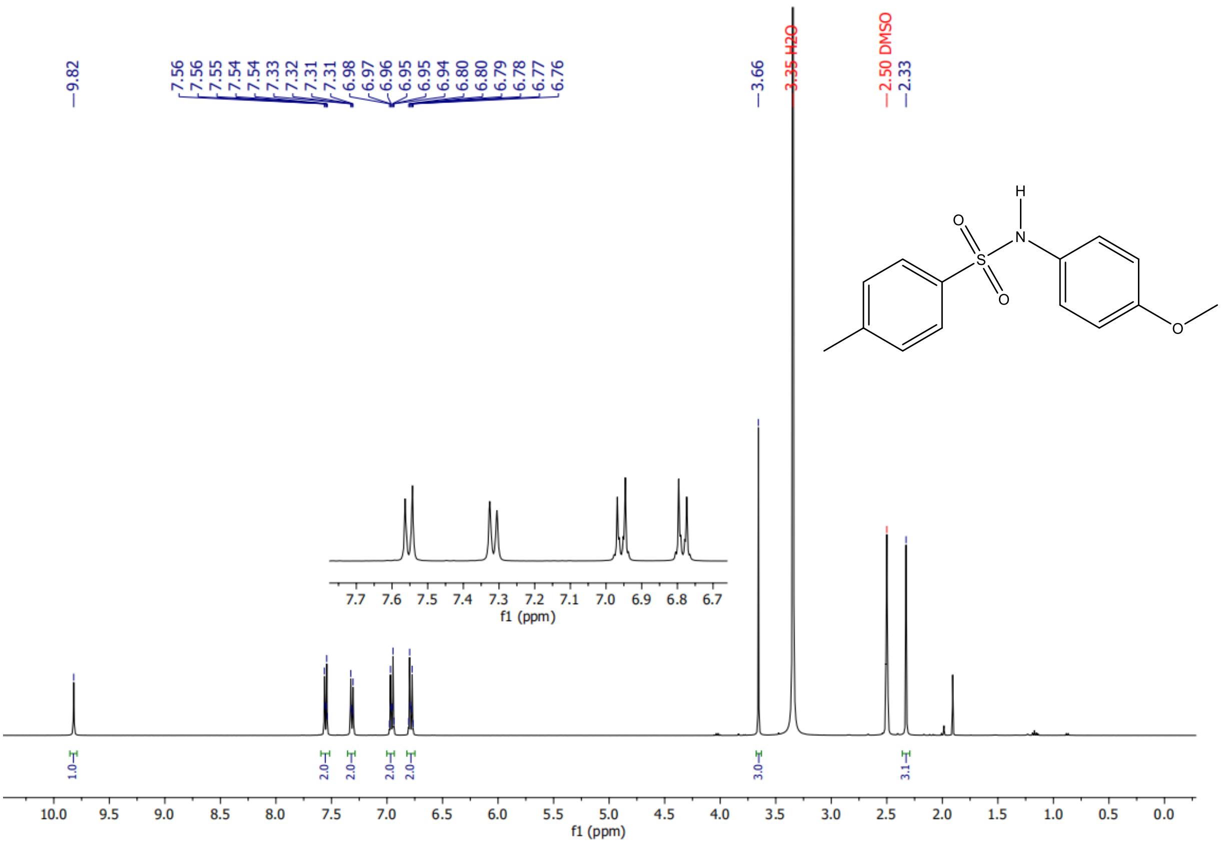

Fig. S64: ^{1}H NMR of compound **3Ab** (DMSO-d_6, 400 MHz).

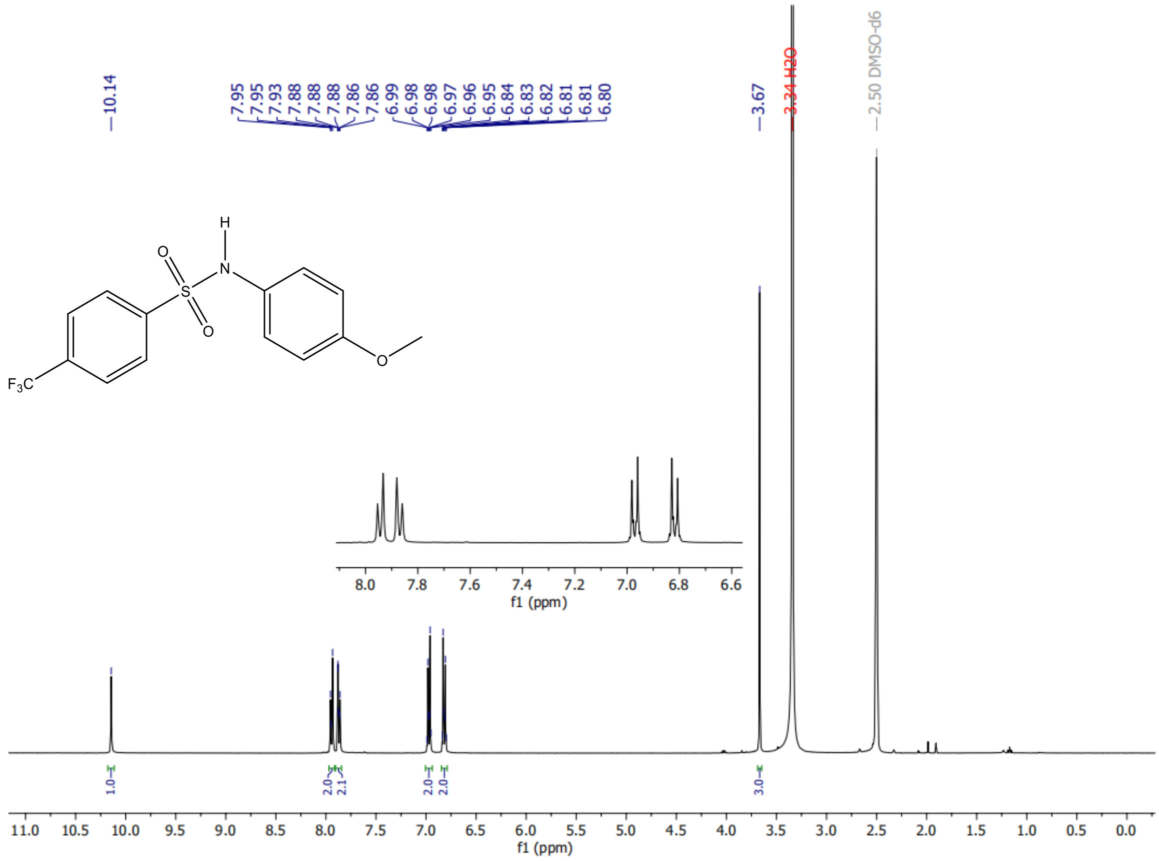

Fig. S65: ^{1}H NMR of compound **3Ac** (DMSO-d_6, 400 MHz).

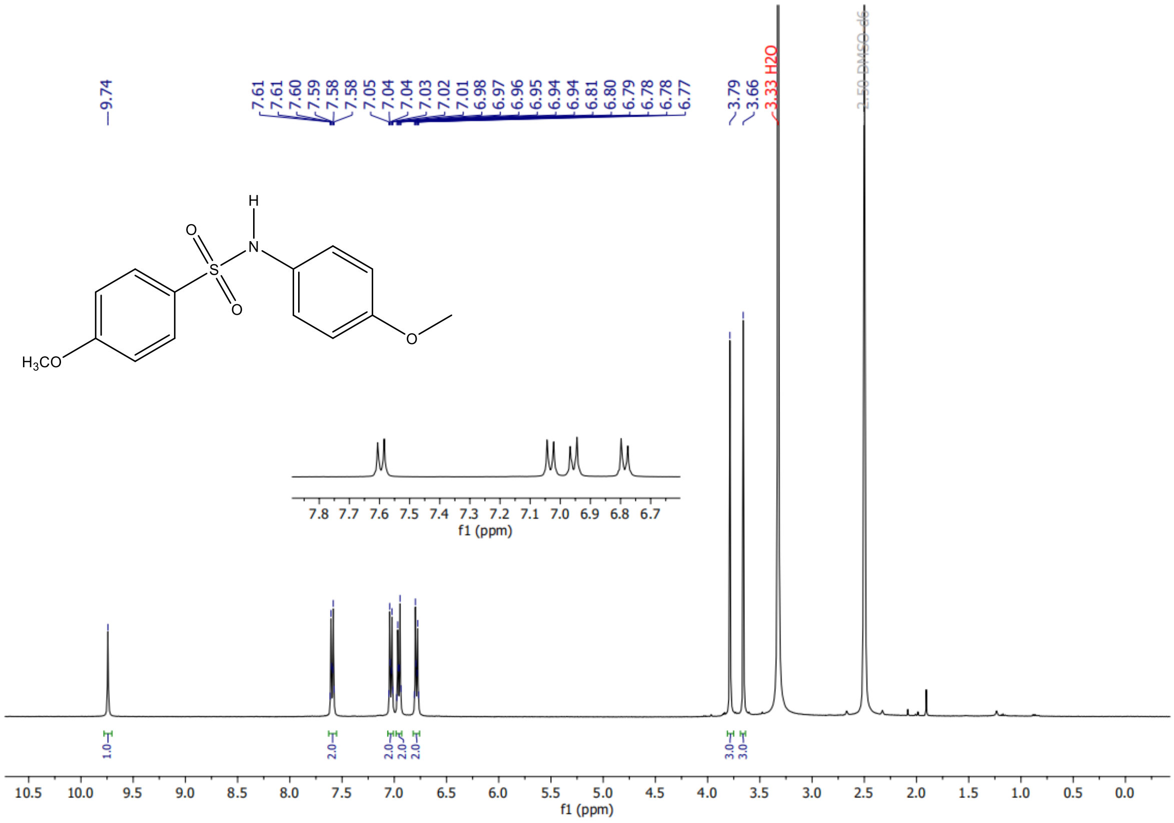

Fig. S66: ^{1}H NMR of compound **3Ad** (DMSO-d_6, 400 MHz).

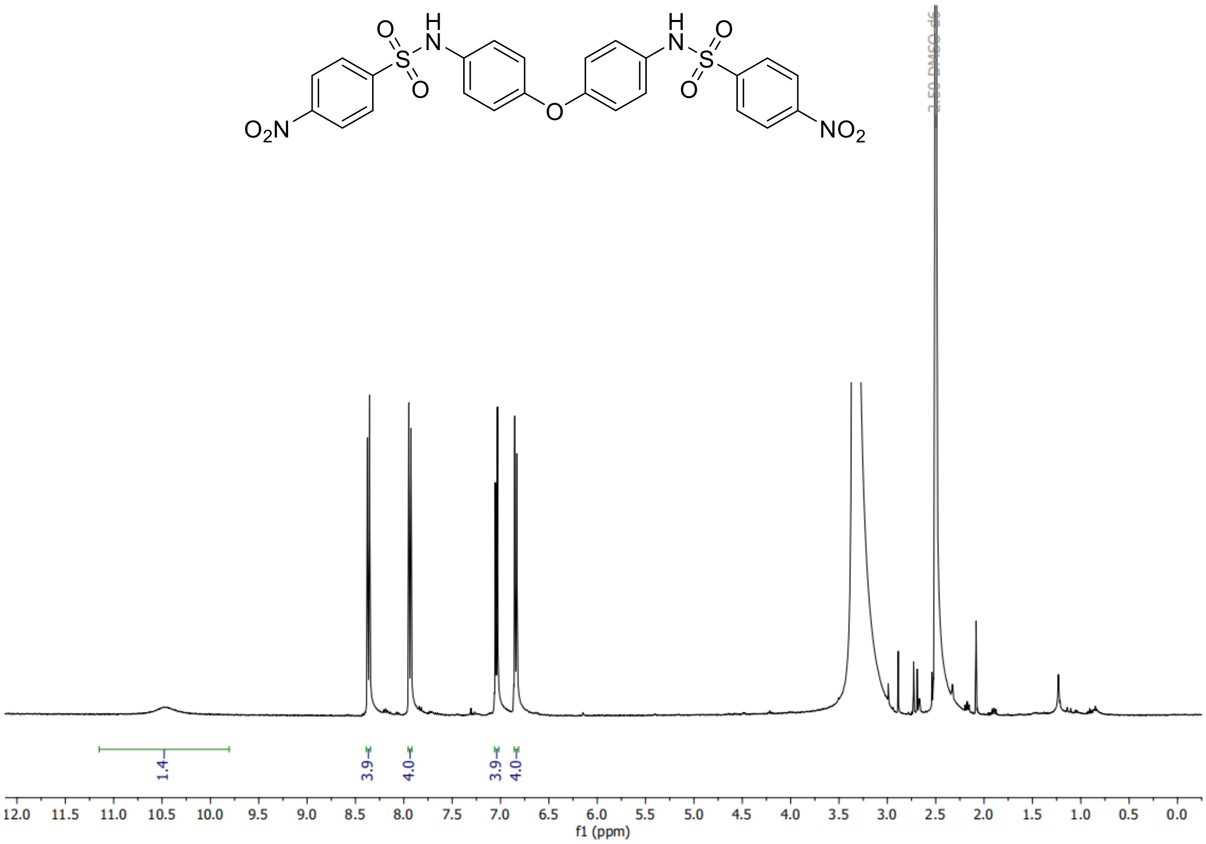

Fig. S67: ^{1}H NMR of compound **6A** (DMSO-d_6, 400 MHz).

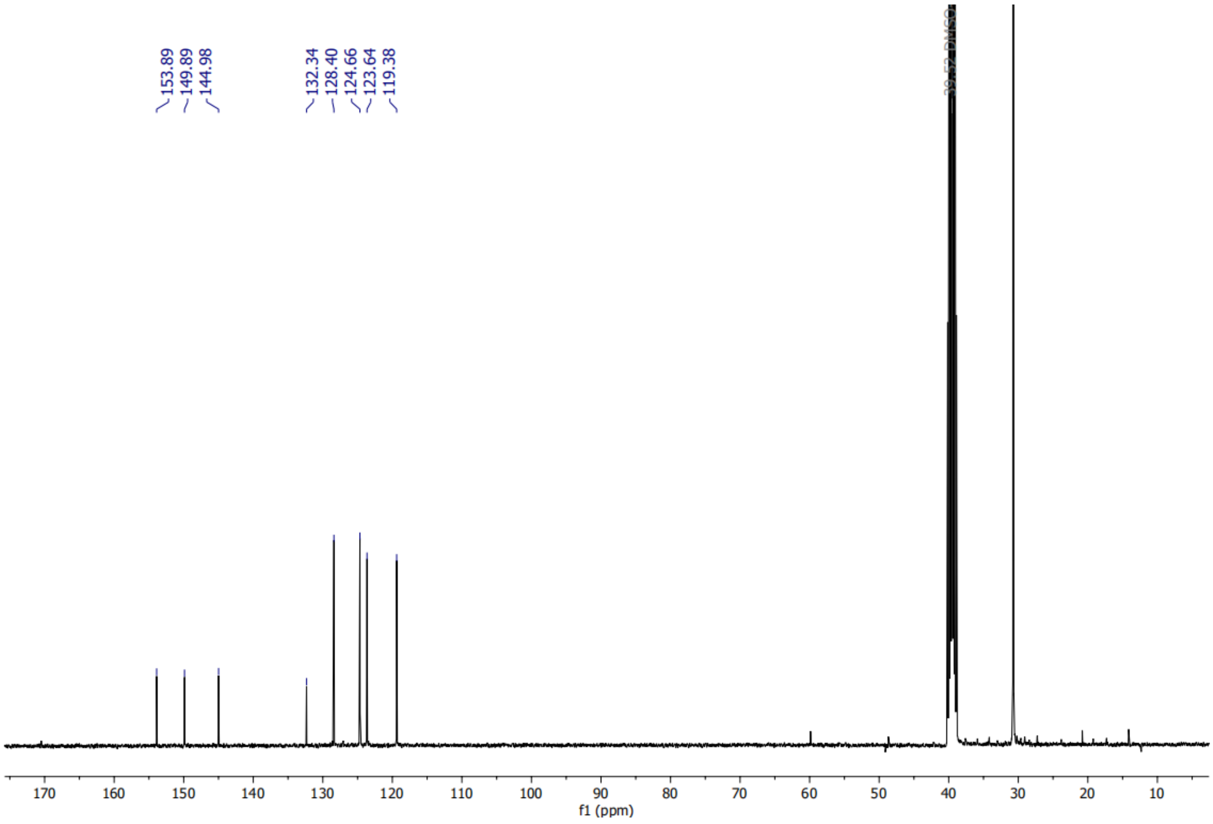

Fig. S68: $^{13}C\{^{1}H\}$ NMR of compound **6A** (DMSO-d_6, 100 MHz).

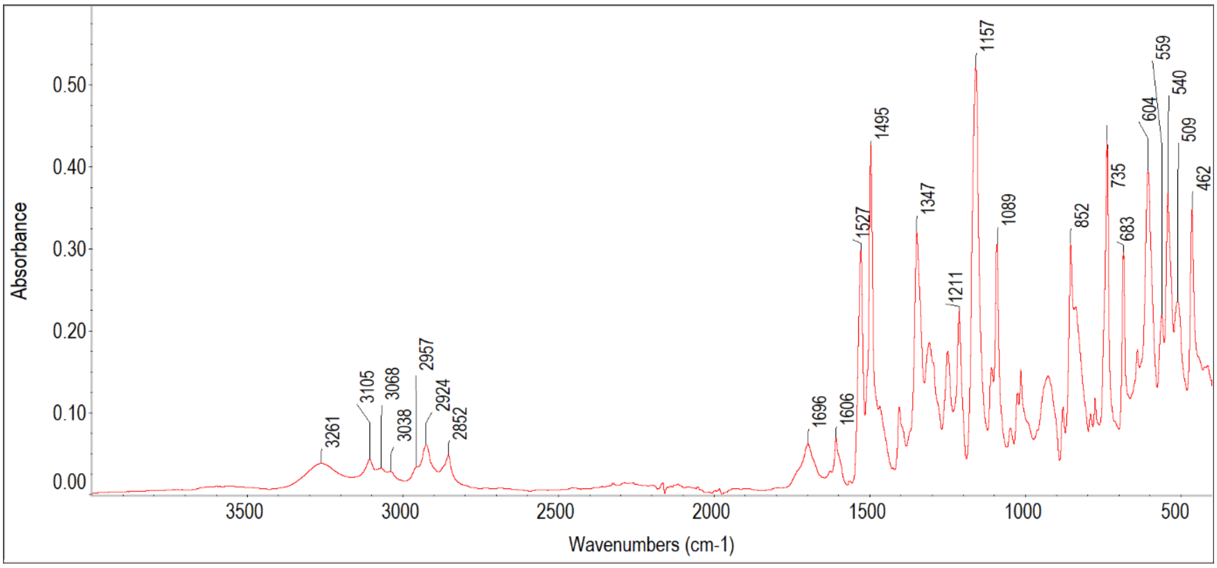

Fig. S69: IR spectrum of compound **6A** (ATR).

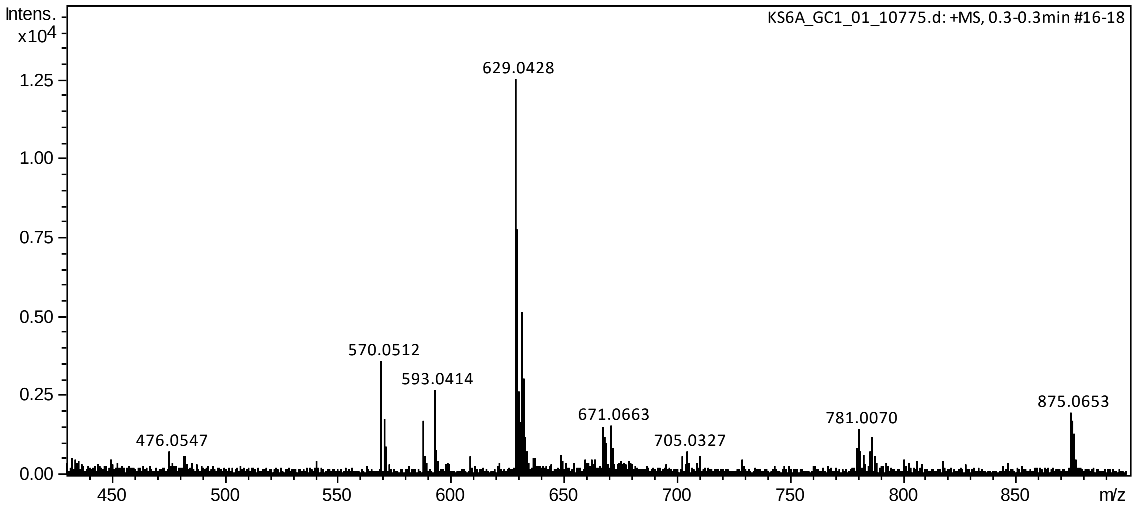

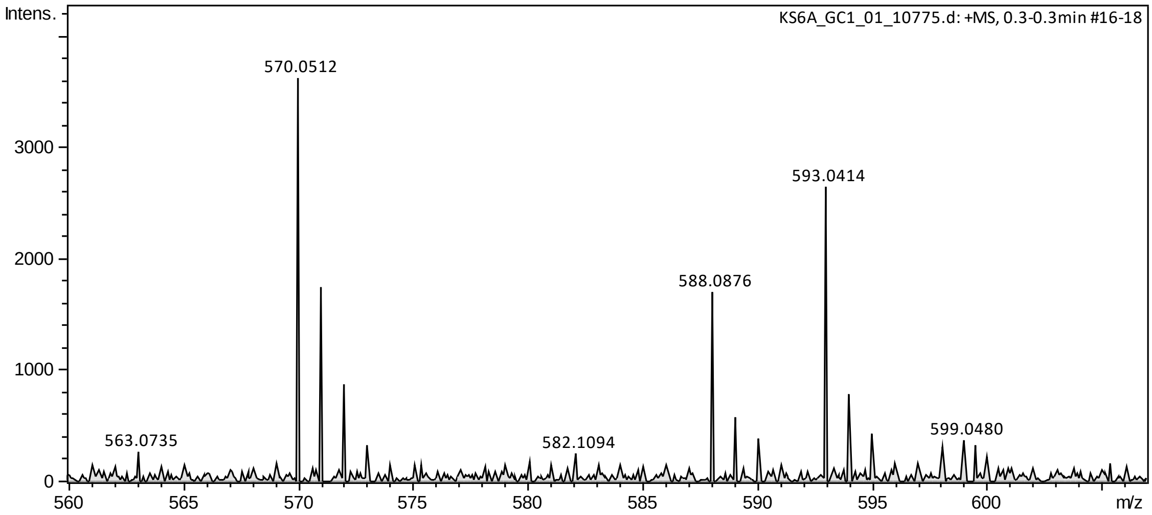

Fig. S70: HRMS of compound **6A** calc $[C_{24}H_{18}N_4O_9S_2]^+$ 570.0510; found *m/z* 570.0512 $[M]^+$, and calc $[C_{24}H_{18}N_4O_9S_2+Na]^+$ 593.0407; found *m/z* 593.0414 $[M+Na]^+$.

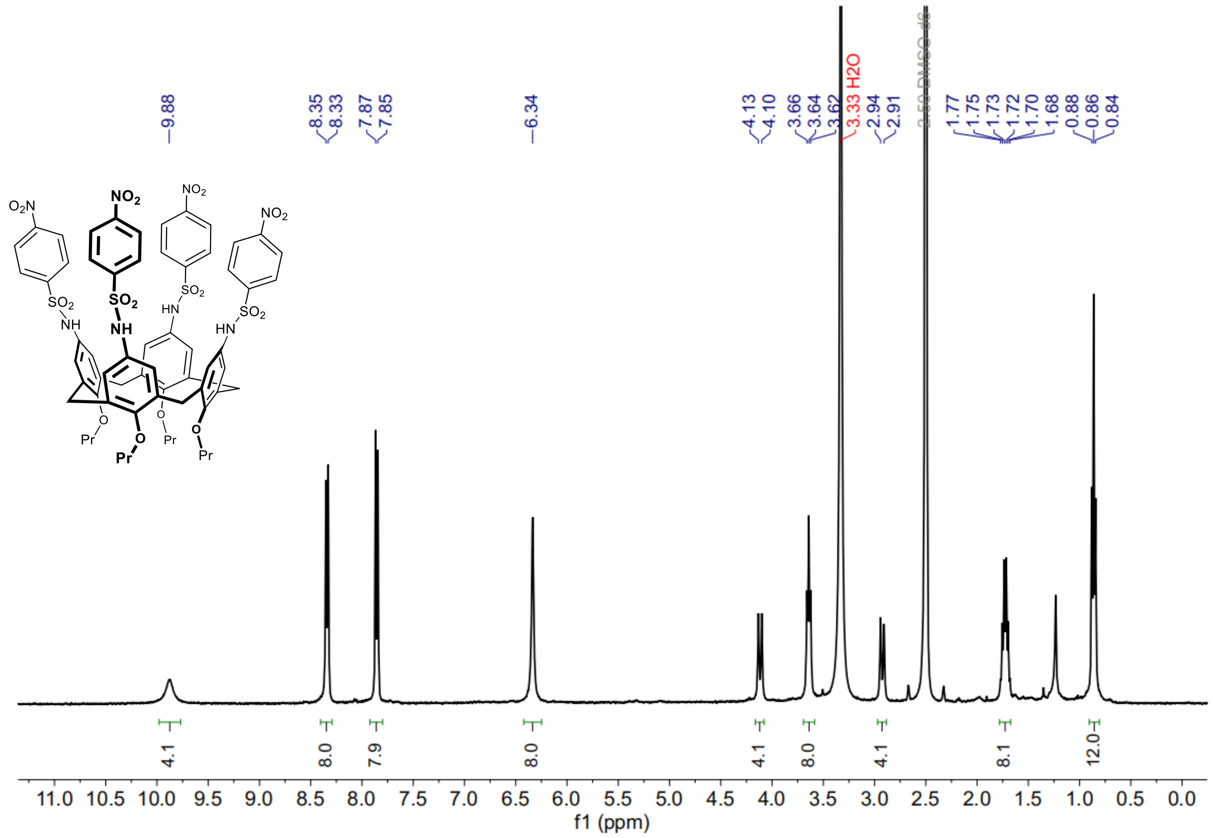

Fig. S71: ^{1}H NMR of compound **7A** (DMSO-d_6, 400 MHz).

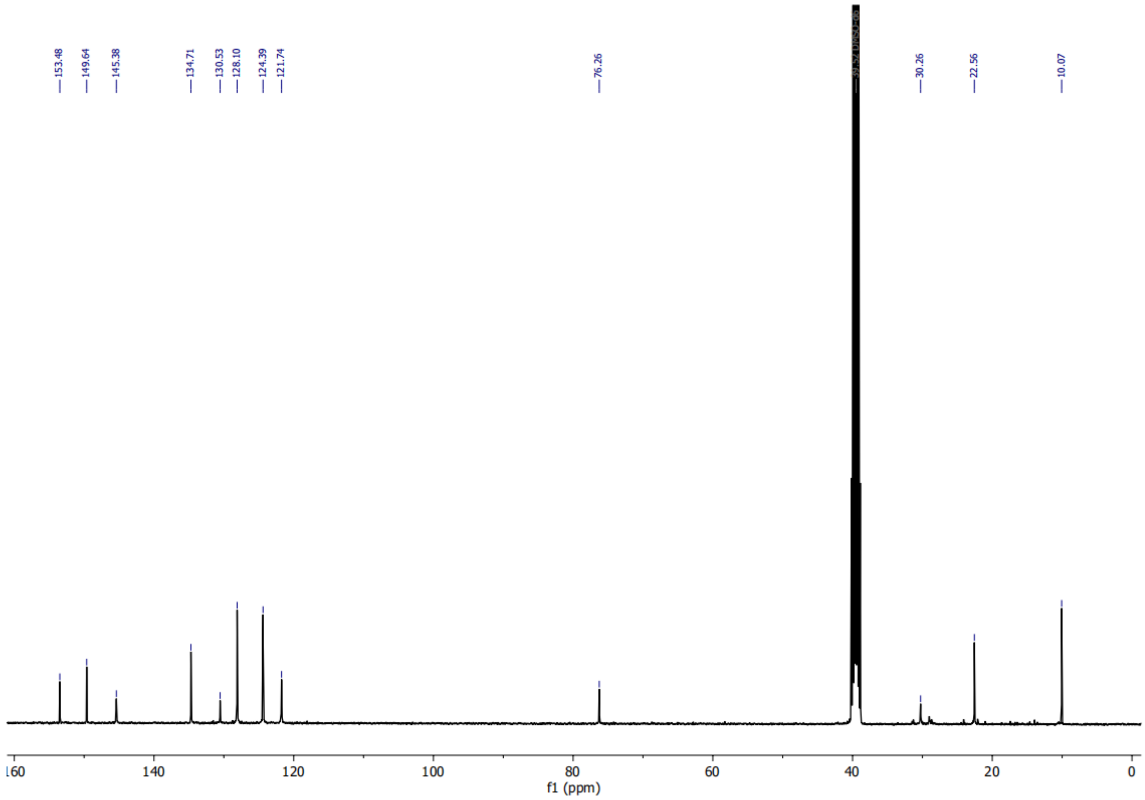

Fig. S72: $^{13}C\{^{1}H\}$ NMR of compound **7A** (DMSO-d_6, 100 MHz).

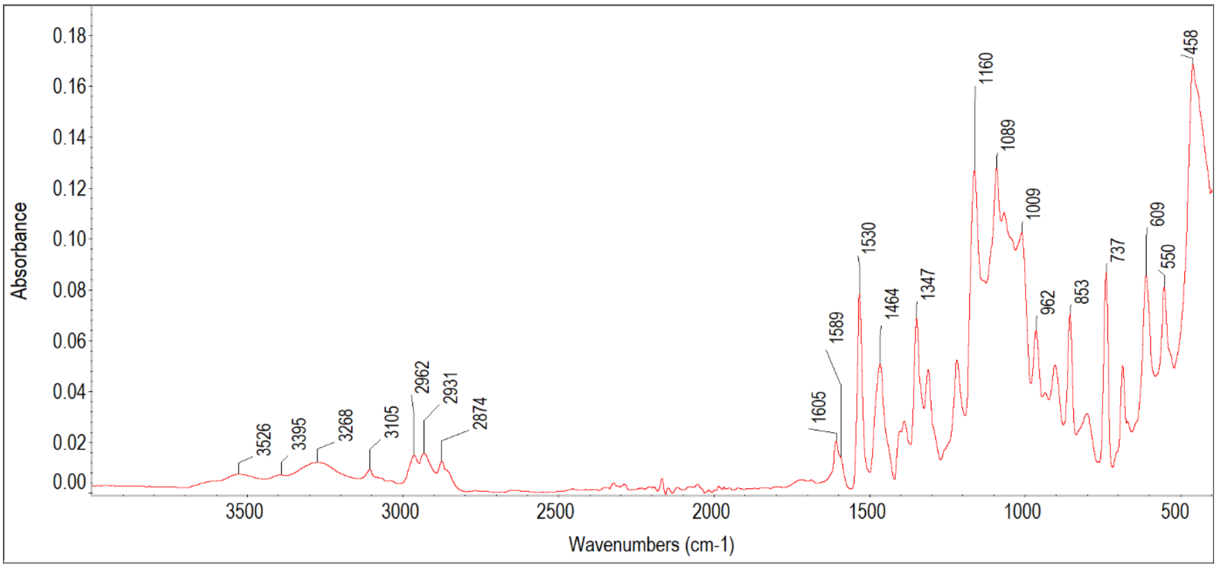

Fig. S73: IR spectrum of compound **7A** (ATR).

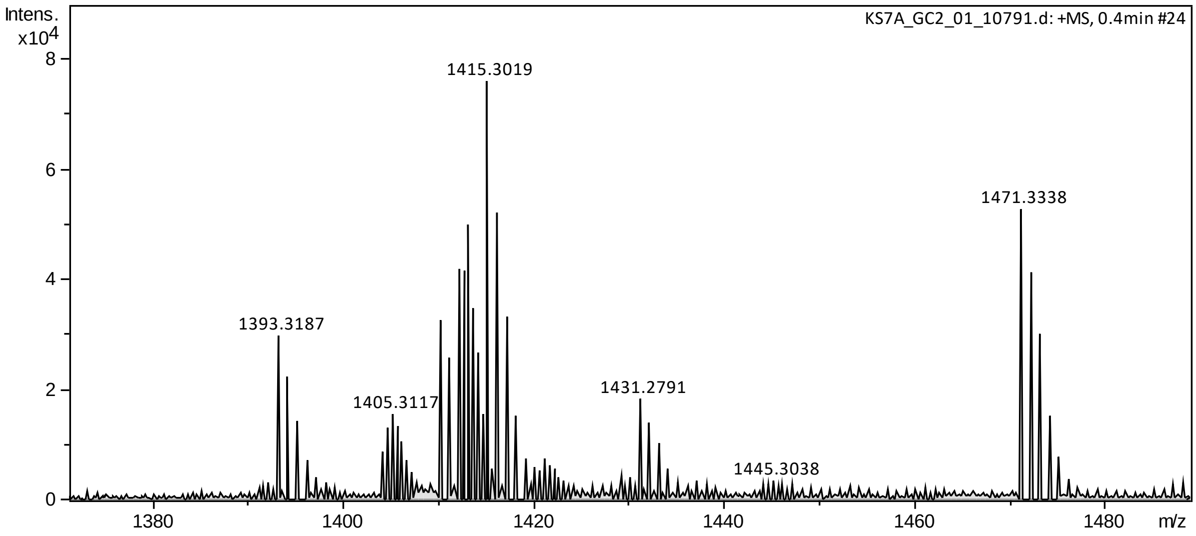

Fig. S74: HRMS of compound **7A** calc $[C_{64}H_{65}N_8O_{20}S_4]^+$ 1393.3193; found *m/z* 1393.3187 $[M+H]^+$, and calc $[C_{64}H_{64}N_8O_{20}S_4+Na]^+$ 1415.3012; found *m/z* 1415.3019 $[M+Na]^+$.

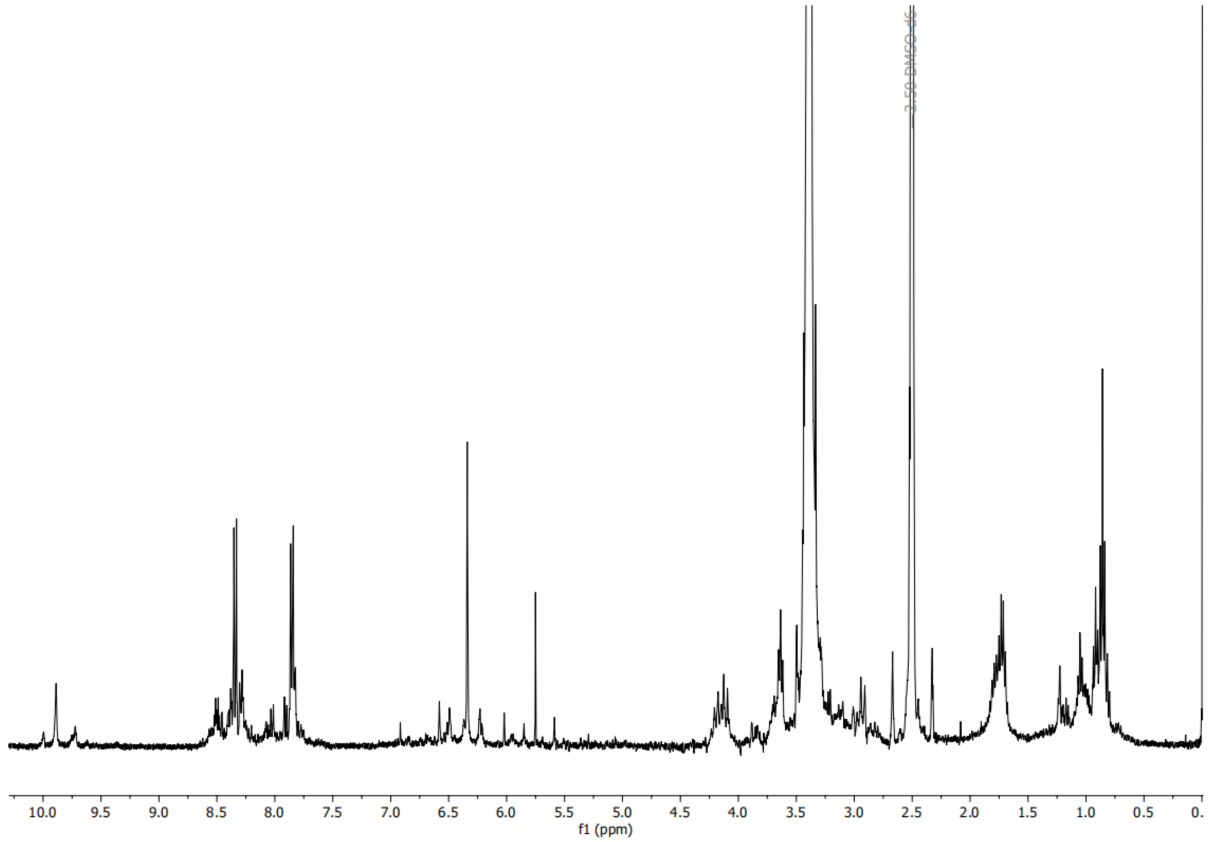

Fig. S75: ^{1}H NMR spectra of a crude mixture of **7A** in DMSO-d_6 after the attempt for its preparation by traditional synthesis.

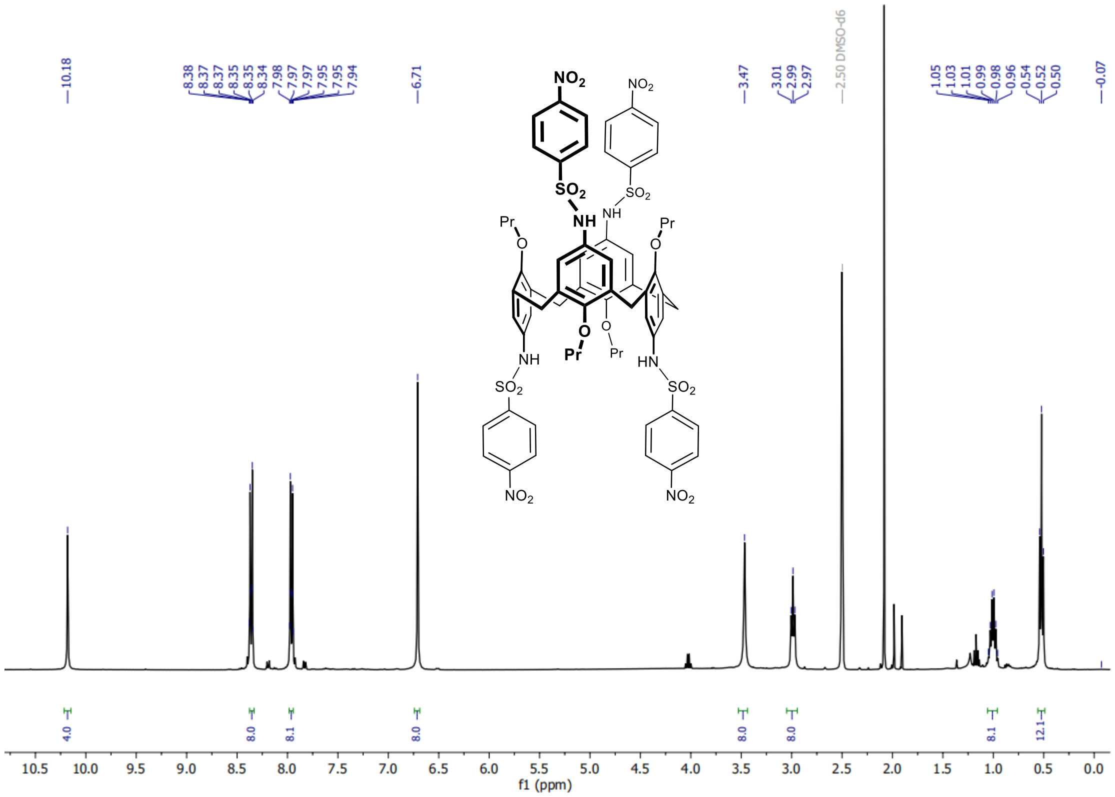

Fig S76: ^{1}H NMR of compound **8A** (DMSO-d_6, 400 MHz).

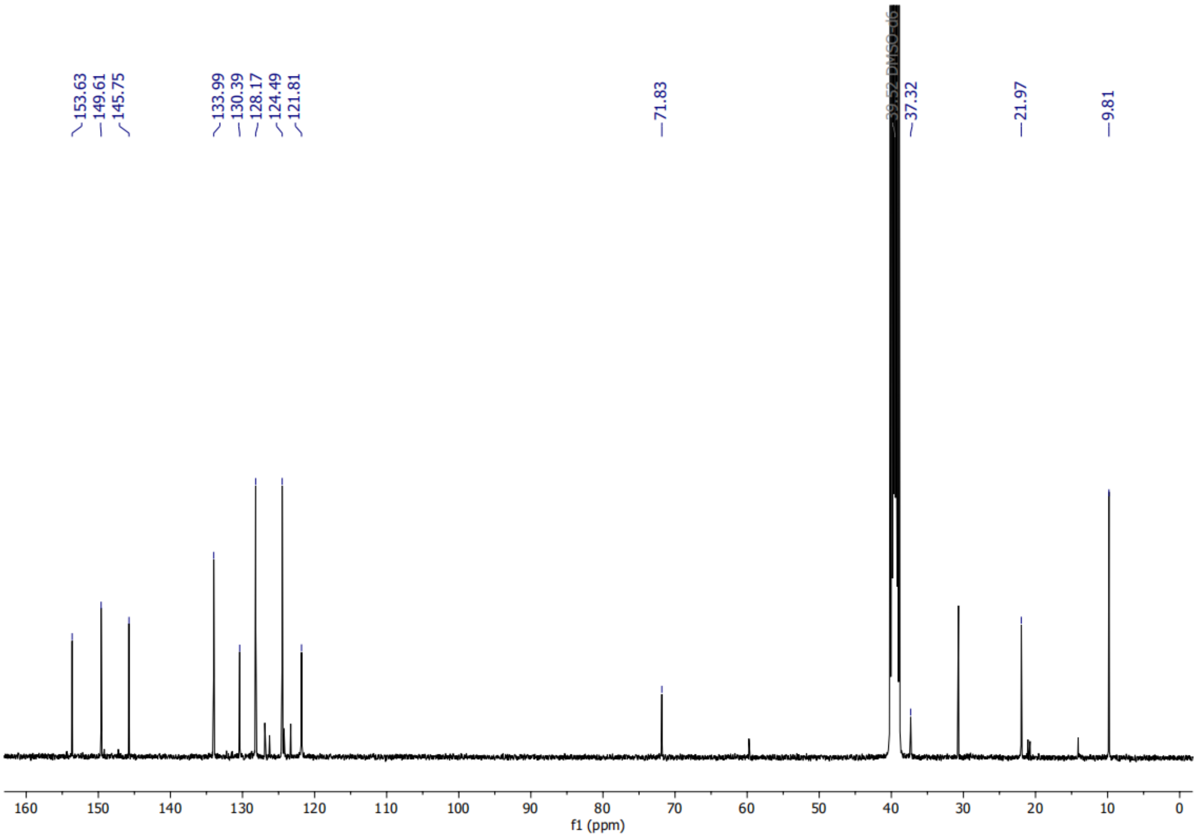

Fig. S77: ^{13}C{^{1}H} NMR of compound **8A** (DMSO-d_6, 100 MHz).

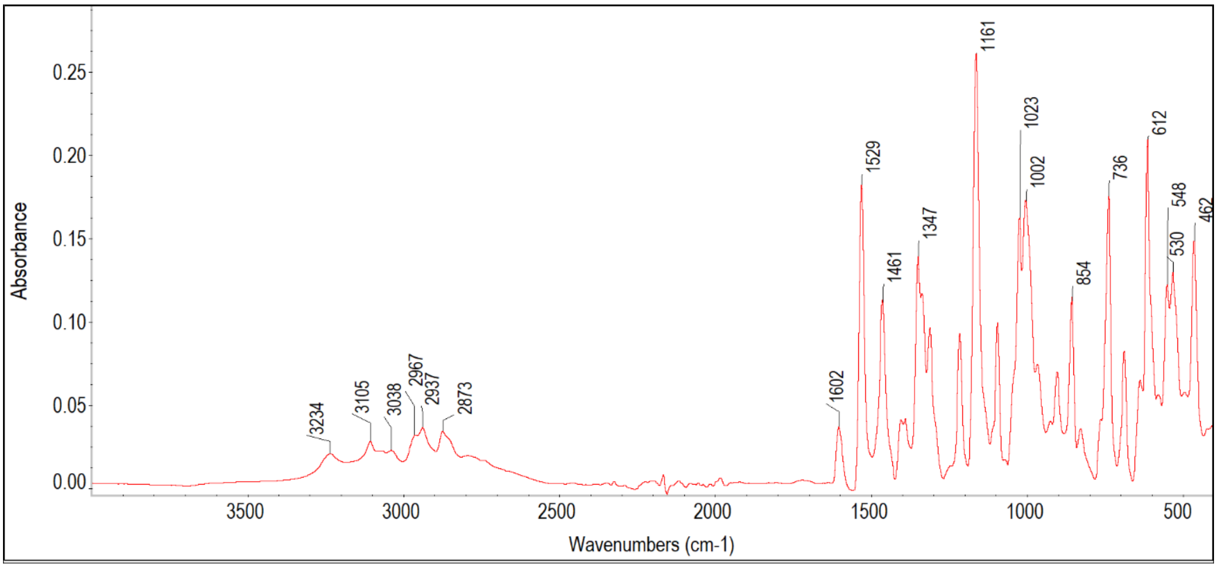

Fig. S78: IR spectrum of compound **8A** (ATR).

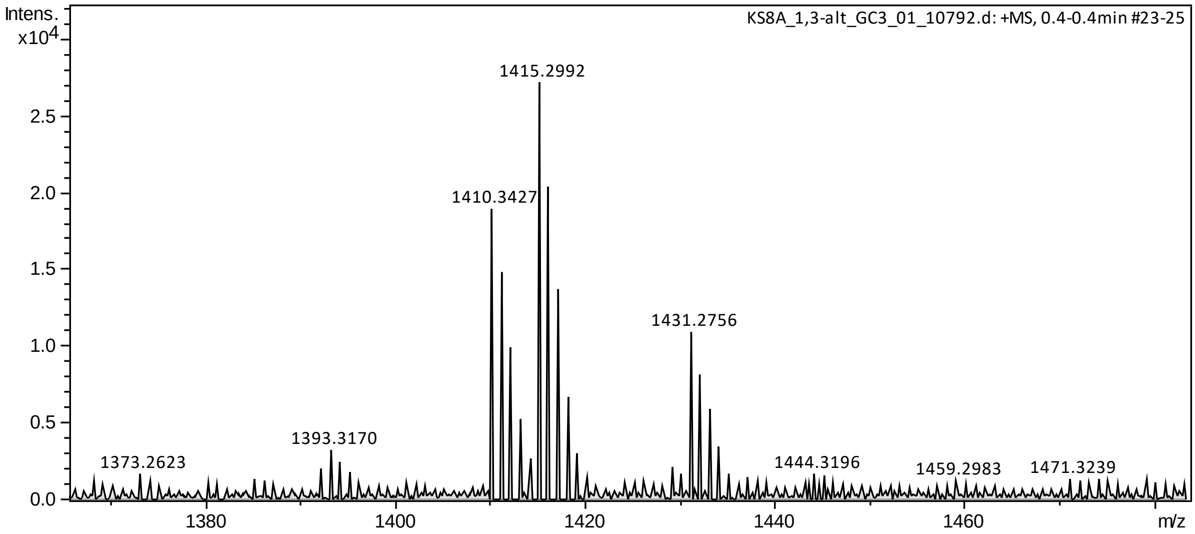

Fig. S79: HRMS of compound **8A** calc $[C_{64}H_{65}N_8O_{20}S_4]^+$ 1393.3193; found *m/z* 1393.3170 $[M+H]^+$, and calc $[C_{64}H_{64}N_8O_{20}S_4+Na]^+$ 1415.3012; found *m/z* 1415.2992 $[M+Na]^+$.

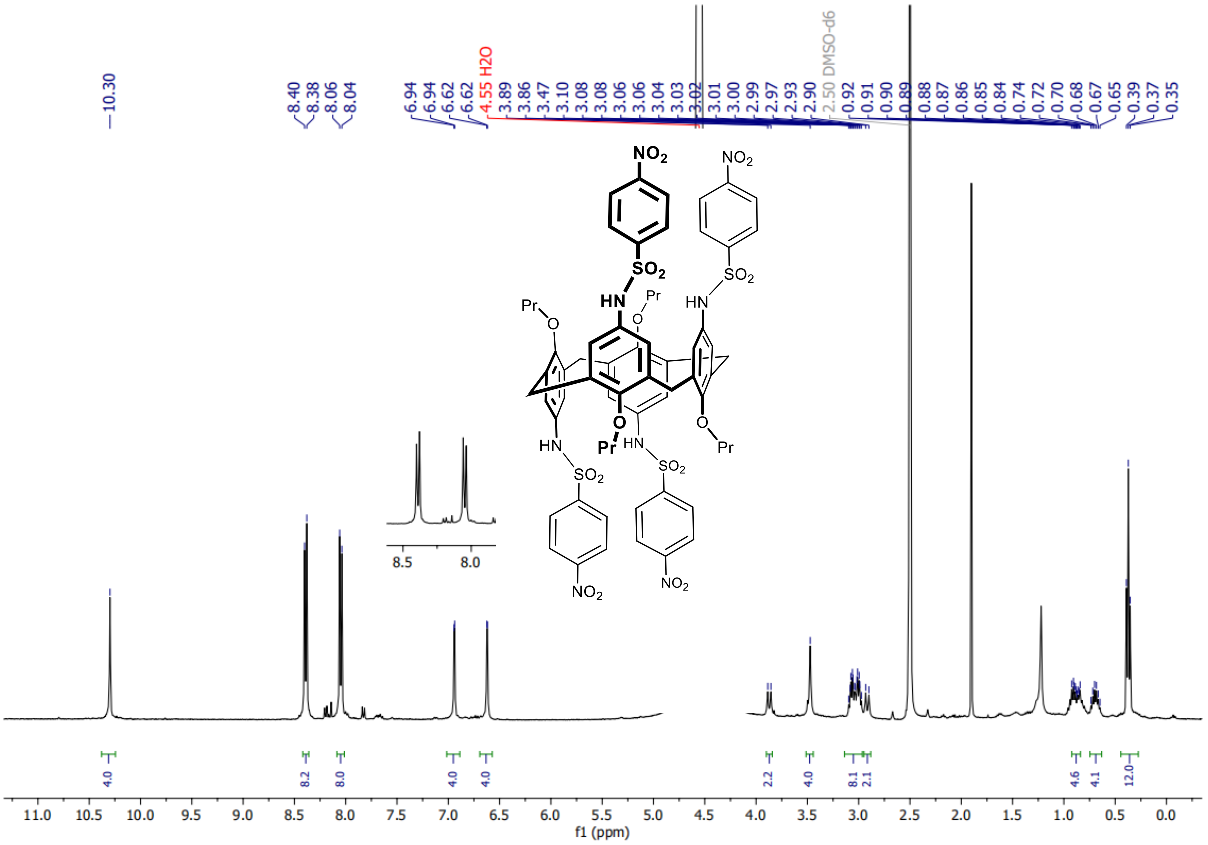

Fig S80: ^{1}H NMR of compound **9A** (DMSO-d_6, 400 MHz).

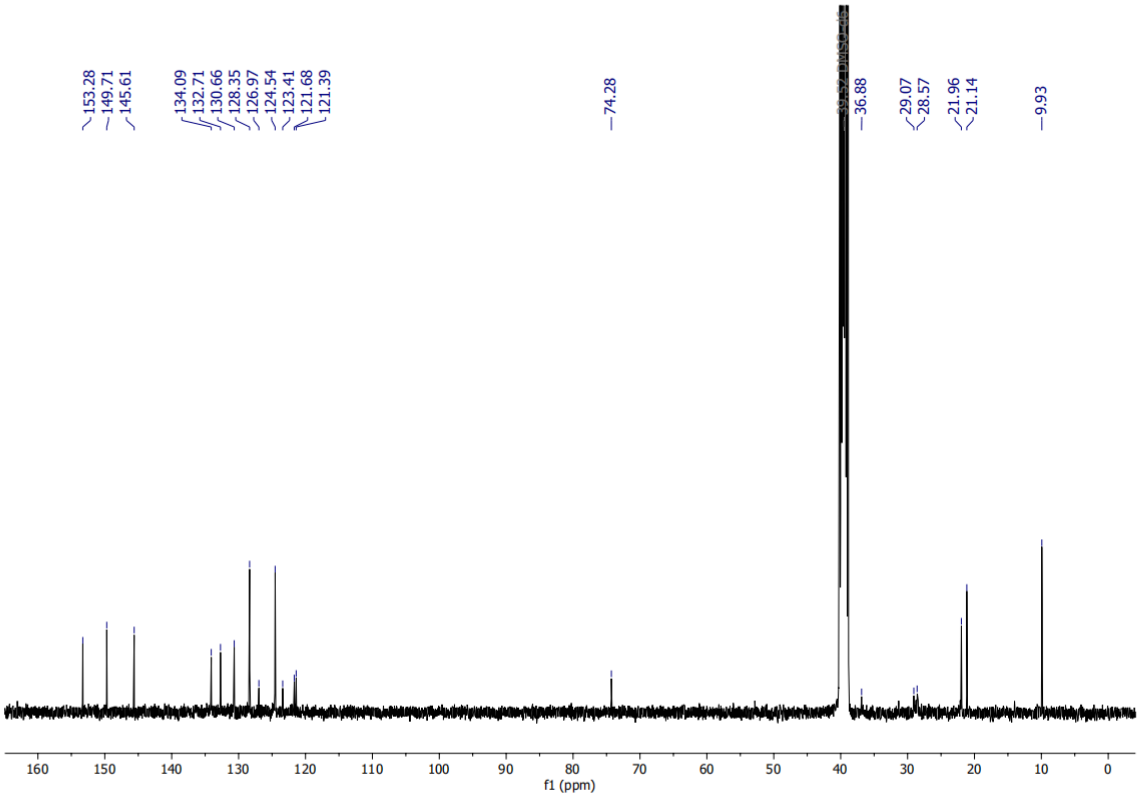

Fig. S81: $^{13}C\{^{1}H\}$ NMR of compound **9A** (DMSO-d_6, 100 MHz).

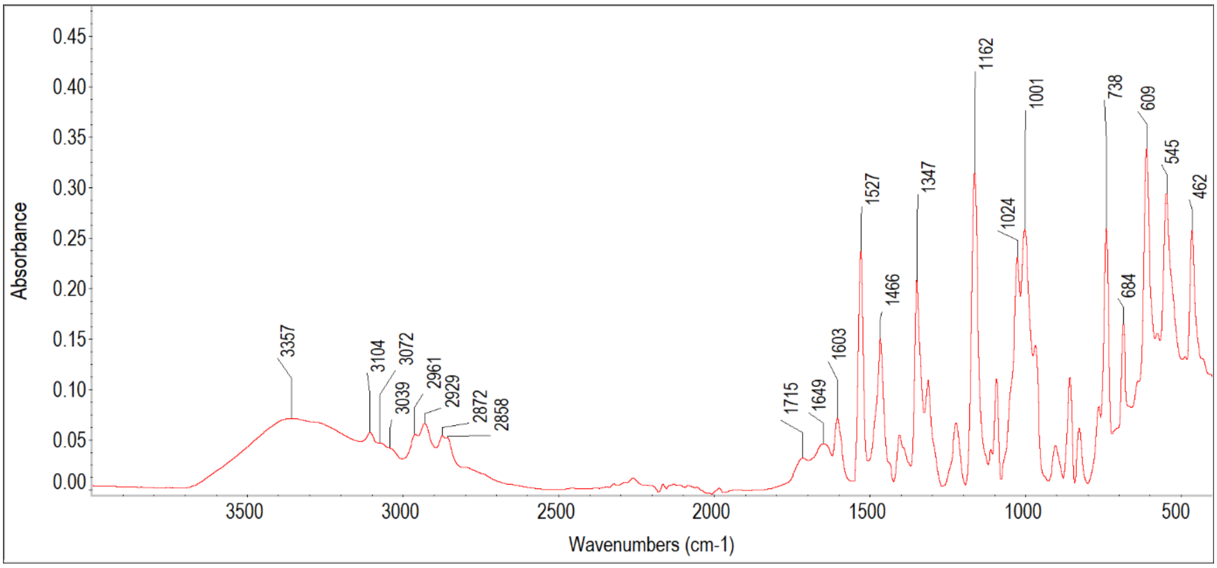

Fig. S82: IR spectrum of compound **9A**(ATR).

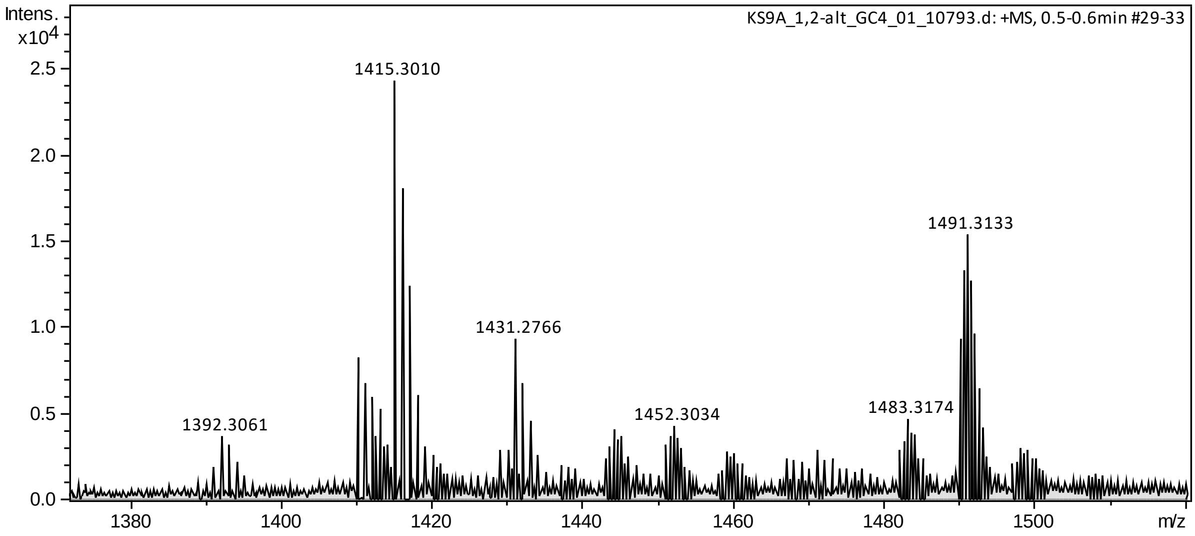

Fig. S83: HRMS of compound **9A** calc $[C_{64}H_{64}N_8O_{20}S_4]^+$ 1392.3119; found *m/z* 1392.3161 $[M]^+$, and calc $[C_{64}H_{64}N_8O_{20}S_4+Na]^+$ 1415.3012; found *m/z* 1415.3010 $[M+Na]^+$.

3. DFT calculations

Table S1: DFT optimized coordinates for structure **2a**.

Symbol	X	Y	Z
C	-1.027880	-3.682240	-0.656310
C	-0.965450	-2.290660	-0.699430
C	-0.035490	-1.597860	0.078097
C	0.839828	-2.308170	0.913609
C	0.779344	-3.690970	0.967879
C	-0.153140	-4.388810	0.181249
O	-0.126870	-5.740590	0.303654
C	-1.041270	-6.512410	-0.465760
N	0.026812	-0.163400	0.019479
S	-0.664410	0.767466	1.311462
S	0.798673	0.599033	-1.338930
O	-0.731850	-0.142000	2.450563
O	0.065157	2.030326	1.376415
C	-2.351920	1.100234	0.784914
O	0.822698	-0.418530	-2.384590
O	0.153183	1.893223	-1.539490
C	2.495961	0.874444	-0.812450
C	-2.612360	2.200462	-0.035920
C	-3.925300	2.450786	-0.424870
C	-4.931350	1.595558	0.020394
C	-4.678480	0.501399	0.843917
C	-3.365600	0.250590	1.234738
C	2.824103	2.051349	-0.135260
C	4.145248	2.254901	0.253531
C	5.091318	1.277577	-0.048330
C	4.771015	0.105083	-0.728270
C	3.450335	-0.098850	-1.119460
N	6.493174	1.495105	0.365060
O	7.306864	0.613888	0.095464
O	6.755367	2.542198	0.952391
N	-6.324290	1.861602	-0.394590
O	-7.191820	1.085583	0.001270
O	-6.526060	2.840233	-1.109890
H	-1.750410	-4.199940	-1.274650
H	-1.630280	-1.738410	-1.354210
H	1.550552	-1.766050	1.527166
H	1.441304	-4.258840	1.612273
H	-0.840910	-7.553340	-0.210800
H	-2.081340	-6.271880	-0.214250
H	-0.885030	-6.366180	-1.541370
H	-1.801010	2.835019	-0.369520
H	-4.178590	3.290019	-1.059760
H	-5.499020	-0.125570	1.168209
H	-3.126970	-0.578060	1.890694
H	2.057251	2.781578	0.089583
H	4.450053	3.150133	0.780010

H	5.547294	-0.617410	-0.944900
H	3.162300	-0.987440	-1.668410

Table S2: DFT optimized coordinates for structure **4b**.

Symbol	X	Y	Z
C	0.553131	-3.795610	0.627251
C	0.568759	-2.402720	0.675502
C	-0.327030	-1.656060	-0.092060
C	-1.246260	-2.313520	-0.923520
C	-1.263620	-3.697450	-0.982900
C	-0.366050	-4.448980	-0.205570
O	-0.469070	-5.797480	-0.331870
C	0.405058	-6.621060	0.429821
N	-0.309250	-0.220790	-0.028400
S	0.435317	0.675013	-1.321730
S	-1.027460	0.575557	1.334975
O	0.431092	-0.232270	-2.465090
O	-0.220290	1.978918	-1.373500
C	2.141278	0.898866	-0.808900
O	-1.108990	-0.443440	2.376977
O	-0.309880	1.830086	1.542368
C	-2.708830	0.952569	0.817758
C	2.476511	1.969536	0.022167
C	3.807104	2.130269	0.399360
C	4.776873	1.231245	-0.054170
C	4.426458	0.167337	-0.888860
C	3.098627	-0.004500	-1.273730
C	-2.969140	2.145146	0.138437
C	-4.277720	2.427723	-0.243240
C	-5.279970	1.510996	0.067696
C	-5.027320	0.323171	0.749578
C	-3.719040	0.040408	1.133365
C	6.224123	1.446486	0.312162
F	6.351273	2.038613	1.518170
F	6.845581	2.239792	-0.588380
F	6.906693	0.281883	0.352953
N	-6.668240	1.812411	-0.337660
O	-7.532960	0.983721	-0.059780
O	-6.870190	2.871663	-0.927280
H	1.250459	-4.355150	1.238102
H	1.268766	-1.890540	1.326143
H	-1.929700	-1.730090	-1.530080
H	-1.961000	-4.225080	-1.624180
H	0.145025	-7.648640	0.174273
H	1.455608	-6.439030	0.172401
H	0.264465	-6.468930	1.506851
H	1.708670	2.647536	0.373207
H	4.091185	2.949277	1.050279
H	5.187294	-0.523370	-1.234220
H	2.805864	-0.813070	-1.932990

H	-2.160210	2.825866	-0.094230
H	-4.530440	3.338141	-0.771240
H	-5.844740	-0.350140	0.973169
H	-3.481830	-0.862580	1.683119

4. Electrochemical study

4.1 Summary

Table S3: Comparison of key parameters for electrolysis of individual sulfonimides performed in a divided H-cells equipped with W = mercury pool, Ref = Ag/AgCl, Aux = Pt.

Compound	Peak potential (V) [a]	Applied potential (V) [b]	Time	*z* [c]	Yield [d]
**2a**	-0.71	-0.9	2 h 30 min	1.82	85
**2b**	-0.67	-0.9	2 h 20 min	1.94	87
**2c**	-0.59	-0.8	2 h 20 min	1.85	88
**3a**	-0.74	-0.9	2 h 40 min	1.92	87
**3b**	-1.92	-2.0	1 h 45 min	1.95	85
**3c**	-1.48	-1.6	1 h 30 min	1.72	80
**3d**	-2.08	-2.2	2 h 10 min	1.75	80
**4a**	-0.77	-0.9	3 h 10 min	1.92	87
**4b**	-0.72	-0.9	2 h 35 min	1.82	78
**4c**	-0.77	-0.9	3 h 30 min	1.90	89
**6**	-0.66	-0.8	3 h 5 min	3.74	81
**7**	-0.81	-0.9	5 h 10min	7.34	80
**8**	-0.71	-0.9	7 h 50 min	7.23	70
**9**	-0.70 and -0.81	-0.9	4 h 55 min	7.27	76

[a] Cathodic peak potentials correspond to the first irreversible (splitting) potential, obtained from corresponding cyclic voltammograms with the scan rates 100 mV/s.

[b] Potential applied during electrolysis.

[c] Number of electron z, $z = \frac{Q \cdot M}{F \cdot m}$, where *Q* = charge (C) obtained during electrolysis, *M* = molar mass (g/mol); *F* = Faraday constant = 96 485 C/mol; *m* = mass (g)

[d] Yields of obtained sulfonamides after acidic workup of crude electrolysed mixture and isolation of product by preparative TLC (with mobile phase PE:EA = 1:1).

4.2 Detail description of derivative 2a

4.2.1 Polarography and cyclic voltammetry

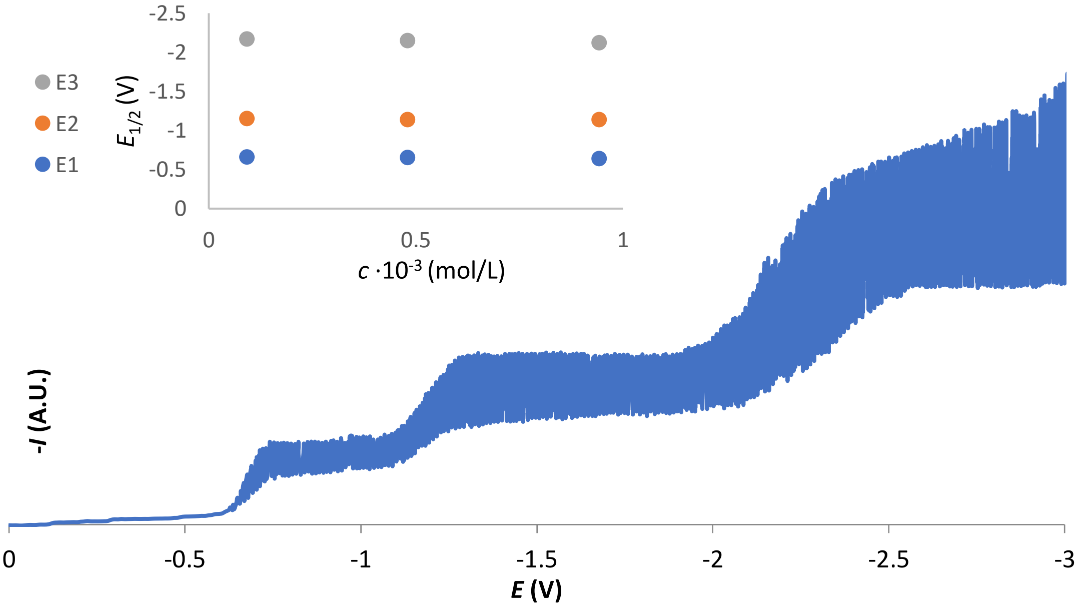

Fig. S84: Polarographic curve for $5{\cdot}10^{-4}$ M concentration of compound **2a** in DMSO (0.1 M $TBAPF_6$). In the cutout, there is the concentration dependence of obtained half-wave potentials from dc-polarography for **2a** (in DMSO with 0.1 M $TBAPF_6$).

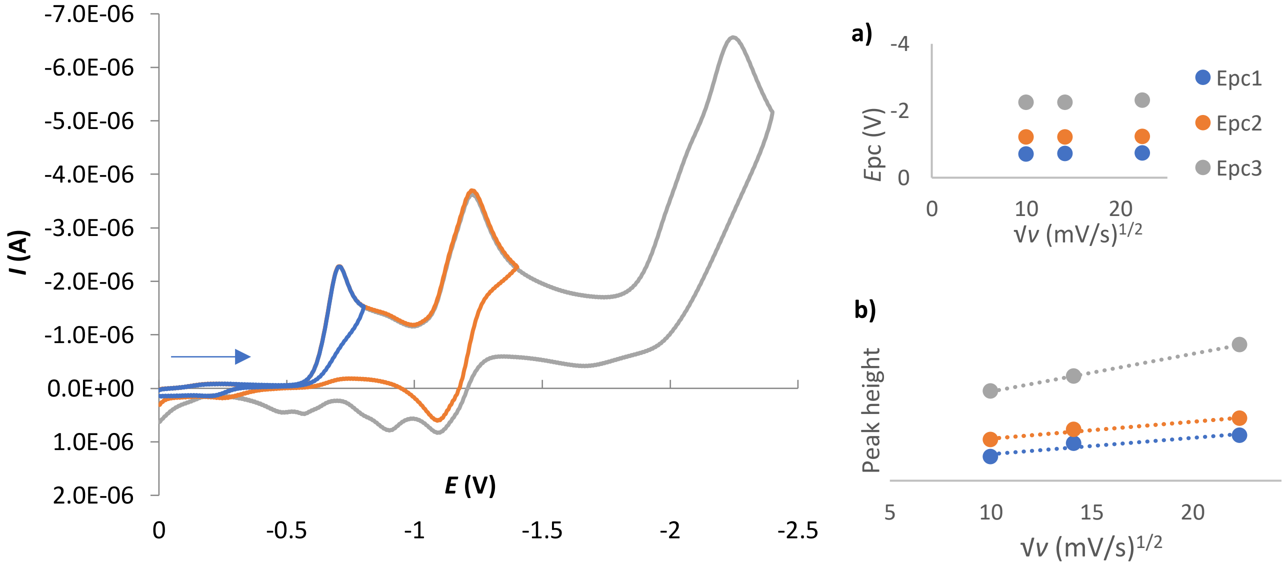

Fig. S85: Cyclic voltammogram (W = HMDE, Ref = SCE, Aux = Pt) of compound **2a** ($1{\cdot}10^{-3}$ M) in DMSO (0.1 M $TBAPF_6$) with scan rate 100 mV/s (polarographic plotting convention). In the cutout, there is a diagnostic of dependence of **a)** cathodic peak potentials on square root of the scan rate, **b)** peak height on square root of the scan rate.

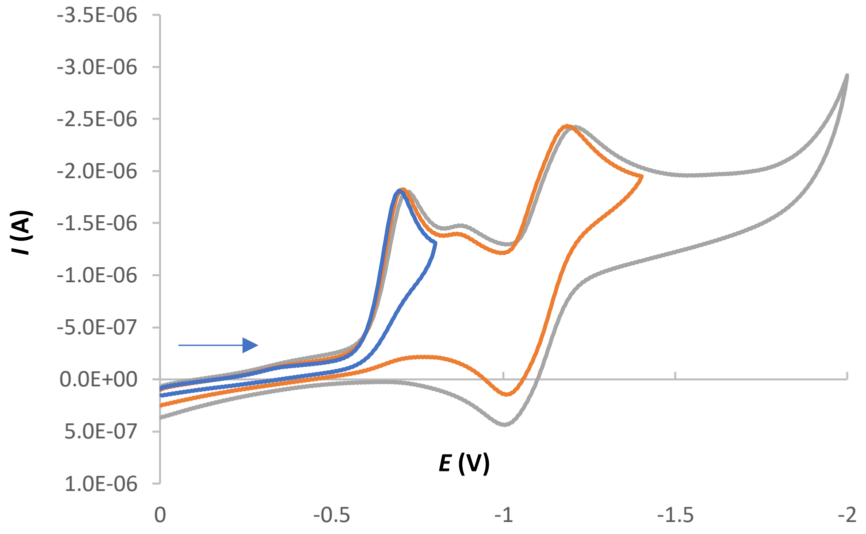

Fig. S86: Cyclic voltammogram (W = Pt disk electrode, Ref = SCE, Aux = Pt) of compound **2a** ($1{\cdot}10^{-3}$ M) in DMSO (0.1 M $TBAPF_6$) with scan rate 100 mV/s (polarographic plotting convention). Grey scan finished at potential -2 V due to limits of the potential window.

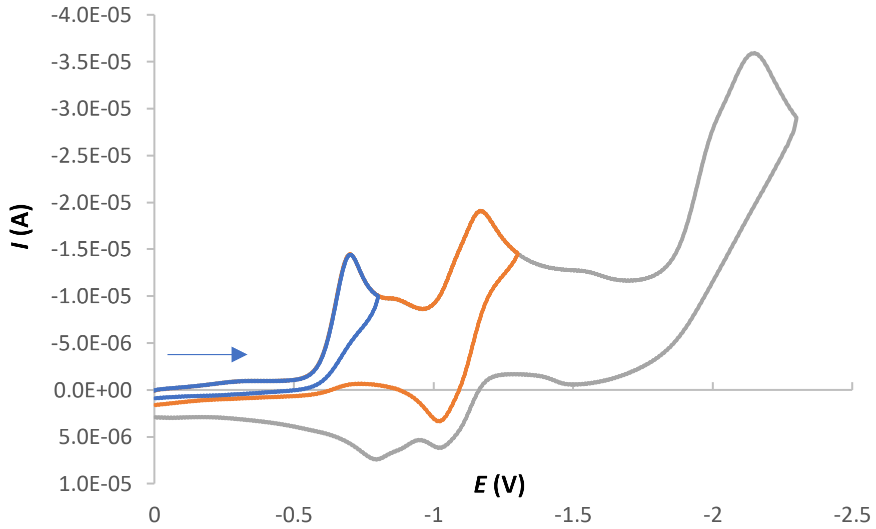

Fig. S87: Cyclic voltammogram (W = glassy carbon – ⌀ 3 mm, Ref = SCE, Aux = Pt) of **2a** ($1{\cdot}10^{-3}$ M) in DMSO (0.1 M $TBAPF_6$) with scan rate 100 mV/s (polarographic plotting convention).

4.2.2 Electrolysis

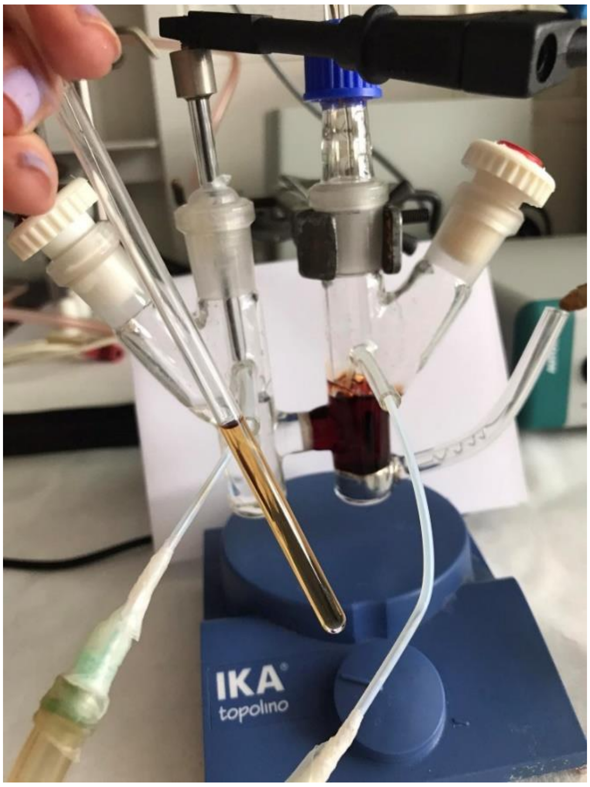

Fig. S88: Illustrative picture after electrolysis of **2a** in divides H-cell with mercury pool used as a working electrode (Ref = Ag/AgCl, Aux = Pt) with non-electrolyzed solution of **2a** in NMR cuvette.

a)

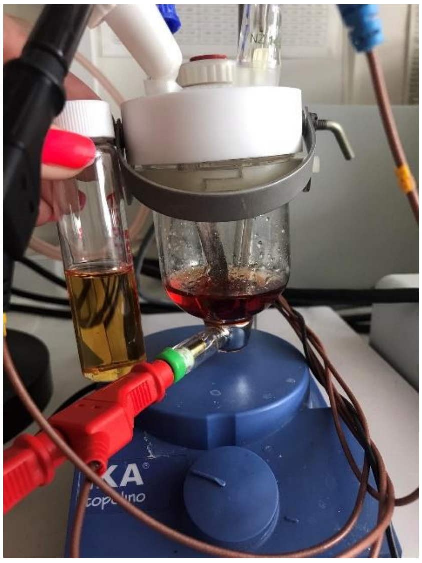

b)

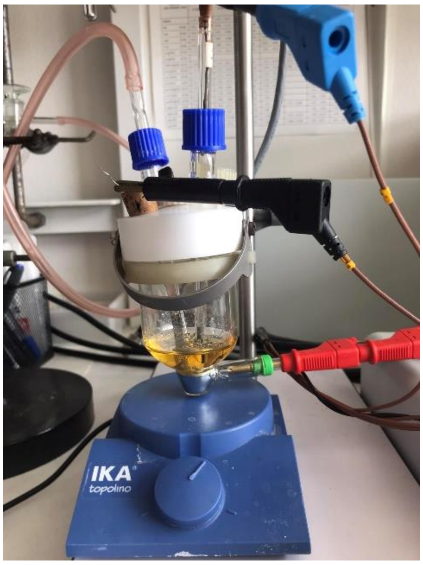

Fig. S89: Illustrative picture after batch electrolysis of **2a** in undivided cell with mercury pool used as a working electrode (Ref = Ag/AgCl) **a)** with Aux = Mg dissolving electrode, **b)** with Aux = Pt.

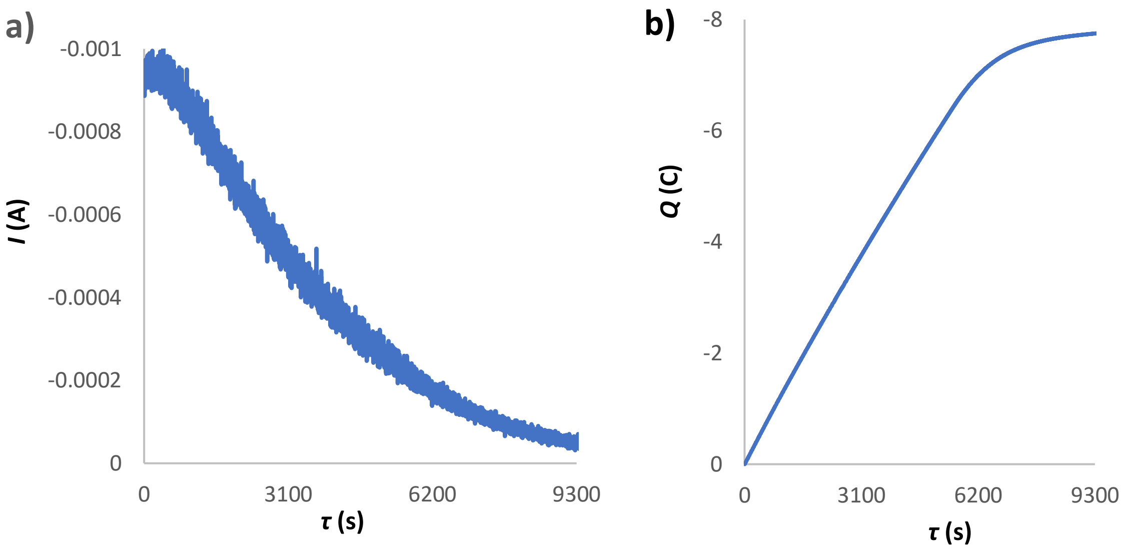

Fig. S90: a) Current record obtained during electrolysis of compound **2a** (10 mM) in DMSO-d_6 (60 mM KPF_6) with W = mercury pool, Ref = Ag/AgCl, Aux = Pt in divided H-cell. **b)** Charge record obtained during electrolysis of compound **2a** (10 mM) in DMSO-d_6 (60 mM KPF_6) with W = mercury pool, Ref = Ag/AgCl, Aux = Pt in divided H-cell.

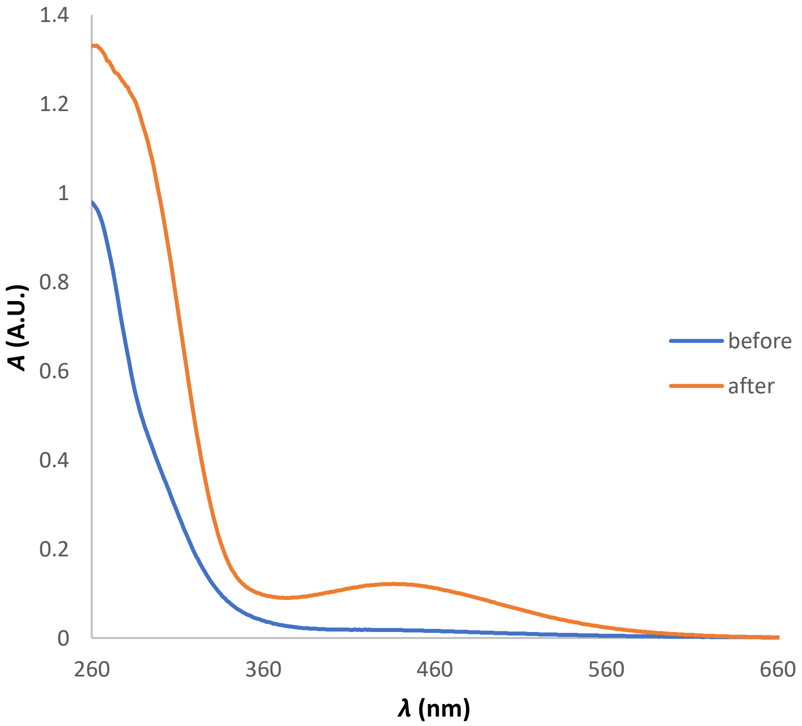

Fig. S91: UV-Vis spectra of crude solution of compound **2a** diluted to the concentration (0.1 mmol/L) before **(blue)**, and after **(orange)** electrolysis. The formation of anionic species after electrochemical cleavage in the mixture leads to the appearance of a new band around 450 nm.

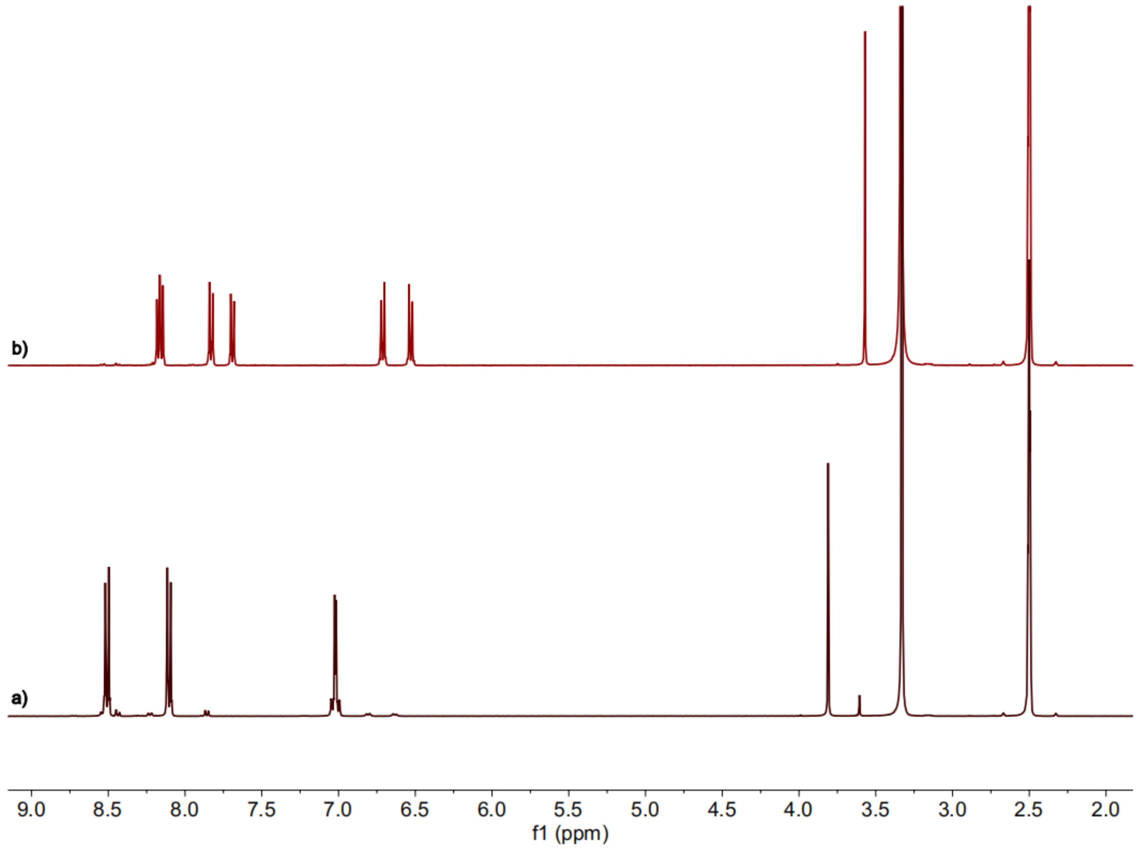

Fig. S92: ^{1}H NMR spectra of crude solution (10 mM **2a**, 60 mM KPF_6, DMSO-d_6) **a)** before, **b)** after electrolysis.

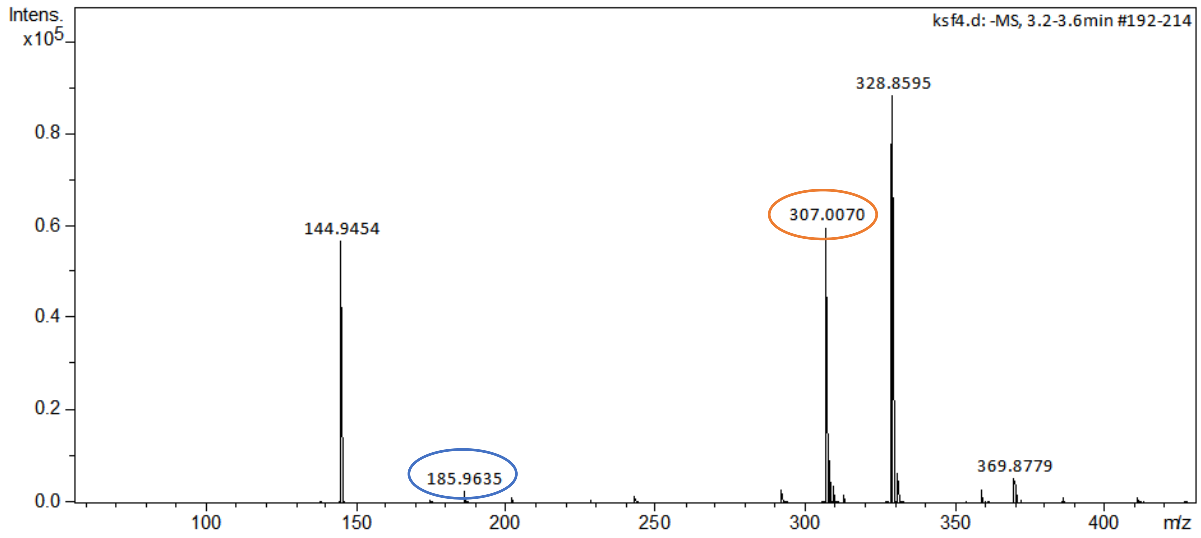

Fig. S93: HRMS spectra (ESI negative mode, MeOH) of crude electrolysed solution of **2a** containing peaks corresponding to nitrobenzenesulfinate **B** $[C_6H_4NO_4S]^-$: calc $[M]^-$ 185.9861, and anionic form of **2Xa** $[C_{13}H_{11}N_2O_5S]^-$: calc $[M]^-$ 307.0389.

4.2.3 Further electrochemical investigation

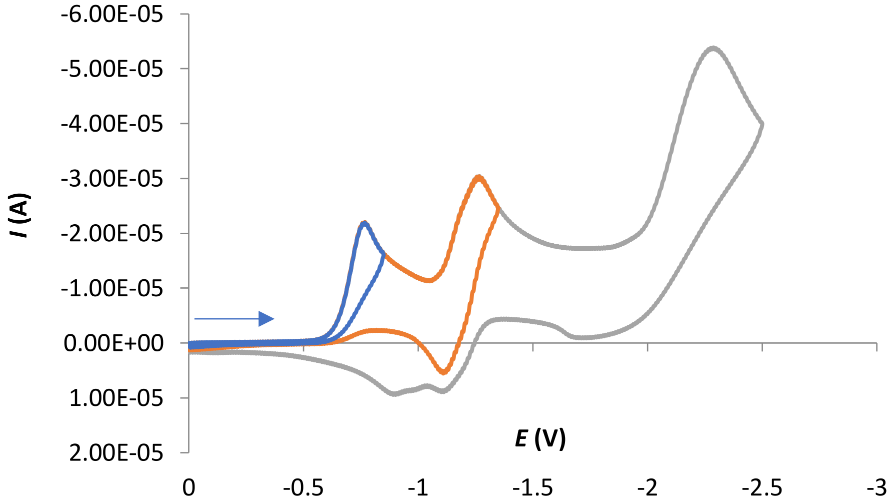

Fig. S94: Cyclic voltammogram (W = glassy carbon - ⌀ 1 mm, Ref = SCE, Aux = Pt) of **2a** ($1 \cdot 10^{-3}$ M) in DMF (0.1 M $TBAPF_6$) with scan rate 100 mV/s (polarographic plotting convention) at room temperature.

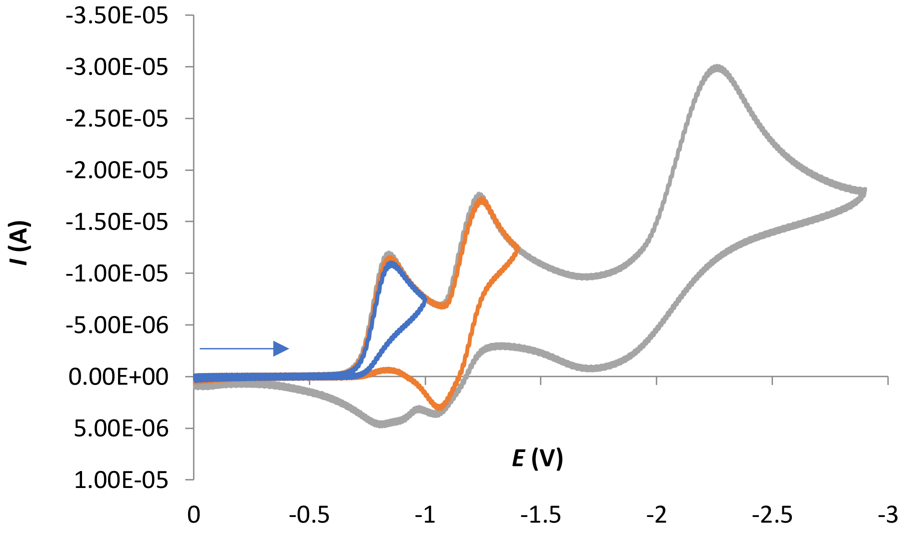

Fig. S95: Cyclic voltammogram (W = glassy carbon - ⌀ 1 mm, Ref = SCE, Aux = Pt) of **2a** ($1 \cdot 10^{-3}$ M) in DMF (0.1 M $TBAPF_6$) with scan rate 100 mV/s (polarographic plotting convention) at temperature -44 °C. According to this record the mechanism of reduction is independent on the temperature (only the kinetics is affected).

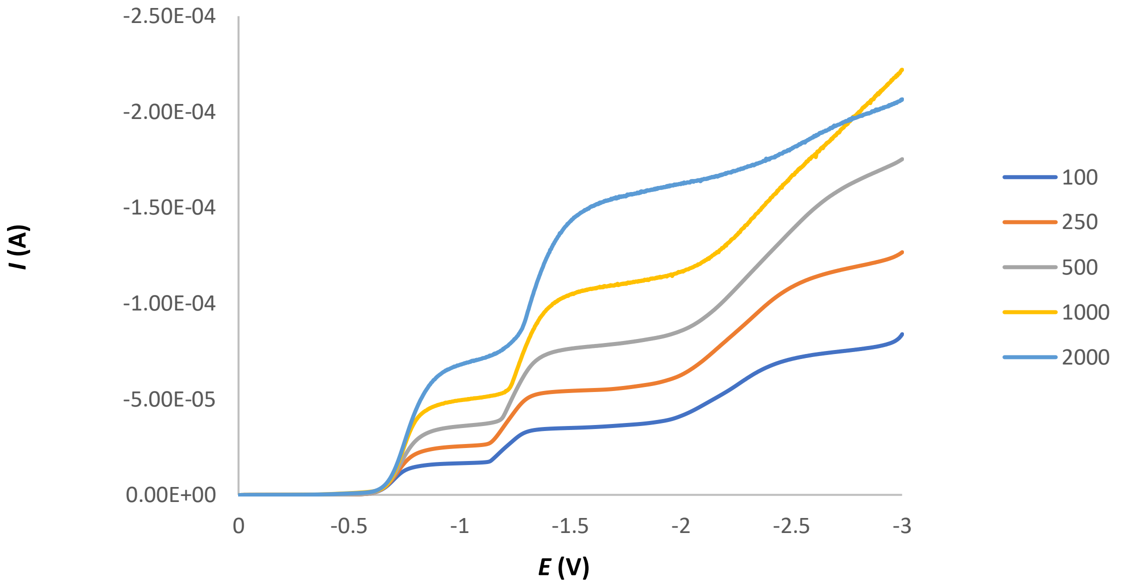

Fig S96: Linear sweep voltammetry of **2a** ($1 \cdot 10^{-3}$ M) in DMF (0.1 M $TBAPF_6$) (W = RDE = glassy carbon - ⌀ 1 mm, Ref = SCE, Aux = Pt) was measured with scan rate 10 $mV \cdot s^{-1}$ using several rotating rates (100, 250, 500, 1000, 2000 s^{-1}).

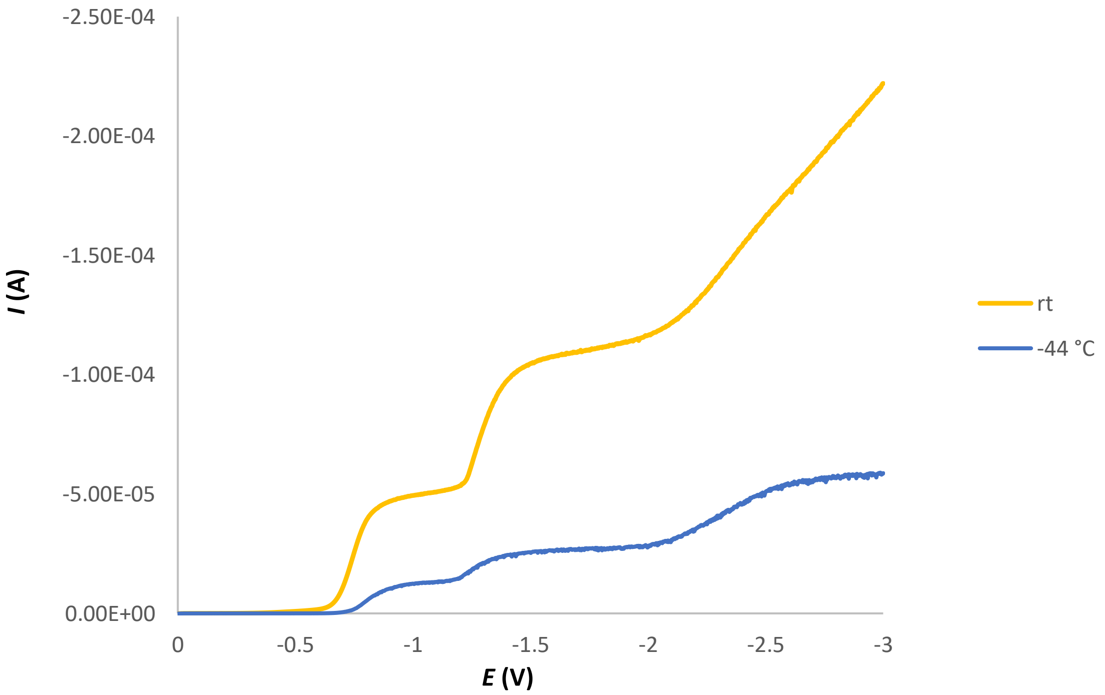

Fig S97: Linear sweep voltammetry of **2a** ($1 \cdot 10^{-3}$ M) in DMF (0.1 M TBAPF6) (W = RDE = glassy carbon - ⌀ 1 mm, Ref = SCE, Aux = Pt) was measured with scan rate of 10 $mV \cdot s^{-1}$, using rotating rate of 1000 s^{-1} for two different temperatures.

4.3 Cyclic voltammograms

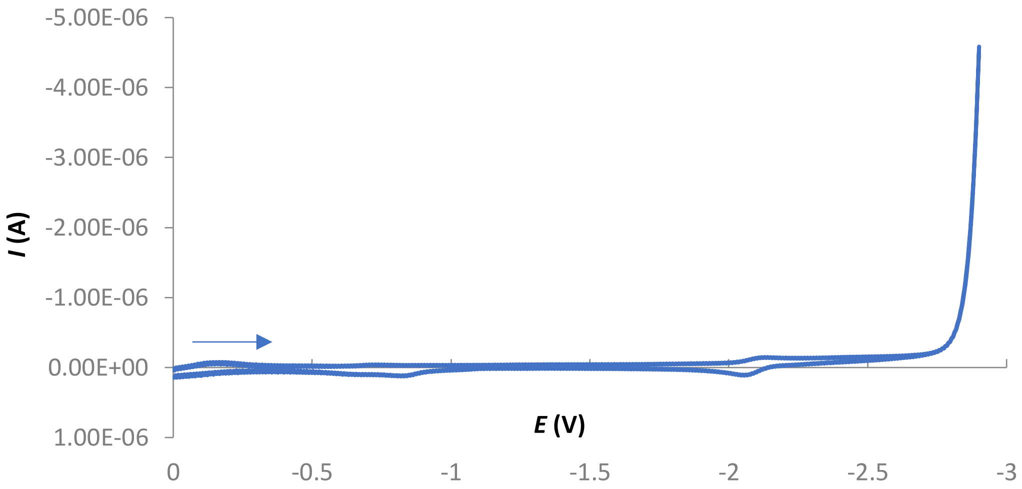

a)

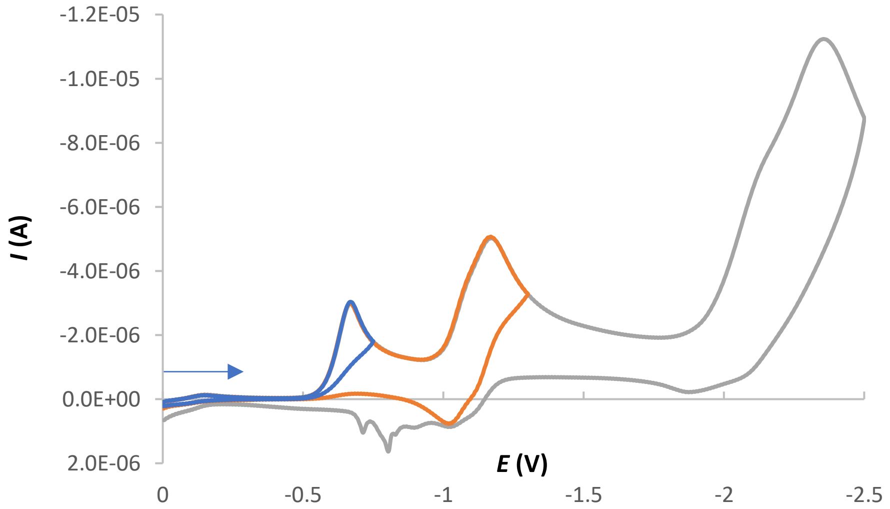

b)

Fig. S98: Cyclic voltammogram (W = HMDE, Ref = SCE, Aux = Pt) a) of typical blank of DMSO (0.1 M $TBAPF_6$) with scan rate 100 mV/s; b) of compound **2b** ($1\cdot10^{-3}$ M) in DMSO (0.1 M $TBAPF_6$) with scan rate 100 mV/s (polarographic plotting convention).

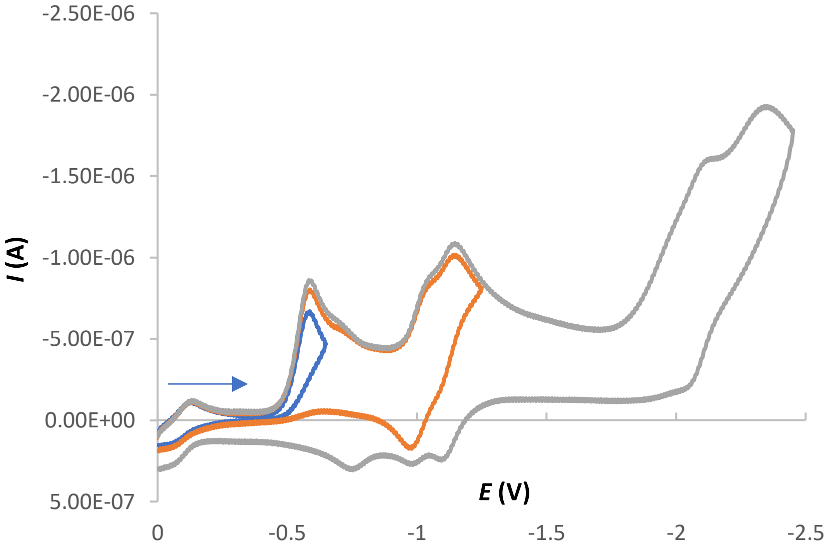

Fig. S99: Cyclic voltammogram (W = HMDE, Ref = SCE, Aux = Pt) of compound **2c** ($1{\cdot}10^{-3}$ M) in DMSO (0.1 M $TBAPF_6$) with scan rate 100 mV/s (polarographic plotting convention).

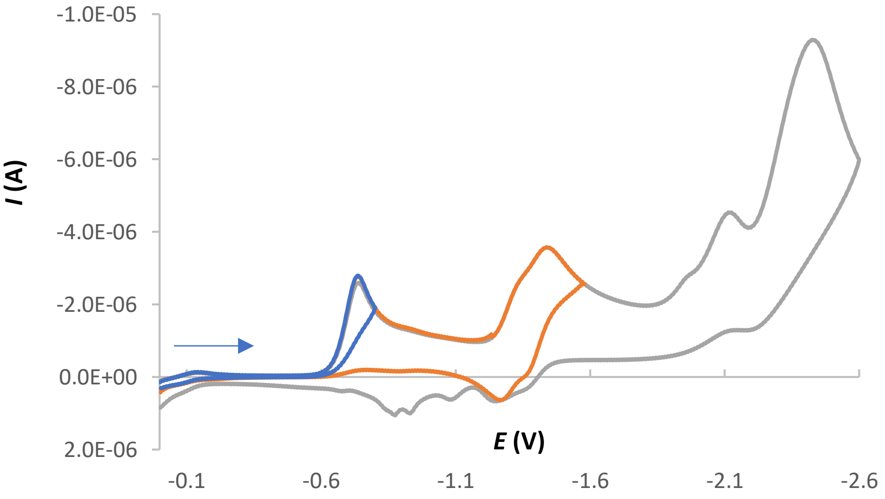

Fig. S100: Cyclic voltammogram (W = HMDE, Ref = SCE, Aux = Pt) of compound **3a** ($1{\cdot}10^{-3}$ M) in DMSO (0.1 M $TBAPF_6$) with scan rate 100 mV/s (polarographic plotting convention).

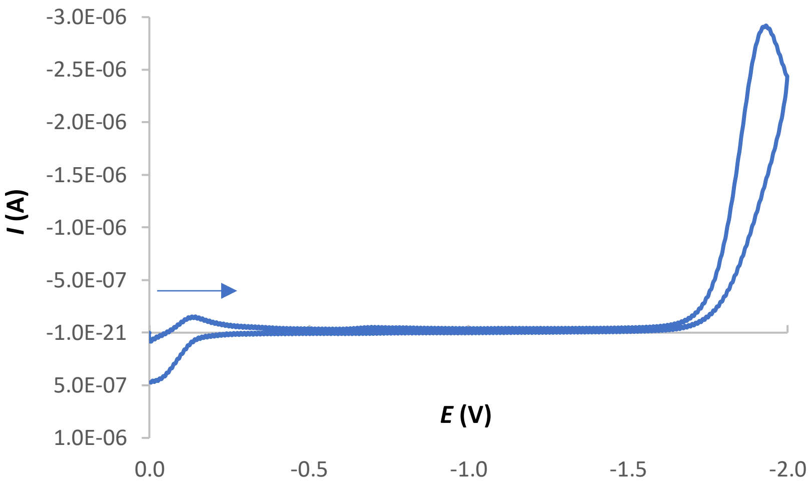

Fig. S101: Cyclic voltammogram (W = HMDE, Ref = SCE, Aux = Pt) of compound **3b** ($1{\cdot}10^{-3}$ M) in DMSO (0.1 M $TBAPF_6$) with scan rate 100 mV/s (polarographic plotting convention).

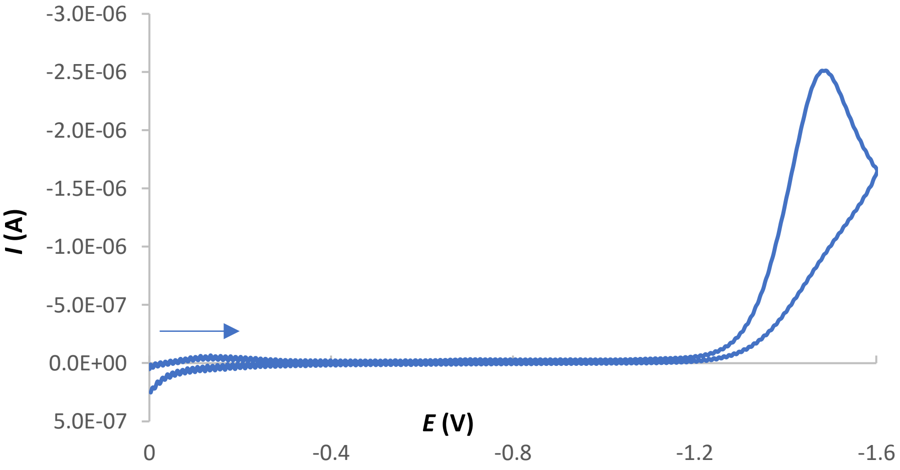

Fig. S102: Cyclic voltammogram (W = HMDE, Ref = SCE, Aux = Pt) of compound **3c** ($1{\cdot}10^{-3}$ M) in DMSO (0.1 M $TBAPF_6$) with scan rate 100 mV/s (polarographic plotting convention).

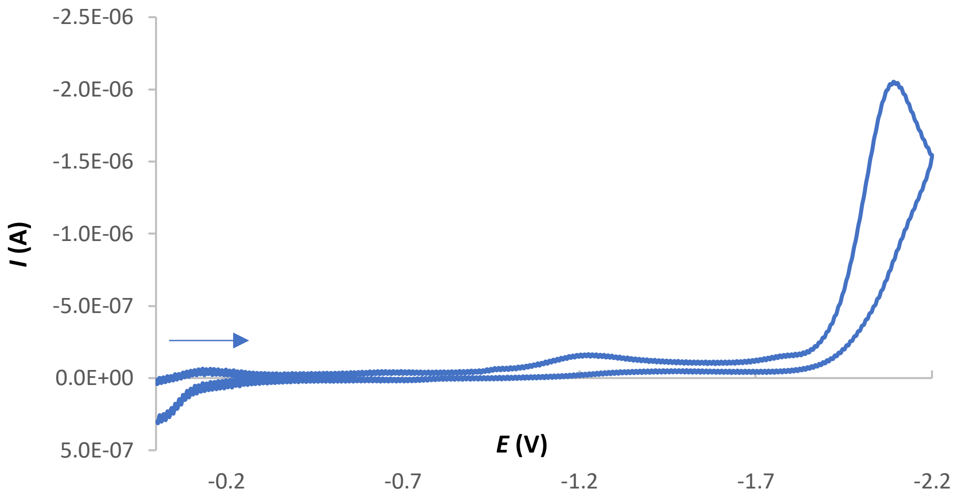

Fig. S103: Cyclic voltammogram (W = HMDE, Ref = SCE, Aux = Pt) of compound **3d** ($1 \cdot 10^{-3}$ M) in DMSO (0.1 M $TBAPF_6$) with scan rate 100 mV/s (polarographic plotting convention).

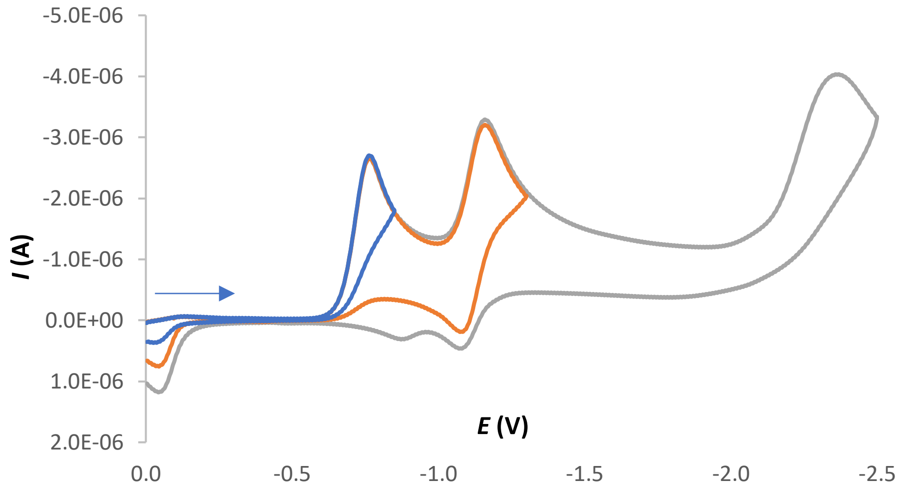

Fig. S104: Cyclic voltammogram (W = HMDE, Ref = SCE, Aux = Pt) of compound **4a** ($1 \cdot 10^{-3}$ M) in DMSO (0.1 M $TBAPF_6$) with scan rate 100 mV/s (polarographic plotting convention).

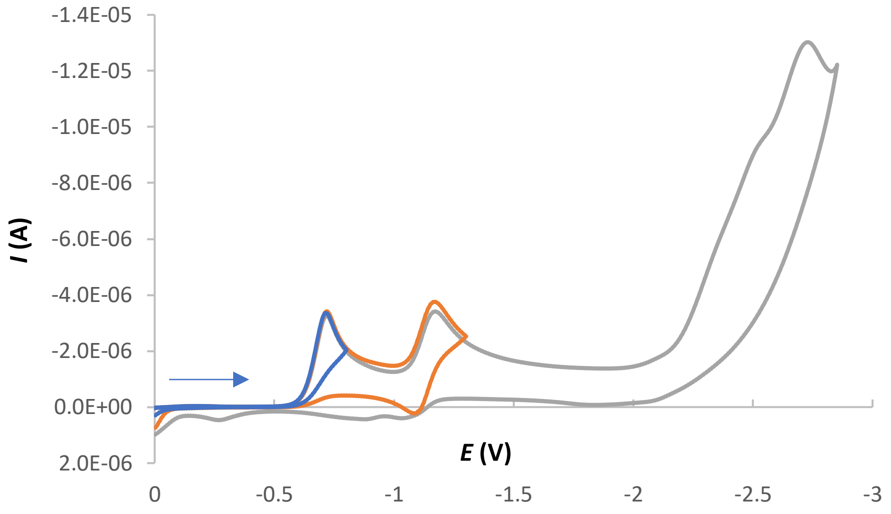

Fig. S105: Cyclic voltammogram (W = HMDE, Ref = SCE, Aux = Pt) of compound **4b** ($1\cdot10^{-3}$ M) in DMSO (0.1 M $TBAPF_6$) with scan rate 100 mV/s (polarographic plotting convention).

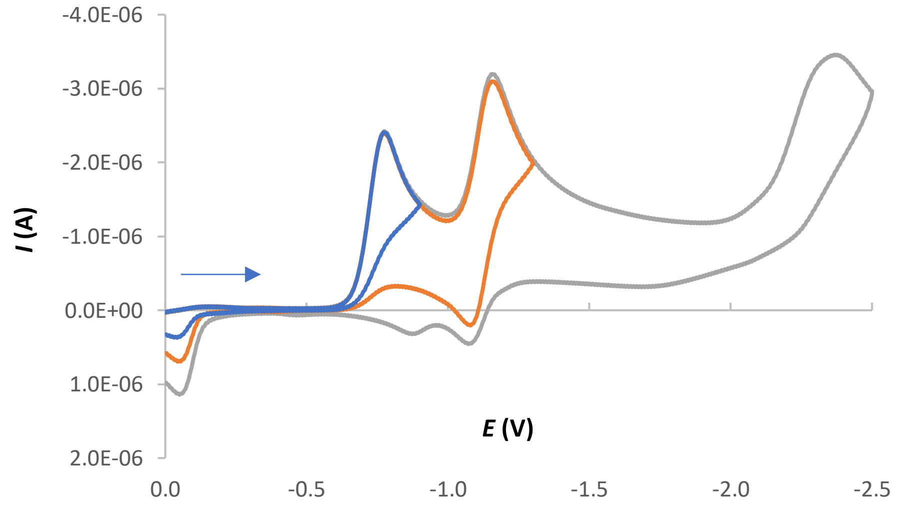

Fig. S106: Cyclic voltammogram (W = HMDE, Ref = SCE, Aux = Pt) of compound **4c** ($1\cdot10^{-3}$ M) in DMSO (0.1 M $TBAPF_6$) with scan rate 100 mV/s (polarographic plotting convention).

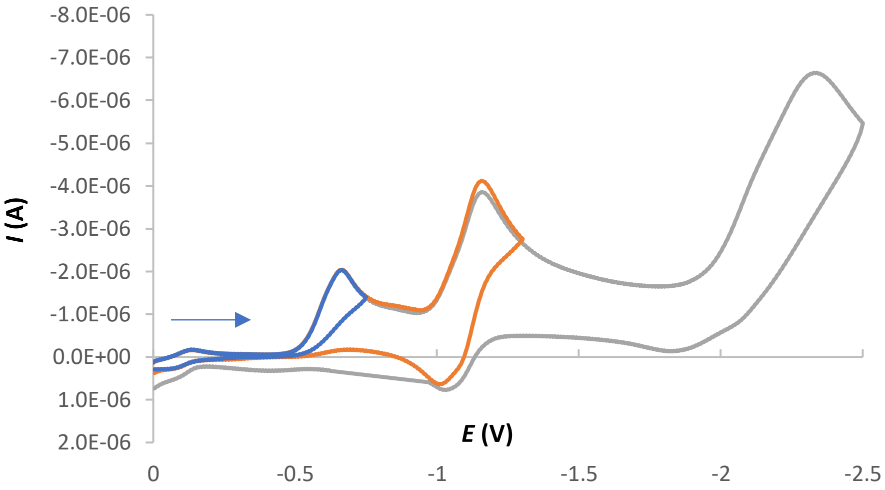

Fig. S107: Cyclic voltammogram (W = HMDE, Ref = SCE, Aux = Pt) of compound **6** ($5\cdot10^{-4}$ M) in DMSO (0.1 M $TBAPF_6$) with scan rate 100 mV/s (polarographic plotting convention).

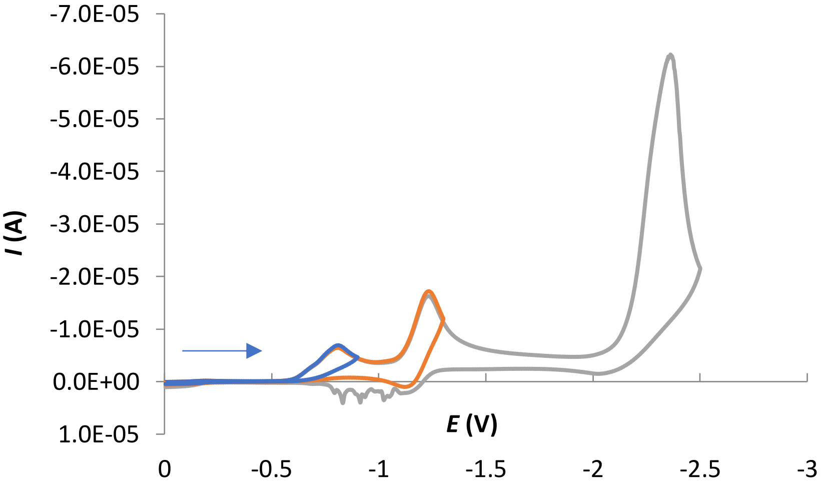

Fig. S108: Cyclic voltammogram (W = HMDE, Ref = SCE, Aux = Pt) of compound **7** ($5\cdot10^{-4}$ M) in DMSO (0.1 M $TBAPF_6$) with scan rate 100 mV/s (polarographic plotting convention).

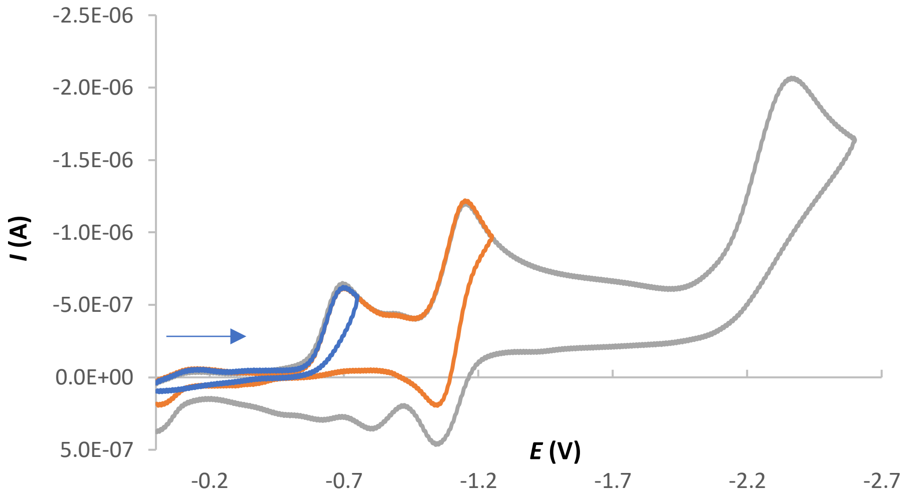

Fig. S109: Cyclic voltammogram (W = HMDE, Ref = SCE, Aux = Pt) of compound **8** ($1{\cdot}10^{-4}$ M) in DMSO (0.1 M $TBAPF_6$) with scan rate 100 mV/s (polarographic plotting convention).

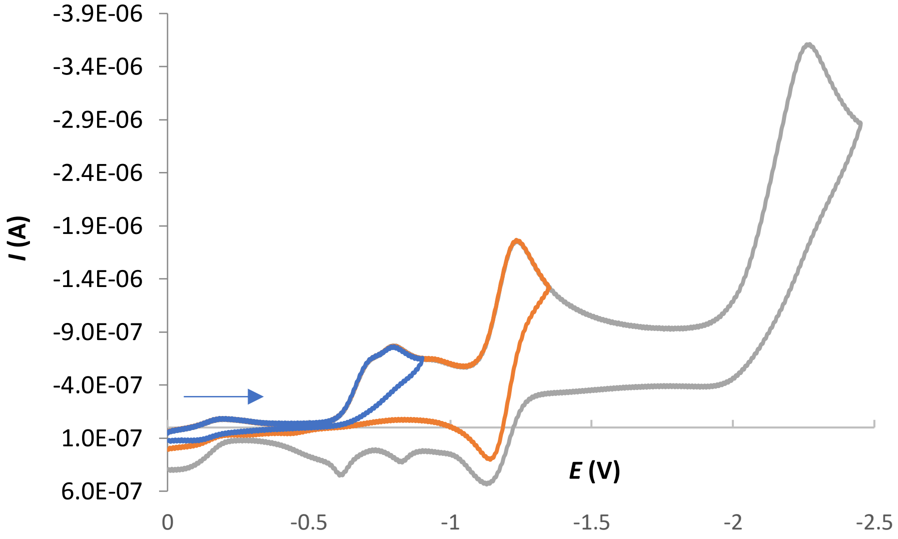

Fig. S110: Cyclic voltammogram (W = HMDE, Ref = SCE, Aux = Pt) of compound **9** ($1{\cdot}10^{-4}$ M) in DMSO (0.1 M $TBAPF_6$) with scan rate 100 mV/s (polarographic plotting convention).

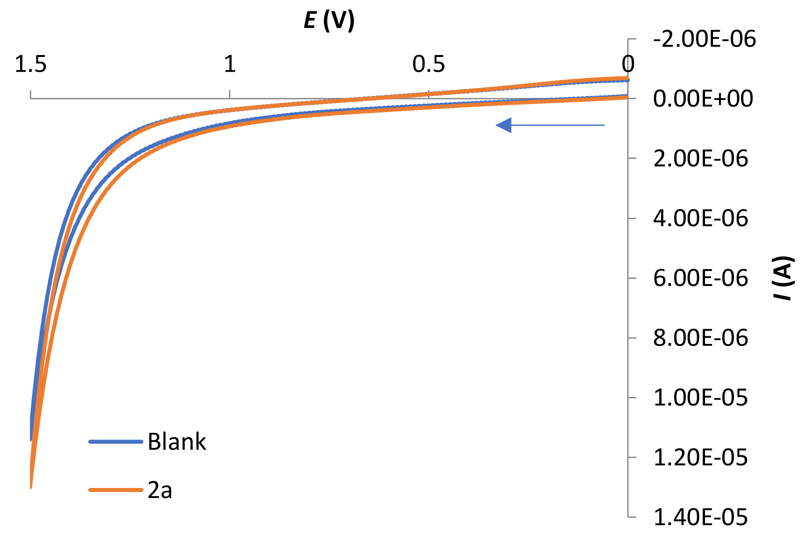

Fig. S111: Cyclic voltammogram (W = Pt, Ref = SCE, Aux = Pt) of compound **2a** ($5\cdot10^{-3}$ M) in DMSO (0.1 M $TBAPF_6$) with scan rate 100 mV/s (polarographic plotting convention). Confirming that no oxidation of sulfonimide proceeding.

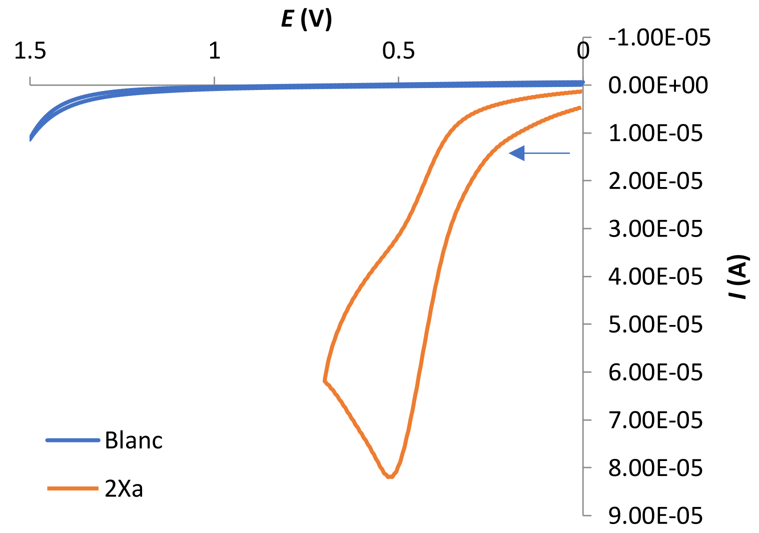

Fig. S112: Cyclic voltammogram (W = Pt, Ref = SCE, Aux = Pt) of compound **2Xa** ($5\cdot10^{-3}$ M) in DMSO (0.1 M $TBAPF_6$) with scan rate 100 mV/s (IUPAC plotting convention). Confirming the unwanted oxidation of deprotonated sulfonamide **2Xa**.

4.4 Mixture containing sulfonimide with sulfonamide

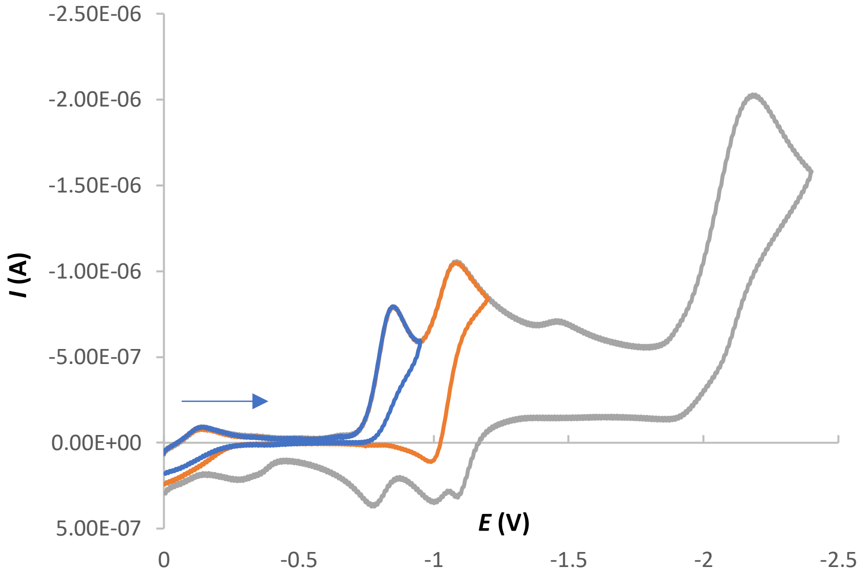

Fig. S113: Cyclic voltammogram (W = HMDE, Ref = SCE, Aux = Pt) of compound pure **2Ab** ($1{\cdot}10^{-3}$ M) in DMSO (0.1 M $TBAPF_6$) with scan rate 100 mV/s (polarographic plotting convention).

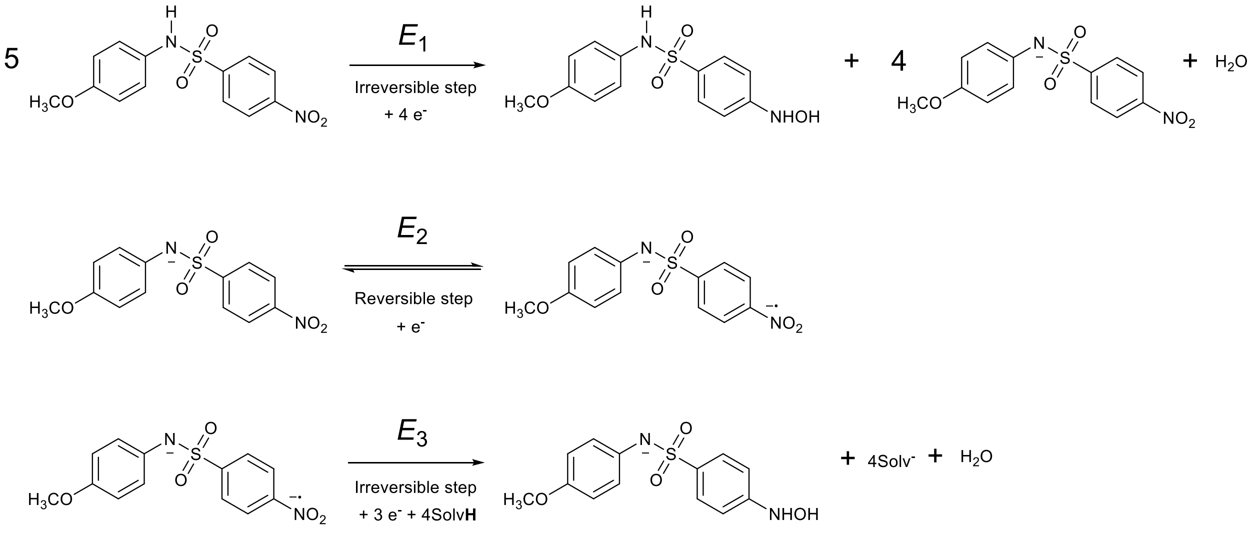

Scheme S1: Auto-protonation reduction mechanism describing the reduction of **2Ab** during three reduction steps obtained by CV above (Fig S113).

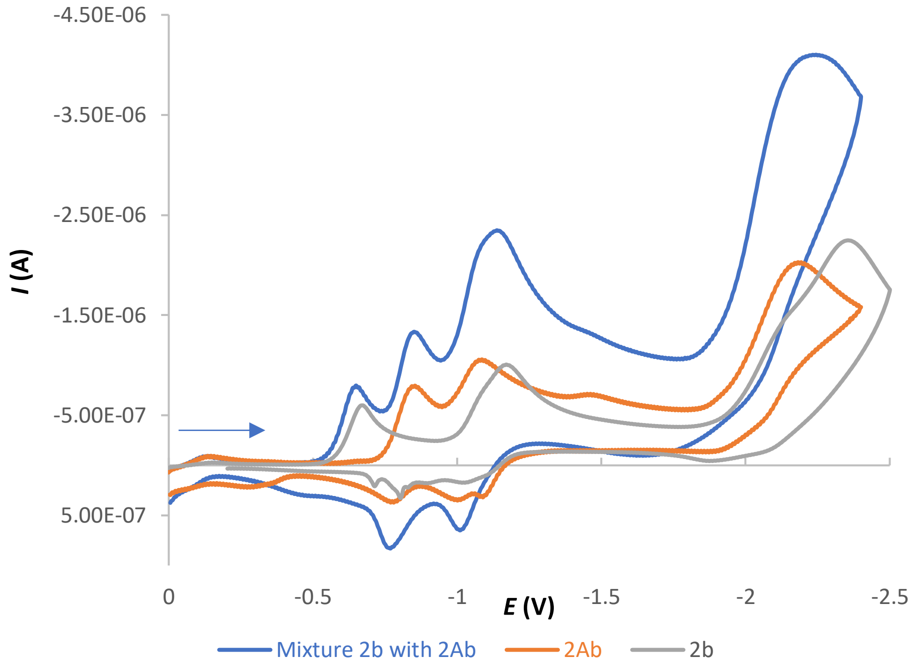

Fig. S114: a) grey Cyclic voltammogram (W = HMDE, Ref = SCE, Aux = Pt) of compound **2b** ($2\cdot10^{-4}$ M) in DMSO (0.1 M $TBAPF_6$) with scan rate 100 mV/s (polarographic plotting convention). **b) orange** Cyclic voltammogram (W = HMDE) of compound **2Ab** ($5.5\cdot10^{-4}$ M) in DMSO (0.1 M $TBAPF_6$) with scan rate 100 mV/s. **c) blue** Cyclic voltammogram (W = HMDE) of mixture containing compound **2b** ($2.7\cdot10^{-4}$ M), and compound **2Ab** ($5.5\cdot10^{-4}$ M) in DMSO (0.1 M $TBAPF_6$) with scan rate 100 mV/s.

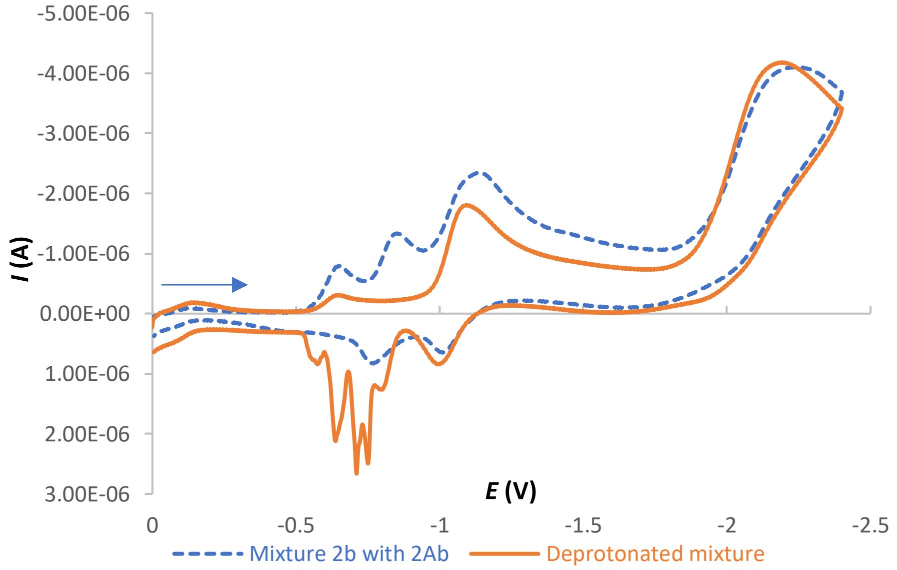

Fig. S115: a) blue Cyclic voltammogram (W = HMDE, Ref = SCE, Aux = Pt) of mixture containing compound **2b** ($2.7\cdot10^{-4}$ M), and compound **2Ab** ($5.5\cdot10^{-4}$ M) in DMSO (0.1 M $TBAPF_6$) with scan rate 100 mV/s (polarographic plotting convention). **b) orange** Cyclic voltammogram (W = HMDE) of same mixture after addition of 1 equiv. of TBAOH.
